# Supplementary material for: Clip-plate versus suture-anchor in double-door laminoplasty for degenerative cervical myelopathy: Protocol for a multicenter, non-inferiority, randomized controlled trial
Source: PLoS One. 2026 Apr 24;21(4):e0339103. doi: 10.1371/journal.pone.0339103 (PMC13108724; doi:10.1371/journal.pone.0339103)
Supplement: S2 File — (DOCX) [file pone.0339103.s002.docx]

両開き式頚椎椎弓形成術におけるプレート法と縫合糸アンカー法の

有用性に関する無作為化非劣性比較試験

臨床研究計画書

I2024-243

研究代表者：　吉井　俊貴

東京科学大学病院　整形外科

所在地：〒113-8519　東京都文京区湯島1-5-45

TEL： 03-5803-5279

FAX： 03-5803-0142

e-mail: yoshii.orth@tmd.ac.jp

2025年2月27日　　第１版

2025年10月17日　第1.1版

(受付番号追記、jRCT公表日による研究実施期間修正)

**略語一覧**

| 略語 | 説明 | |
| --- | --- | --- |
| ASA | American Society of Anesthesiologists | アメリカ麻酔科学会 |
| CSM | cervical spondylotic myelopathy | 頚椎症性脊髄症 |
| C-SVA | Cervical sagittal vertical axis | 頚椎矢状面垂直軸 |
| CTCAE | Common Terminology Criteria for Adverse Events | 有害事象共通用語基準 |
| DCM | degenerative cervical myelopathy | 変性頚部脊髄症 |
| FAS | Full analysis set | 最大の解析対象集団 |
| ICER | Incremental cost-effectiveness ratio | 増分費用効果比 |
| JOA | Japanese Orthopaedic Association | 日本整形外科学会 |
| jRCT | Japan Registry of Clinical Trials | 臨床研究等提出・公開システム  （臨床試験情報を提供するウェブサイト） |
| MCID | Minimal clinically important difference | 臨床的に意義のある最小変化量 |
| NDI | Neck Disability Index | 頚椎障害指数 |
| OPLL | Ossification of the Posterior Longitudinal Ligament | 後縦靭帯骨化症 |
| PPS | Per protocol set | 研究計画書に適合した対象集団 |
| QOL | Quality of Life | 生活の質 |
| SAS | Safety analysis set | 安全性解析対象集団 |

**目次**

[１．概要 6](#_Toc191420878)

[２．臨床研究の実施体制 8](#_Toc191420879)

[３．臨床研究の背景 9](#_Toc191420880)

[3.1 対象疾患について 9](#_Toc191420881)

[3.2 対象疾患に対する標準治療について 10](#_Toc191420882)

[3.3 研究デザインの設定根拠 12](#_Toc191420883)

[3.4 本研究の意義 12](#_Toc191420884)

[４．臨床研究の目的 12](#_Toc191420885)

[５．臨床研究の内容に関する事項 12](#_Toc191420886)

[5.1　主要評価項目および副次評価項目 12](#_Toc191420887)

[5.2　臨床研究のデザインおよびアウトライン 15](#_Toc191420888)

[5.2.1　臨床研究のデザイン 15](#_Toc191420889)

[5.2.2　臨床研究のアウトライン 16](#_Toc191420890)

[5.3　症例登録・割付方法 16](#_Toc191420891)

[5.4　研究対象者の参加予定期間 17](#_Toc191420892)

[5.5　臨床研究全体の中止基準 17](#_Toc191420893)

[６．研究対象者の選択および除外関する基準 17](#_Toc191420894)

[6.1　研究対象者（対象疾患） 17](#_Toc191420895)

[6.2　選択基準 17](#_Toc191420896)

[6.3　除外基準 18](#_Toc191420897)

[７．研究対象者に対する治療に関する事項 18](#_Toc191420898)

[7.1　プロトコール治療の実施手順 18](#_Toc191420899)

[7.1.1　同意取得 18](#_Toc191420900)

[7.1.2　登録・割付 19](#_Toc191420901)

[7.1.3　プロトコール治療 19](#_Toc191420902)

[7.1.4　治療を変更した場合について 20](#_Toc191420903)

[7.1.5　周術期の管理 21](#_Toc191420904)

[7.1.6　サーベイランス 21](#_Toc191420905)

[7.1.7　後治療 21](#_Toc191420906)

[7.2　観察・検査項目およびスケジュール 22](#_Toc191420907)

[7.2.1　観察・検査スケジュール 22](#_Toc191420908)

[7.2.2　観察・検査項目 23](#_Toc191420909)

[7.3　併用薬（療法）に関する規定 24](#_Toc191420910)

[7.4　研究対象者への指導事項 24](#_Toc191420911)

[7.5　研究終了後の対応 25](#_Toc191420912)

[7.6　研究対象者ごとの研究中止基準 25](#_Toc191420913)

[８．評価に関する事項 26](#_Toc191420914)

[8.1　評価指標（エンドポイント） 26](#_Toc191420915)

[8.1.1　主要評価項目（Primary endpoint） 26](#_Toc191420916)

[8.1.2　副次評価項目（Secondary endpoint） 26](#_Toc191420917)

[8.2　評価指標に関する評価、記録 26](#_Toc191420918)

[8.3　評価指標に関する解析の方法ならびに時期 33](#_Toc191420919)

[９．有害事象・手術合併症・不具合の記録、報告等に関する事項 33](#_Toc191420920)

[9.1　有害事象・手術合併症・不具合に関する記録、報告、解析の方法 33](#_Toc191420921)

[9.2　予期される有害事象 35](#_Toc191420922)

[9.3　有害事象・手術合併症・不具合発生時の対応 36](#_Toc191420923)

[9.3.1　有害事象・手術合併症・不具合の発生時の対応 36](#_Toc191420924)

[9.3.2　重篤な有害事象・手術合併症・不具合の発生時の対応 37](#_Toc191420925)

[9.3.3　予測できない重篤な有害事象・手術合併症・不具合の発生時の対応 37](#_Toc191420926)

[9.4　有害事象・手術合併症・不具合発生後の研究対象者の観察 37](#_Toc191420927)

[10．統計的な解析に関する事項 37](#_Toc191420928)

[10.1　解析対象集団 37](#_Toc191420929)

[10.1.1　最大の解析対象集団 (Full Analysis Set、 FAS) 37](#_Toc191420930)

[10.1.2　安全性解析対象集団 (Safety Analysis Set、 SAS) 37](#_Toc191420931)

[10.2　目標登録数と設定根拠 38](#_Toc191420932)

[10.3　症例の取扱い 38](#_Toc191420933)

[10.4　データの取扱い 38](#_Toc191420934)

[10.5　統計解析項目および解析計画 38](#_Toc191420935)

[10.5.1　研究対象者の背景の要約 38](#_Toc191420936)

[10.5.2　主要評価項目の解析 39](#_Toc191420937)

[10.5.3　副次評価項目の解析 39](#_Toc191420938)

[10.5.4　サブグループ解析 41](#_Toc191420939)

[10.6　中間解析 41](#_Toc191420940)

[10.7　主たる解析 41](#_Toc191420941)

[10.8　最終解析 41](#_Toc191420942)

[11．原資料等の閲覧に関する事項 41](#_Toc191420943)

[12．品質管理および品質保証に関する事項 42](#_Toc191420944)

[12.1　モニタリング 42](#_Toc191420945)

[12.2　監査 42](#_Toc191420946)

[13．倫理的な配慮に関する事項 42](#_Toc191420947)

[13.1　法令等の遵守 42](#_Toc191420948)

[13.2　予期される利益、負担および不利益 43](#_Toc191420949)

[13.3　研究対象者に係わる遺伝的特徴等に関する研究結果や偶発的初見の取扱い 43](#_Toc191420950)

[14．情報・試料等の収集、保管、廃棄に関する事項 43](#_Toc191420951)

[14.1　情報の収集、保管、廃棄 43](#_Toc191420952)

[14.2　試料の収集、保管、廃棄 44](#_Toc191420953)

[15．金銭の支払いおよび補償に関する事項 44](#_Toc191420954)

[15.1　金銭の支払い（研究対象者の費用負担） 44](#_Toc191420955)

[15.2　補償に関する事項 44](#_Toc191420956)

[16．情報の公表 45](#_Toc191420957)

[17．実施期間 45](#_Toc191420958)

[18．研究対象者に対する説明および同意 45](#_Toc191420959)

[19．利益相反に関する事項 46](#_Toc191420960)

[20．知的財産権 46](#_Toc191420961)

[21．個人情報等の取扱い 46](#_Toc191420962)

[21.1　個人情報の保護 46](#_Toc191420963)

[21.2　データの二次利用 47](#_Toc191420964)

[22．研究計画書の遵守および研究計画書の変更 47](#_Toc191420965)

[22.1　研究計画書の遵守 47](#_Toc191420966)

[22.2　研究計画書の変更 47](#_Toc191420967)

[23．研究計画書からの逸脱（不適合）の取扱い 47](#_Toc191420968)

[24．研究機関の長への報告と方法 47](#_Toc191420969)

[25．研究の中止 48](#_Toc191420970)

[26．研究の終了 48](#_Toc191420971)

[27．参考資料・引用文献 48](#_Toc191420972)

**１．概要**

| **研究課題名** | 両開き式頚椎椎弓形成術におけるプレート法と縫合糸アンカー法の有用性に関する無作為化非劣性比較試験 |
| --- | --- |
| **研究の目的** | 頚椎椎弓形成手術を要する変性頚部脊髄症（DCM）患者を対象として、現在標準術式のひとつとなりつつあるプレートを用いた両開き式椎弓形成術（プレート法）と比較して、従来行われてきた安価な縫合糸アンカーを用いた手術（縫合糸アンカー法）の有効性・安全性が劣らないこと（非劣性）を、無作為化比較研究にて検証する。 |
| **研究デザイン** | 多施設共同、非盲検無作為化比較研究 |
| **研究の性質** | 検証的研究 |
| **研究対象者に対する治療** | ＜対照群＞  プレートを用いた両開き式椎弓形成術（プレート法）  （使用機器：LAMINAclip2、オリンパス・テルモバイオマテリアル株式会社）  ＜試験治療群＞  縫合糸アンカーを用いた両開き式椎弓形成術（縫合糸アンカー法）  （使用機器：LAMIFIX、オリンパス・テルモバイオマテリアル株式会社） |
| **対象** | 頚椎椎弓形成術の手術を要するDCM患者 |
| **選択基準** | 以下の基準のすべてに該当する患者を、本臨床研究に組み入れる。   1. DCM（頚椎症性脊髄症[CSM]あるいは頚椎後縦靭帯骨化症[OPLL]）による脊髄症状を有し、頚椎椎弓形成術を予定している患者 2. MRIまたはCTで脊髄の狭窄がC3-7レベルの患者 3. 同意取得時の年齢が20歳以上90歳未満の患者 4. 本臨床研究の参加に関して患者本人から文書で同意の得られた患者 |
| **除外基準** | 以下の基準のいずれかに該当する患者は、本臨床研究に組み入れない。   1. 術式として頚椎椎弓形成術が不適切である患者（MRIまたはCTで著明な頚椎後弯もしくは、脊髄の前方圧迫、頚椎局所不安定性を呈している患者等） 2. 椎間孔狭窄を合併している患者（後方椎間孔拡大術の併用を要する患者） 3. 脊椎感染症を有する患者 4. 脊椎腫瘍（転移性腫瘍を含む）を有する患者 5. 外傷性の圧迫性脊髄症の患者（中心性脊髄損傷の患者を含む） 6. 頚椎手術の既往を有する患者 7. 維持透析中の患者 8. 脳性麻痺を合併する患者 9. パーキンソン病の患者   10）妊婦または妊娠している可能性のある患者  11）その他、研究責任（分担）医師が本研究への参加が不適当であると判断した患者 |
| **評価項目** | **主要評価項目**  手術前後の頚椎JOAスコア改善率  頚椎JOAスコア改善率＝(術後スコア-術前スコア)/(17-術前スコア)×100(%)  主たる解析時点は手術後1年時とする。最終解析として手術後2年時の結果も示す。副次評価項目３）～13）についても同様。  ※JOA：日本整形外科学会  **副次評価項目**   1. 手術時間 2. 出血量 3. 術後1年・2年時における頚椎JOAスコア改善率の臨床的に意義のある最小変化量（MCID）達成割合 4. 術後1年・2年時における健康関連QOL（EQ-5D） 5. 術後1年・2年時における頚部痛、上肢痛、上肢しびれの程度（VAS） 6. 術後1年・2年時における手術前後の頚椎障害指数（NDI） 7. 術後1年時・2年時における拡大椎弓保持率（Retention rate） 8. 術後1年時・2年時におけるHinge骨折の割合 9. 術後１年時・2年時における骨癒合の割合 10. 術後１年・2年時における頚椎アライメント（C-SVA, C2-7角、T1 slope） 11. 術後1年時・2年時における傍脊柱筋断面積（C4/5レベル） 12. 術後１年・2年時における硬膜管面積(C3/4,C4/5,C5/6,C6/7レベル) 13. 術後１年・2年時における硬膜管後方圧迫分類 14. 増分費用効果比（ICER）   15）手術合併症発生割合 |
| **プロトコール治療** | 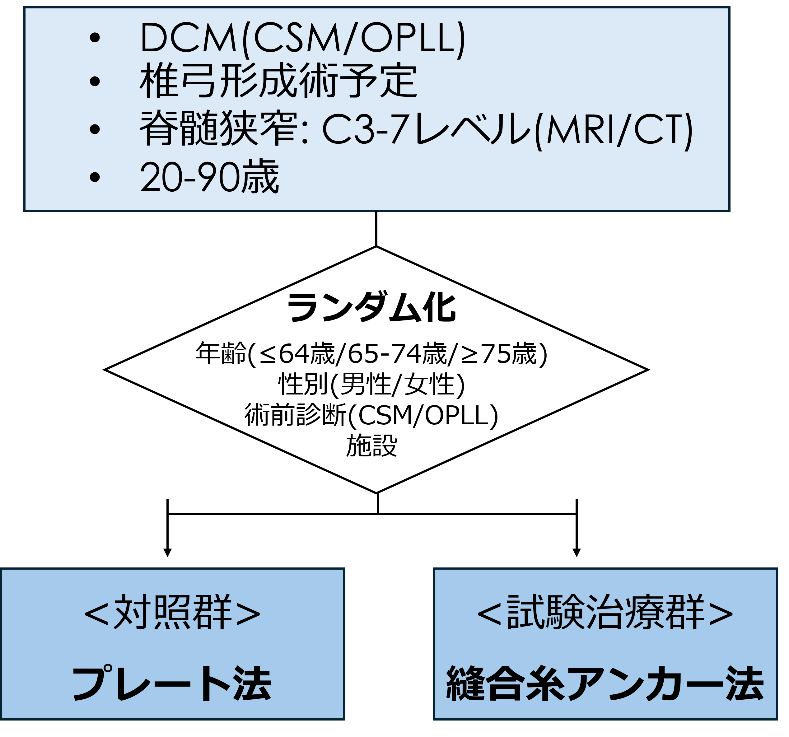  研究責任（分担）医師は、割付結果に従って手術（プレート法または縫合糸アンカー法）を行う。手術は頚椎椎弓形成術に精通した整形外科専門医が行う。手術方法、手術前後の評価スケジュールは以下の通りとする。  **【手術方法】**  全身麻酔、腹臥位でC2-7までの正中縦皮切を置く。椎間関節内側までの展開を行う。C3-6の椎弓に対して2-4椎弓を拡大する両開き式の椎弓形成を行う。  必要に応じてC2およびC7のドーム状椎弓切除を追加する。  ＜対照群＞プレート法  C3-6の拡大椎弓にスクリューを設置し、プレートを設置する。スクリュー・プレートのサイズは各症例の椎弓の形状に応じて決定する。  ＜試験治療群＞縫合糸アンカー法  C3-6のいずれかに外側塊にアンカーを設置し、黄色靱帯に縫合糸をかけて拡大位を保持するように縫縮する。  **【手術前後の評価スケジュール】**   1. 術前：X線、CT、MRI、臨床評価 2. 術後1週：CT 3. 術後1年：X線、CT、MRI、臨床評価、直接医療費 4. 術後2年：X線、CT、MRI、臨床評価、直接医療費 |
| **目標研究対象者数** | 216例 |
| **研究実施期間** | 総研究期間：2025年10月17日(jRCT公表日)～2031年3月31日（5.5年）  （予定登録期間：3年、追跡期間：登録終了後2年、解析期間：0.5年） |
| **研究施設数** | 3施設（東京科学大学病院、済生会川口総合病院、佐久医療センター） |

**２．臨床研究の実施体制**

| 役割 | 氏名 | 職名 | 所属機関・部署等※ | 連絡先 |
| --- | --- | --- | --- | --- |
| 研究代表者 | 吉井　俊貴 | 教授 | 東京科学大学病院  整形外科 | 03-5803-5279 |
| 調整管理実務担当者  （研究事務局） | 山田　賢太郎 | 助教 | 東京科学大学病院  整形外科 | 03-5803-5279 |
| データマネジメント責任者 | 坂巻　泰則 | URA | 東京科学大学病院 ヘルスサイエンスR&Dセンター | 03-5803-5465 |
| モニタリング責任者 | 桑本 幸接 | URA | 東京科学大学病院 ヘルスサイエンスR&Dセンター | 03-5803-5465 |
| 統計解析責任者 | 平川　晃弘 | 教授 | 東京科学大学 臨床統計学分野 | 03-5803-5150 |
| 統計解析担当者 | 佐藤　宏征 | 講師 | 東京科学大学 臨床統計学分野 | 03-5803-5150 |
|  | 花澤　遼一 | 特任助教 |  |  |
|  | 北林　遼 | 特任助教 |  |  |

＜研究実施医療機関＞

①　東京科学大学病院　整形外科

住所：〒113-8510　東京都文京区湯島1-5-45

電話番号：03-5803-5279

研究責任者：吉井 俊貴（教授）

②　済生会川口総合病院　整形外科

住所：〒332-8558　埼玉県川口市西川口5-11-5

電話番号：0570-08-1551

研究責任者：坂井 顕一郎（部長）

③　佐久医療センター　整形外科

住所：〒385-0051 長野県佐久市中込３４００−２８

電話番号：[0267-62-8181](https://www.google.com/search?q=%E4%BD%90%E4%B9%85%E5%8C%BB%E7%99%82%E3%82%BB%E3%83%B3%E3%82%BF%E3%83%BC&sca_esv=4518de3dcf4930bc&sxsrf=ADLYWIImcjXcC8O9TVzg3vByYSjiJuhtLw%3A1737425724450&source=hp&ei=PAOPZ-e_GO_b2roPk5TS8Q0&iflsig=AL9hbdgAAAAAZ48RTJbBZBIwAAg3MG4chxHkWT47jpL9&ved=0ahUKEwjnzYGZ34WLAxXvrVYBHROKNN4Q4dUDCBk&uact=5&oq=%E4%BD%90%E4%B9%85%E5%8C%BB%E7%99%82%E3%82%BB%E3%83%B3%E3%82%BF%E3%83%BC&gs_lp=Egdnd3Mtd2l6IhjkvZDkuYXljLvnmYLjgrvjg7Pjgr_jg7wyBRAAGIAEMgUQABiABDIFEAAYgAQyBRAAGIAEMgUQABiABDIFEAAYgAQyBRAAGIAEMgUQABiABDIFEAAYgAQyBRAAGIAESMMxUNwBWIAucAh4AJABAJgBwAGgAb0hqgEEMC4yNbgBA8gBAPgBAZgCHaAC4ByoAgvCAgYQswEYhQTCAgoQABgDGOoCGI8BwgIHEAAYgAQYBMICEBAAGIAEGLEDGIMBGAQYigXCAgsQABiABBixAxiDAcICDRAAGIAEGLEDGIMBGATCAgoQABiABBixAxgEwgIMEAAYgAQYBBhGGPkBwgIPEAAYgAQYsQMYBBhGGPkBwgIIEAAYgAQYsQPCAg4QABiABBixAxiDARiKBcICBhAAGAQYHpgDCfEFSys5ZuKbjT-SBwQ4LjIxoAeJZg&sclient=gws-wiz)

研究責任者：福島　和之 (部長)

**３．臨床研究の背景**

## **3.1 対象疾患について**


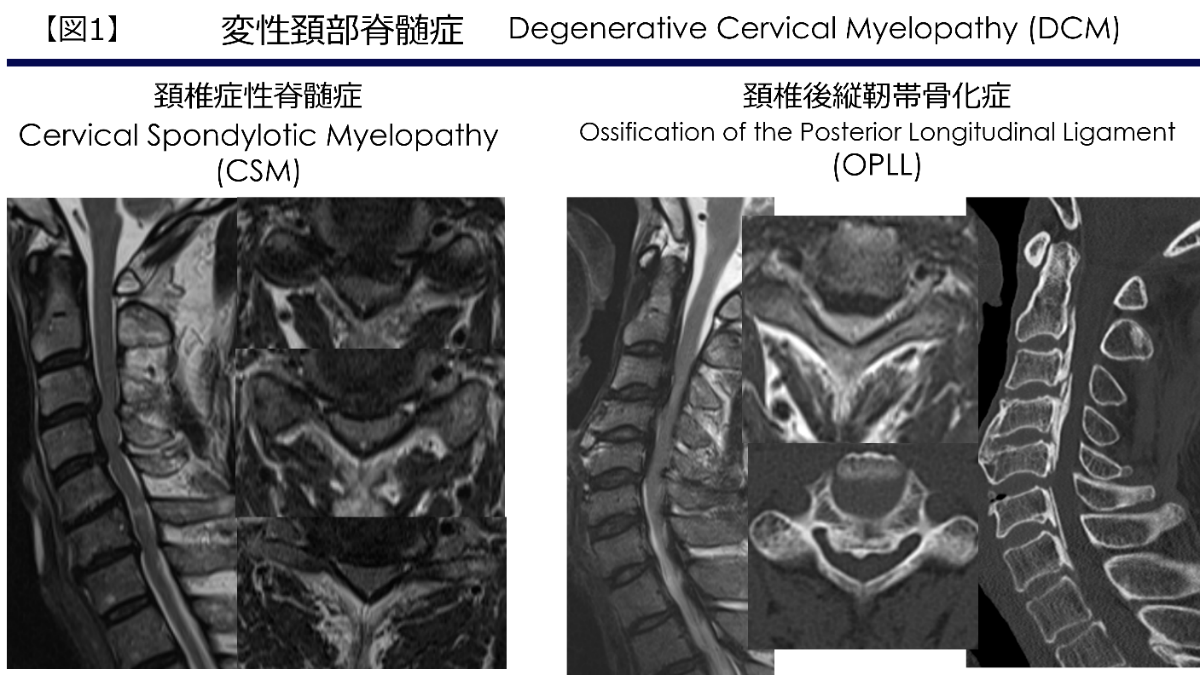
変性頚部脊髄症（degenerative spondylotic myelopathy: DCM）は、靭帯骨化を伴わない行性変化による脊髄の圧迫性病変を病態とする頚椎症性脊髄症（Cervical Spondylotic Myelopathy: CSM）と、頚椎後縦靭帯骨化症（Ossification of the posterior longitudinal ligament: OPLL）を含む疾患概念である（図1）。

DCMの有病率は人種間で差があると報告されており、米国では100万人あたり41-605人の有病率（0.004-0.06％）であると報告されている^1)^一方で、本邦では和歌山県の959人の住民健診によるとDCMの有病率は10.1%^2)^と報告されており、東洋人で有病率の高い疾患である。頚髄が圧迫されることにより、（知覚障害（上下肢のしびれ）の他に手指巧緻運動障害や痙性歩行をきたし、進行すると運動麻痺、感覚障害、膀胱直腸障害等をきたす。保存的治療の有効性は限定的であり、症状が進行した場合は手術療法が第一選択となる^3)^。

## **3.2 対象疾患に対する標準治療について**

DCMに対する手術療法は、上述のように東洋人で多い疾患であることから、本邦を中心に発展してきた。1970年代～80年代では広範椎弓切除（extensive laminectomy）が行われてきたが、術後の頚椎後弯進行あるいは椎弓切除後硬膜後方瘢痕による脊髄症状の再悪化等の問題が指摘され、1980年代後半からは、後方からアプローチする術式として頚椎椎弓形成術（Laminoplasty）が日本人医師により開発された。頚椎椎弓形成術は片開き式^4)^と両開き式^5)^の術式が提唱されている（図2）。


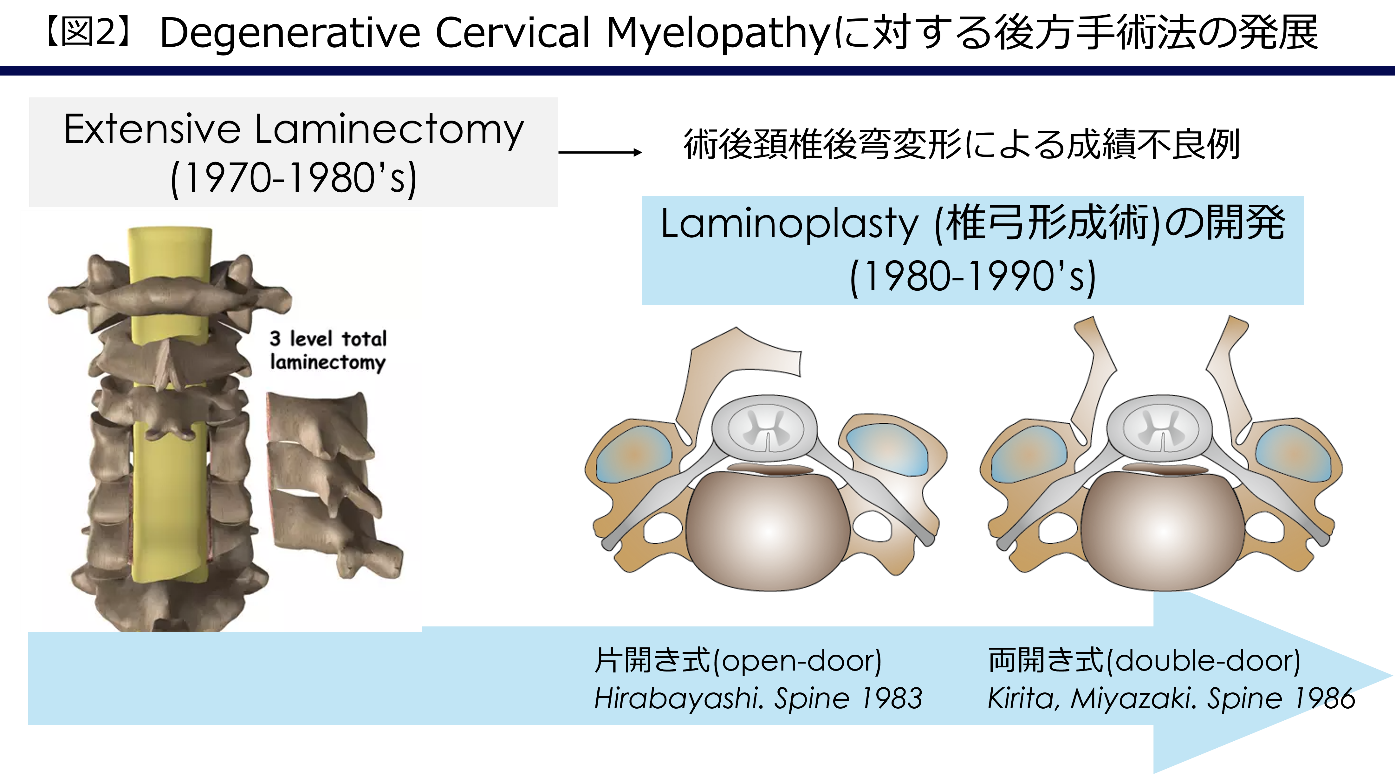


椎弓形成術は片開き式と両開き式の術式間で術後成績に差はないと報告されており、後弯変形または前方圧迫のないDCMの標準治療とされている^6)^。

両開き式椎弓形成術の問題点として、拡大した椎弓の再閉鎖（Lamina reclosure）による脊髄症の再悪化や頚椎後弯化が報告されている^7,8)^。そのため、拡大した椎弓の再閉鎖を防止するべく、縫合糸アンカー法（図３左）^9-11)^、椎弓スペーサー法（図３中）^12-15)^が開発され、近年ではより簡便なプレートを用いたプレート法（図3右）^16-18)^が普及している。


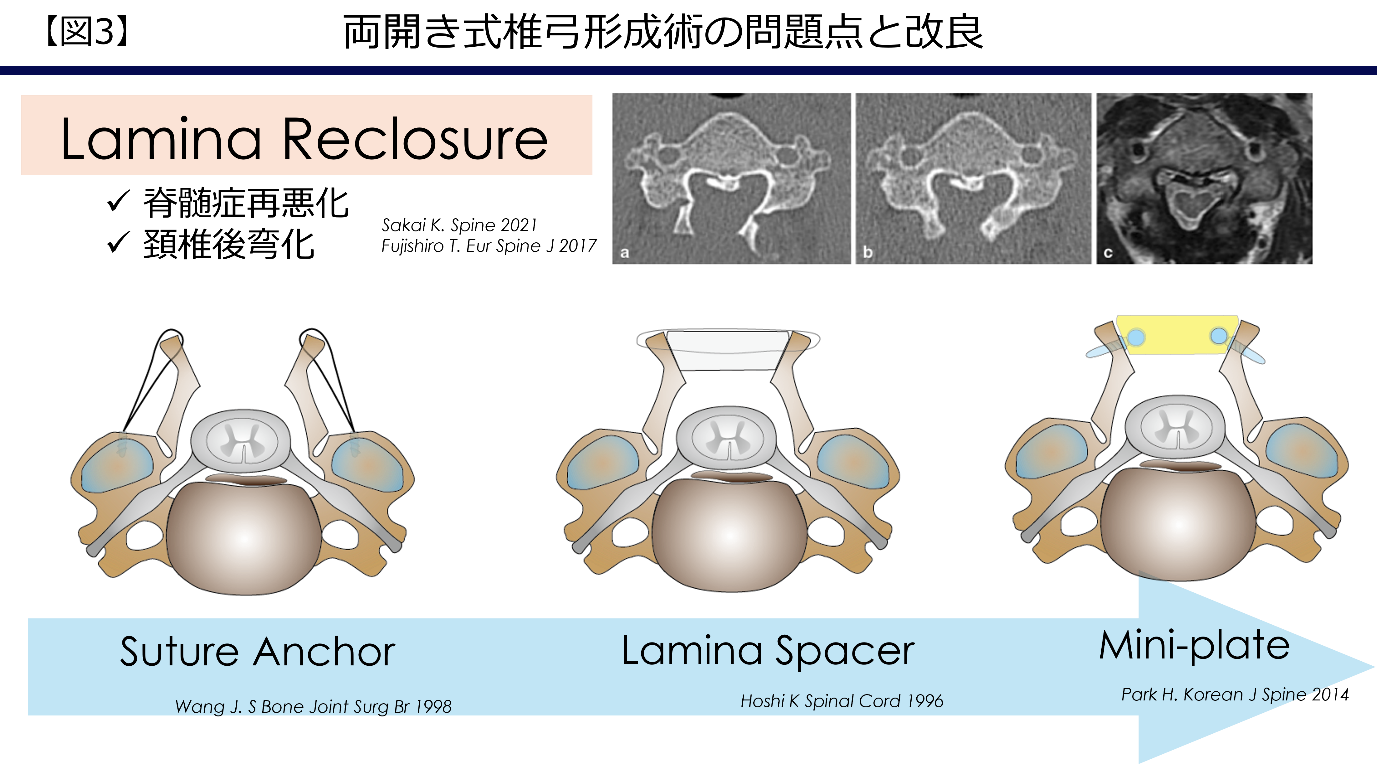


|  | 縫合糸アンカー法 | 椎弓スペーサー法 | プレート法 |
| --- | --- | --- | --- |
| 拡大した椎弓の再閉鎖率 | 1.8-5.5% | 2.9-21.3%  (スペーサー脱転率) | 3.4-13.3%  (スクリュー脱転率) |
| JOAスコア改善率 | 42.4-59.8% | 43.2-52.3％ | 47.1-83.4% |
| 手技の煩雑さ | 簡便 | 煩雑 | 簡便 |
| 費用（材料費） | 安い  138,000円／２椎弓 | 安い  120,800円／4椎弓 | 高い  506,700円／３椎弓 |
| 手術料（診療報酬） | 727,800円／5椎弓 | | |

　　　※JOAスコア：Japanese Orthopaedic Association（日本整形外科学会）スコア

縫合糸アンカー法でもJOAスコアの改善率は42.4-59.8%と良好な手術成績が報告されている^9-11)^一方で、プレート法でのより簡便な手術手順と優れた固定力の利点が報告されている^16-18)^。　しかしながらプレート法が手術成績において従来の縫合糸アンカー法と比べて優れているかについて良質な無作為化比較試験は行われておらず結論が出ていない。

またプレート法でもスクリュー脱転による拡大した椎弓の再閉鎖が3.4-13.3％で報告されており、縫合糸アンカー法の椎弓再閉鎖率（1.8-5.5%）よりもより安全かどうか、安全性を含んだ比較研究はこれまで報告はない。

３つの方法の手術料は同じであるが、材料費については、プレート法では本邦での保険償還価格では3椎弓に使用した場合506,700円となり、縫合糸アンカー法を2椎弓に使用した際の138,000円より高額である。これまで費用対効果に関する検討は行われておらず、高額な材料を用いてプレート法を行うメリットは明らかになっていない。

## **3.3 研究デザインの設定根拠**

そこで、本研究では、現在標準術式のひとつとなりつつあるプレート法と比較して、従来行われてきた安価な縫合糸アンカー法の有効性・安全性が劣らないこと（非劣性）を無作為化非劣性試験において示すことを目的とする。

主要評価項目は、当該手術で最も関心性のある有効性の指標として、JOAスコアを用いた。手術前後でのJOAスコア改善率^4)^は手術治療の治療効果の評価指標として一般的に用いられていることから、手術前後の頚椎JOAスコア改善率と設定した。頚椎JOAスコアは、圧迫性脊髄症の治療成績の評価として国際的にも最も使用されている指標である。

頚椎JOAスコア改善率＝(術後スコア-術前スコア)/(17-術前スコア)×100(%)

※JOA：日本整形外科学会

主たる解析時点は、術後1年以降の改善率は大きく変化しない^14)^との理由から、手術後1年時とした。最終解析として手術後2年時の結果も評価することとした。

## **3.4 本研究の意義**

　　頚椎椎弓形成術の術式・使用材料についての良質な無作為化比較試験はこれまで報告がない。現在標準治療となりつつあるプレート法に対して、従来の方法である縫合糸アンカー法が有効性および安全性において非劣性であり、かつ、安価であることを証明することが、本研究の期待される結果である。副次評価項目として費用対効果分析も行う。本研究結果により、高価な材料費を要するプレート法の必要性を議論するにあたり、重要なエビデンスを提示することとなり、今後の手術方法の選択に寄与すると考える。

# **４．臨床研究の目的**

頚椎椎弓形成手術を要する変性頚部脊髄症（DCM）患者を対象として、現在標準術式のひとつとなりつつあるプレートを用いた両開き式椎弓形成術（プレート法）と比較して、従来行われてきた安価な縫合糸アンカーを用いた手術（縫合糸アンカー法）の有効性・安全性が劣らないこと（非劣性）を、無作為化比較研究にて検証する。

**５．臨床研究の内容に関する事項**

**5.1　主要評価項目および副次評価項目**

**１）主要評価項目**

手術前後の頚椎JOAスコア改善率

【設定根拠】

頚椎JOAスコアは、DCMの治療成績の評価として国際的にも最も使用されている指標である。手術前後でのJOAスコア改善率^4)^は手術治療の治療効果の評価として一般的に用いられていることから、手術前後の頚椎JOAスコアの改善率を主要評価項目に設定した。主たる解析時点は、術後1年以降の改善率は大きく変化しない^14)^との理由から、手術後1年時とした。最終解析として手術後2年時の結果も評価することとした。

**２）副次評価項目**

1. 手術時間
2. 出血量
3. 術後1年・2年時における頚椎JOAスコア改善率の臨床的に意義のある最小変化量（MCID）達成割合
4. 術後1年・2年時における健康関連QOL（EQ-5D）
5. 術後1年・2年時における頚部痛、上肢痛、上肢しびれの程度（VAS）
6. 術後1年・2年時における手術前後の頚椎障害指数（NDI）
7. 術後1年時・2年時における拡大椎弓保持率（Retention rate）
8. 術後1年時・2年時におけるHinge骨折の割合
9. 術後１年時・2年時における骨癒合の割合
10. 術後１年・2年時における頚椎アライメント（C-SVA, C2-7角、C7 slope）
11. 術後1年時・2年時における傍脊柱筋断面積（C4/5レベル）
12. 術後１年・2年時における硬膜管面積(C3/4,C4/5,C5/6,C6/7レベル)
13. 術後１年・2年時における硬膜管後方圧迫分類
14. 増分費用効果比（ICER）

⑮　手術合併症発生割合

【設定根拠】

①・②　本研究の立案に至ったClinical Question（CQ）として、「プレート法は簡便な手術手技であるが、高額である。安価な従来法（縫合糸アンカー法）でもプレート法と比べて有効性・安全性は劣っていないのではないか？」が挙げられる。手術手技の簡便さは手術時間・出血量で測り知る事ができるため、CQに答える直接的な項目として副次的評価項目に設定した。

③　近年治療の有効性を評価する方法として、患者立脚型評価法の単純な比較のみならず、MCID（臨床的に意義のある最小変化量：Minimal clinically important difference）で評価することが一般的である。治療によりMCIDの閾値を超える変化があれば、その治療は効果があったとみなすことができる。圧迫性脊髄症におけるJOAスコア改善率のMCIDは52.8%と報告されている^19)^。改善率の直接群間比較のみならずMCID達成割合についても、副次評価項目として治療群間で１年時および２年時の比較を行う。

④～⑥　JOAスコアは頚椎に由来する脊髄症状の医師主導評価である。患者立脚型アウトカムであるEQ-5DやVAS、NDIを副次評価項目に設定した。なお、EQ-5Dは⑪増分費用効果比を算出する際の効用値としても使用する。

⑦　安全性に関する評価項目として、拡大椎弓再閉鎖に対する客観的指標であるRetention rate^7)^(図4)を副次評価項目に設定した。

⑧　安全性に関する評価項目として、CTにおける拡大椎弓のHinge骨折の割合^8）^を副次評価項目に設定した。

⑨　手術目的達成に対する評価項目として、CTでHinge骨折の骨癒合の割合を副次評価項目に設定した。


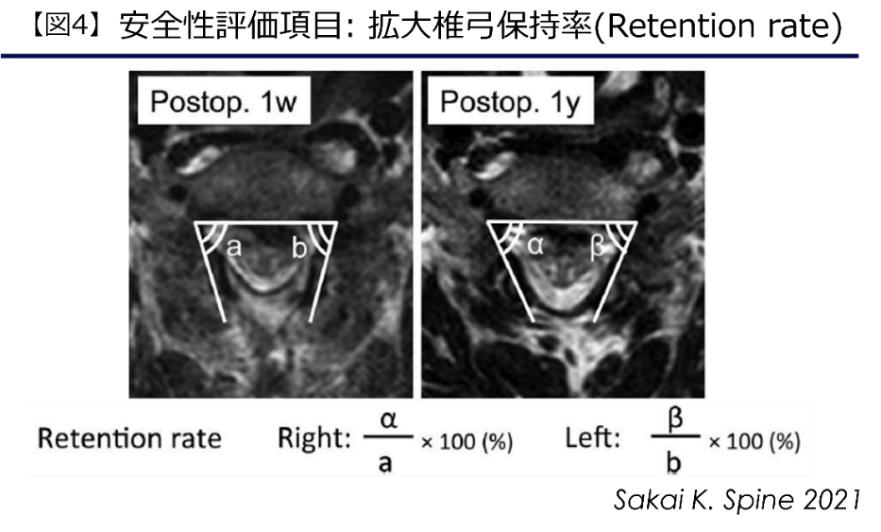


⑩　術後の頚椎の後弯変形は脊髄症状の再悪化につながり、頚椎椎弓形成術の成績不良を惹起する形態的変化である^7-9)^。後弯変形の指標としてC2-7前弯角およびC-SVA、またこれらに影響する因子としてC7 slopeを副次評価項目に設定した（図5）。

⑪　頚椎伸展筋である傍脊柱筋の断面積（図6）の減少は、項部痛に影響する因子^20)^、また椎弓形成術後の椎弓再閉鎖に関わる因子^7)^として報告されているため、術後のC4-5レベルにおける傍脊柱筋面積を副次評価項目に設定した。


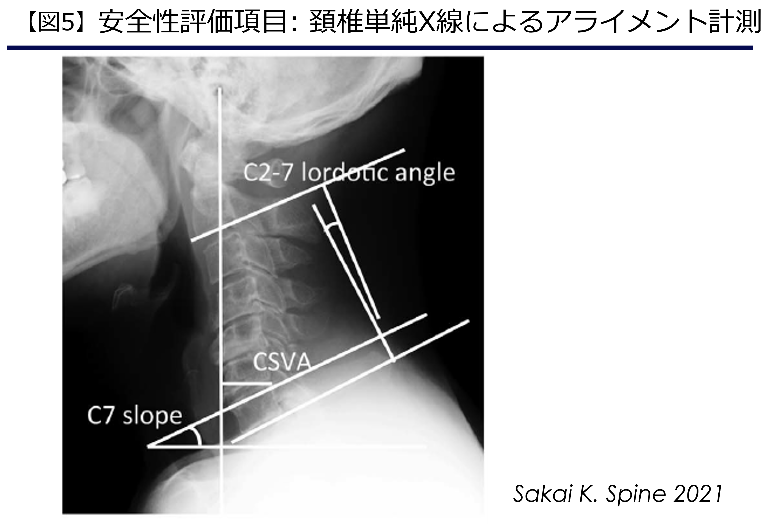

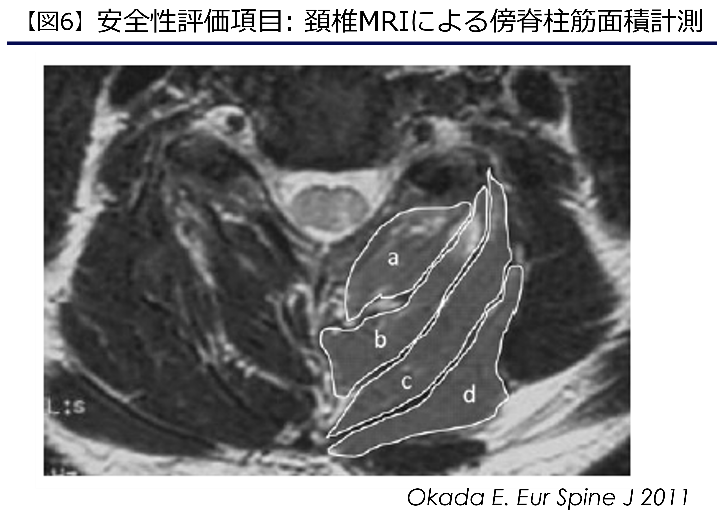


⑫⑬　椎弓切除後の硬膜後方瘢痕による脊髄の再圧迫はpost-laminectomy membraneと呼ばれ、脊髄症再燃の要因と推察されている^21)^が、椎弓形成の各手術方法における発生頻度は明らかになっていない。術後の硬膜管面積と硬膜管後方圧迫分類^22)^（図7）を脊髄の再圧迫の評価として副次評価項目に設定した。

⑭　本研究では、試験治療（縫合糸アンカー法）が対照治療（プレート法）に比べて、有効性・安全性は劣らず、より安価であり、費用対効果に優れていることを示すことが重要な目的の１つである。試験治療群と対照群の費用対効果を比較する指標として、一般的な指標であるICERを副次評価項目に設定した。

⑮　再手術を含む手術合併症の発生頻度は手術治療の臨床的有用性を評価する指標として一般的な指標であることから、副次評価項目に設定した。


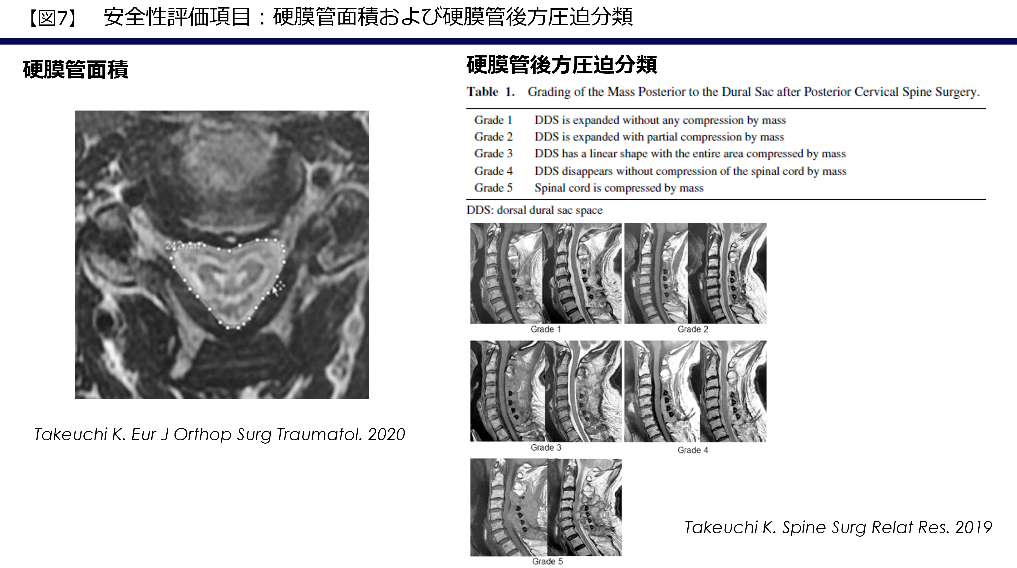


## **5.2　臨床研究のデザインおよびアウトライン**

**5.2.1　臨床研究のデザイン**

１）研究の性質：検証的研究

２）無作為化：無作為化比較

３）盲検化：非盲検

４）対照：実薬（治療）対照

５）割付：並行群間比較


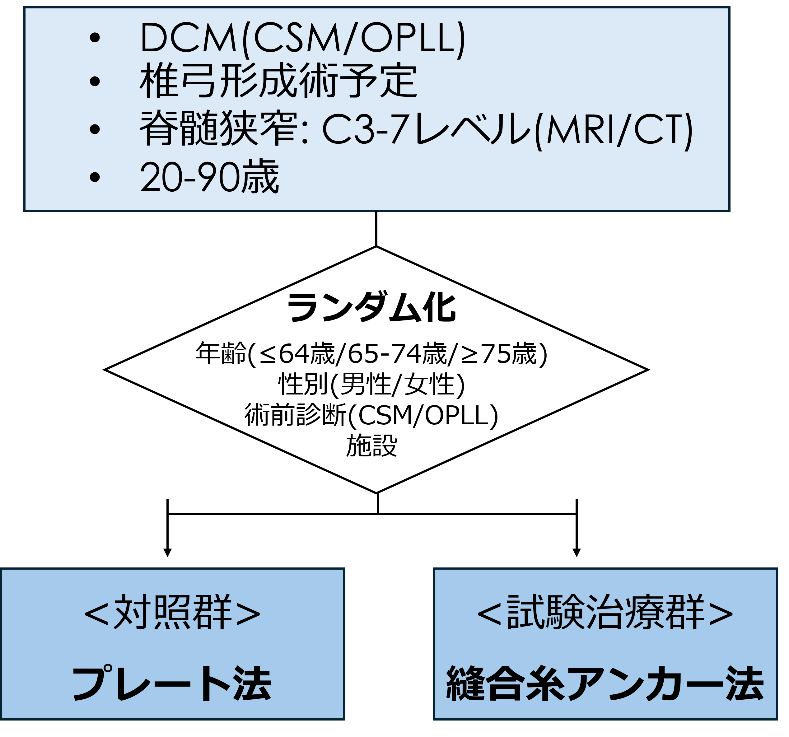
**5.2.2　臨床研究のアウトライン**

**5.3　症例登録・割付方法**

１）症例登録方法

研究責任（分担）医師は、研究対象者として文書同意を取得した患者が全ての選択基準を満たし、全ての除外基準に抵触しないことを確認する。その後、本臨床研究に従事する者は、EDCシステムに患者情報を入力し、研究対象者の登録および割付を行う。

研究対象者の登録番号および拡大椎弓保持の方法（プレート法または縫合アンカー法）は、EDCシステムに表示されると共に、印刷・ダウンロード可能なPDFファイルにも記録される。研究に従事する者は、研究対象者の登録番号および割り付けられた拡大椎弓保持の方法（プレート法または縫合アンカー法）を確認し、研究対象者名簿などに記録または割付結果の記載されたPDFを印刷・保管する。

２）割付方法

- 割付方法：最小化法
- 割付調整因子：①施設、②年齢、③性別、④術前診断

【設定根拠】

1. 施設（東京科学大学病院／済生会川口総合病院/佐久医療センター）

登録患者の背景、短期成績等の施設間差が存在する可能性があるため。

1. 年齢（64歳以下／65～74歳／75歳以上）
2. 性別（男性／女性）

年齢、性別は、DCM患者の治療成績における一般的な予後因子であるため。

④　術前診断（頚椎症性脊髄症（CSM）/後縦靭帯骨化症（OPLL））

病態によって治療予後が異なることが報告されているため。

**5.4　研究対象者の参加予定期間**

参加予定期間：約２年

（登録～手術までの期間：最長90日、追跡期間：手術後2年）

参加予定期間終了後、研究の実施に起因すると疑われる有害事象等が認められた場合、研究対象者の安全が確保されたと研究責任（分担）医師が判断するまでフォローアップを行う。なお、当該フォローアップ期間に関しては、研究期間には含まないものとする。

**5.5　臨床研究全体の中止基準**

研究代表者は、次の事例があった場合、研究実施継続の可否を検討する。

1. 研究の実施または継続に影響を及ぼすような重要な情報、その他研究を適正に行うために重要な情報を知った場合。
2. 研究対象者の組入れが困難で、予定症例数を達成することが困難であると判断されたとき。
3. 予定症例数または予定期間終了に達する前に、中間解析等により研究の目的が達成されたとき。
4. 倫理審査委員会等の意見として研究計画書等に対する修正の指示があり、これを受け入れることが困難な場合。
5. 倫理審査委員会等が中止の判断をした場合。
6. 臨床研究法、施行規則または本研究計画書に重大なまたは継続的な違反が生じた場合。

**６．研究対象者の選択および除外関する基準**

**6.1　研究対象者（対象疾患）**

頚椎椎弓形成術の手術を要する変性頚部脊髄症（DCM）患者

**6.2　選択基準**

以下の基準のすべてに該当する患者を、本臨床研究に組み入れる。

1)　DCM（頚椎症性脊髄症[CSM]あるいは頚椎後縦靭帯骨化症[OPLL]）による脊髄症状を有し、椎弓形成術を予定している患者

2)　MRIまたはCTで脊髄の狭窄がC3-7レベルの患者

3)　同意取得時の年齢が20歳以上90歳未満の患者

4)　本臨床研究の参加に関して患者本人から文書で同意の得られた患者

【設定根拠】

1. 研究対象者の病態学的な特徴を統一する観点から設定した。
2. DCMに対する椎弓形成術を施行する椎間高位範囲として、対照治療であるプレート法と試験治療である縫合糸アンカー法を比較する本研究の目的に合致する対象を選択するために設定した。
3. 個人の同意が成立する年齢および能力を考慮して、20歳以上と設定した。また、研究治療を安全に行える対象を選択する観点から、90歳以下と設定した。

4）担当医師が、他の全般的要因も勘案して判断するために設定した。

## **6.3　除外基準**

以下の基準のいずれかに該当する患者は、本臨床研究に組み入れない。

1) 術式として頚椎椎弓形成術が不適切である患者（MRIまたはCTで著明な頚椎後弯もしくは、脊髄の前方圧迫、頚椎局所不安定性を呈している患者等）

2) 椎間孔狭窄を合併している患者（後方椎間孔拡大術の併用を要する患者）

3) 脊椎感染症を有する患者

4）　脊椎腫瘍（転移性腫瘍を含む）を有する患者

5）　外傷性の圧迫性脊髄症の患者（中心性脊髄損傷の患者を含む）

6）　頚椎手術の既往を有する患者

7）　維持透析中の患者

8）　脳性麻痺を合併する患者

9）　パーキンソン病の患者

10) 妊婦または妊娠している可能性のある患者

11) その他、研究責任（分担）医師が本研究への参加が不適当であると判断した患者

【設定根拠】

1. 主要評価項目であるJOAスコアを始めとした治療予後に影響する可能性があることから設定した。
2. 研究対象者の術前の状態（脊髄症の症状）を統一する観点から設定した

3～9）主要評価項目であるJOAスコアを始めとした予後に影響する可能性があることから設定した。

10）安全性および倫理的配慮の観点から設定した。

11）研究責任（分担）医師が、他の全般的要因も勘案して判断するために設定した。

# **７．研究対象者に対する治療に関する事項**

## **7.1　プロトコール治療の実施手順**

**7.1.1　同意取得**

- 担当医師は、対象患者の診断およびその他の適格基準を確認した上で、文書にて本研究に関する説明を行い、本研究に対する理解を得たうえで、本人から文書同意を取得する。
- 適格性判定のための検査については、登録前360日以内に実施している場合は、同意取得前のデータであっても当該データを使用できるものとする。

**7.1.2　登録・割付**

- 担当医師は、文書同意を取得した後、研究対象者の適格性を再度確認した上で、当該研究対象者を本研究EDCシステムに登録する。登録された研究対象者は、EDCシステムにより、プレート法群または縫合糸アンカー法群に無作為に割付けられる。

※必ず手術前日までに登録・割付を完了させること。

**7.1.3　プロトコール治療**

- 登録後90日以内にプロトコール治療（頚椎椎弓形成術）を実施する。
- なんらかの理由で90日以内にプロトコール治療を実施できなかった場合は、「プロトコール治療中止」として、中止の理由等の情報を症例報告書に入力する。
- 登録後、プロトコール治療実施日（手術日）までに原疾患や併存疾患が悪化するなどして適格基準を満たさなくなった場合に、研究参加を継続しプロトコール治療として手術を実施するか否かは、担当医師の判断に一任する。症例報告書には、登録～治療実施日までの間に適格基準を満たさなくなった旨およびその詳細について記録を残す。
- DCMに対する椎弓形成術を、プレート法もしくは縫合糸アンカー法で行う。
- 全身麻酔で手術は行う。
- 腹臥位でC2-7までの正中縦皮切を置く。椎間関節内側までの展開を行う。
- C3-6の椎弓に対して2-4椎弓を拡大する両開き式の椎弓形成を行う。

椎弓を拡大する棘突起の先端を切除、椎弓正中部をハイスピードドリルを用い正中縦割する。椎間関節内側部でハイスピードドリルを用いて椎弓が拡大できる適度な硬さとなるように溝を掘削する。拡大予定の椎弓すべてで正中縦割・溝を作成した後に粘膜剥離子を用いて徐々に椎弓を拡大する。浮上した黄色靭帯を正中で切離し、硬膜との癒着を剥離、硬膜の膨隆をもって除圧の確認を行う。適宜硬膜外静脈叢からの出血を止血する。

- 担当医師は、登録時の割付結果に従い、プレート法、縫合糸アンカー法のいずれかの方法で拡大椎弓保持法を行う。
- 椎弓拡大の頭尾側の椎弓にドーム状椎弓切除を追加してもよい。
- 閉創前に椎弓後面にドレーンを留置する。ドレーンの規格・留置期間については規定しない。
- いかなる併施術式であっても手術時間、出血量、術中・術後合併症などはDCMの手術に含める。詳細を診療録（もしくは手術記録）と症例報告書に記載する。

⇒拡大した椎弓の保持法以外については、手術チームが最善と考える設定・方法にて手術を行う（7.1.4参照）。

- 各実施医療機関に日本脊椎脊髄病学会　脊椎脊髄外科指導医が勤務していることを、研究参加の条件とする。
- 担当医師は、可能な限り割り付けられた拡大椎弓保持法（プレート法／縫合糸アンカー法）にて手術を行う。もし、割り付けられた群とは異なる拡大椎弓保持法を行う場合には、その理由を症例報告書に入力する。（7.1.4参照）

　　プレート法・縫合糸アンカー法の手技の概要は以下の通り。

**＜対照群＞プレート法**

椎弓を拡大後に、拡大した椎弓の両側にLAMINAclip2（オリンパス・テルモバイオマテリアル株式会社）のスクリューを設置するための骨孔を作成し、スクリューを設置する。

スクリューヘッドに専用の機具を用いてプレート：LAMINAclip2（オリンパス・テルモバイオマテリアル株式会社）をかぶせて設置する。

スクリュー・プレートのサイズは各症例の椎弓の形状に応じて決定する。

使用するプレート数は2つ以上と規定する。


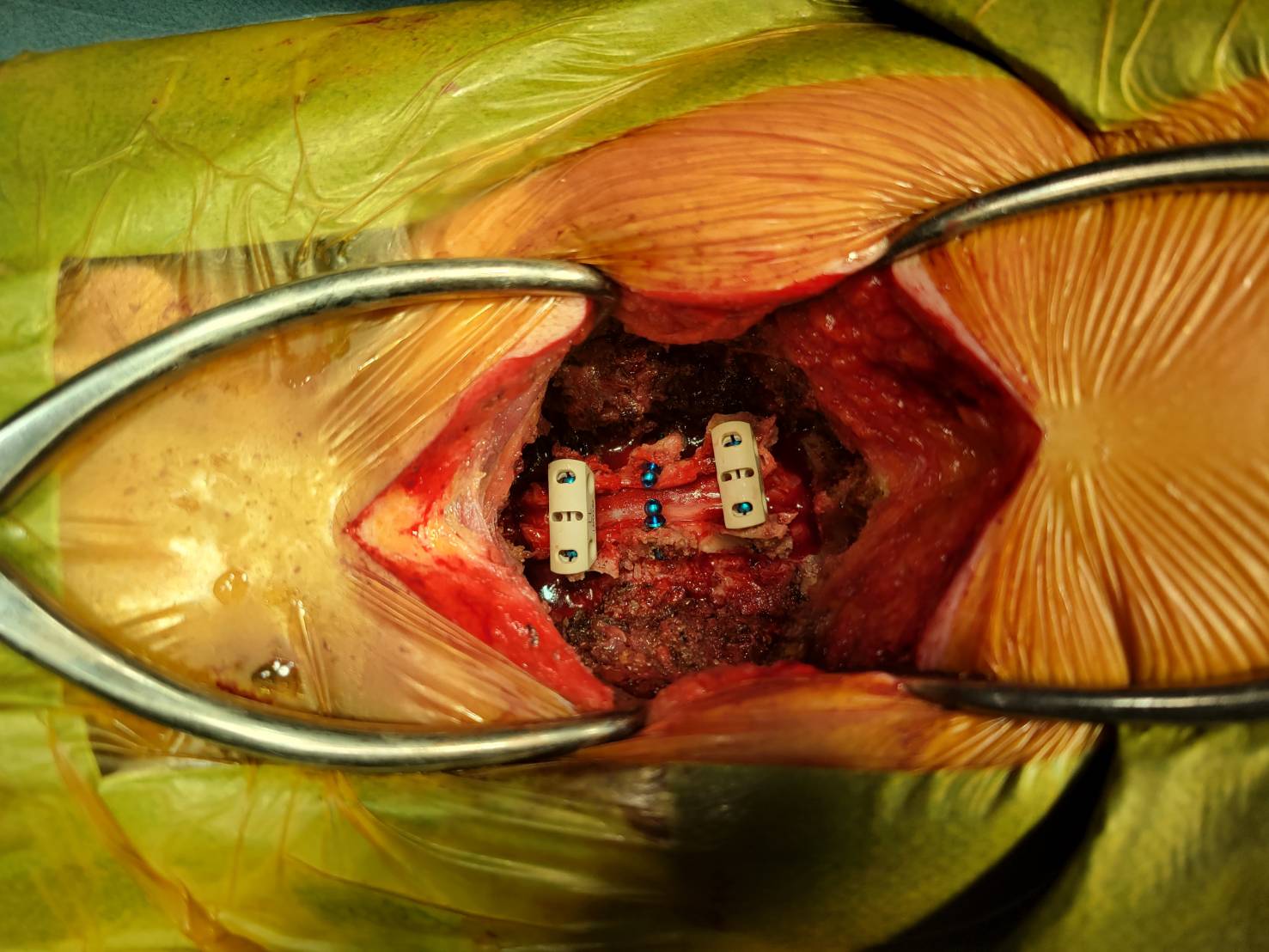

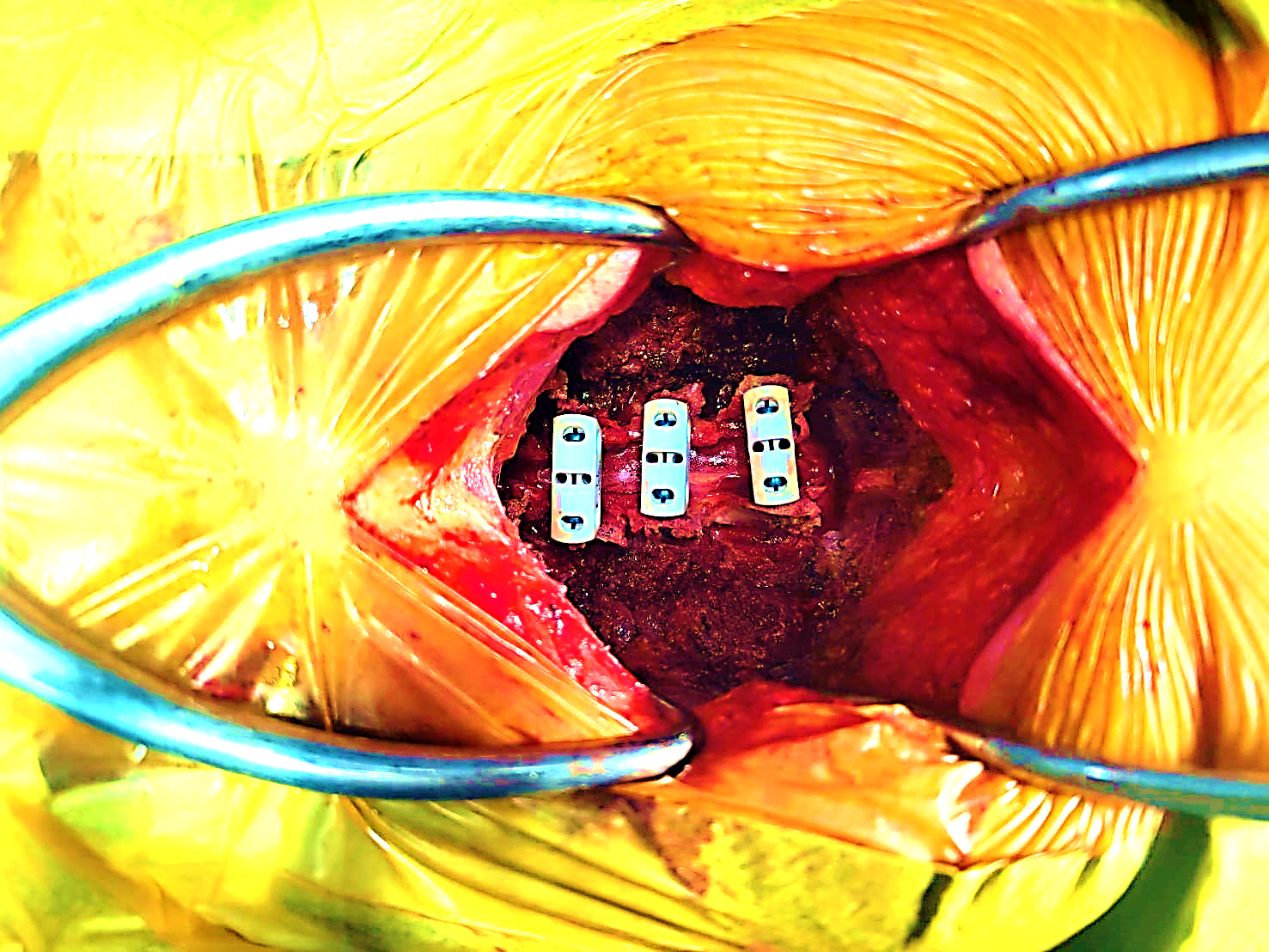

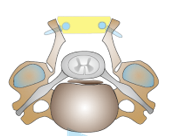


**＜試験治療群＞縫合糸アンカー法**


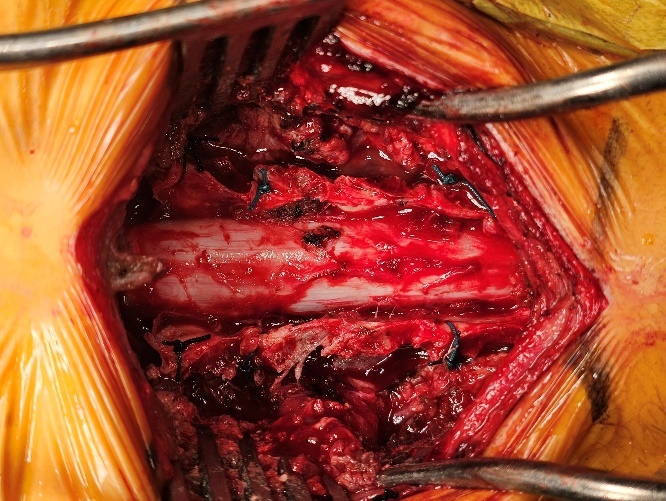

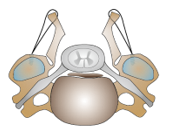


椎弓を拡大後に、拡大した椎弓の両側の外側塊にLAMIFIX（オリンパス・テルモバイオマテリアル株式会社）のアンカーを設置する。アンカーに連結された縫合糸を拡大椎弓間の切離した黄色靭帯に縫着することで拡大椎弓の保持を行う。

使用する縫合糸アンカー数は2つ以上と規定する。

**7.1.4　治療を変更した場合について**

＜割り付けられた拡大椎弓保持法ができなかった場合＞

登録時に割り付けられた拡大椎弓保持法（プレート法または縫合糸アンカー法）が、なんらかの理由で実施不可能だった場合は、担当医師が最善と考える方法により硬膜管の除圧を行う。研究対象者の安全や脊髄症の改善を最優先とし、適切に判断すること。

割り付けられた拡大椎弓保持法が実施不可能だった理由、実施した拡大椎弓保持法等について、症例報告書に入力する。

＜後方または前方固定術に移行した場合＞

手術中に担当医師が、頚椎に著しい不安定性があると判断した場合、もしくは術中合併症（椎弓骨折、椎体骨折など）の対処のために必要と判断した場合には、椎弓形成術から頚椎後方固定術または前方固定術に移行する。研究対象者の安全や脊髄症の改善、脊椎安定性を最優先とし、適切に判断すること。

- 椎弓形成が不可能だったため頚椎固定術に移行した場合、もしくは椎弓形成を行う前に頚椎固定手術に移行した場合は、理由等について症例報告書に入力する。
- 当該患者においては研究中止とし（7.6参照）、手術チームが最善と考える設定・方法にてDCMに対する手術を行うこと。

**7.1.5　周術期の管理**

周術期管理とは、予防的抗菌薬の種類と投与期間、術前・術後の輸液管理、術後の経口摂取開始時期や食事の内容、創およびドレーン管理、術後頚椎外固定等を含む。周術期の鎮痛剤の使用方法に関しても各実施医療機関で通常行っている方法に従う。

術後頚椎外固定の有無・種類は問わないが、術後頚椎外固定の内容は症例報告書に記入する。

術後離床時期、リハビリ内容は問わないが、リハビリはすべての患者で実施するものする。

**7.1.6　サーベイランス**

「7.2 観察・検査項目およびスケジュール」に従い、術後調査を行う。

手術合併症、その他の有害事象等が確認された場合は、7.2.2に定める項目のデータを収集し、EDCシステムに入力する。

**7.1.7　後治療**

割り付けられた拡大椎弓保持法吻合法ができなかった場合、以降の治療については規定しない。ただし、行った治療の内容については症例報告書に記録することとする。

**7.2　観察・検査項目およびスケジュール**

**7.2.1　観察・検査スケジュール**

本研究における調査、観察および評価項目ならびに来院スケジュールは以下の通りとする。

|  | 登録前  ～登録 | 手術日 | 術後  1週 | 退院日 | 術後  30日 | 術後  1年 | 術後  2年 |  |
| --- | --- | --- | --- | --- | --- | --- | --- | --- |
| 許容される期間 |  |  |  |  | ±14日  以内 | ±56日  以内 | ±56日  以内 | |
| 登録・割付 | ● |  |  |  |  |  |  |  |
| 患者背景情報 | ●※1 |  |  |  |  |  |  |  |
| 手術（プロトコール治療） |  | ● |  |  |  |  |  | |
| 手術情報 |  | ● |  |  |  |  |  |  |
| 術中合併症／有害事象 |  | ● |  |  |  |  |  |  |
| 術後早期合併症／有害事象 |  |  |  |  |  |  |  |  |
| 問診・診察 | ●※1 |  | ● | ● | ● | ● | ● |  |
| JOAスコア | ●※1 |  |  |  |  | ● | ● |  |
| 頚椎単純X線  （頚椎アライメント） | ●※1 |  | ● |  |  | ● | ● |  |
| 頚椎単純CT  （術後：拡大椎弓保持率、Hinge骨折・骨癒合） | ●※1 |  | ● |  |  | ● | ● |  |
| 頚椎単純MRI  （傍脊柱筋断面積） | ●※1 |  |  |  |  | ● | ● |  |
| 頚椎単純MRI  （硬膜管面積） | ●※1 |  |  |  |  | ● | ● |  |
| 頚椎単純MRI  （硬膜後方圧迫分類） |  |  |  |  |  | ● | ● |  |
| 術後晩期合併症／有害事象 |  |  |  |  |  |  |  |  |
| 健康関連QoL  （EQ-5D） | ●※1 |  |  |  |  | ● | ● |  |
| 疼痛・しびれの程度（VAS） | ●※1 |  |  |  |  | ● | ● |  |
| 頚椎障害指数（NDI） | ●※1 |  |  |  |  | ● | ● |  |
| 転帰調査 |  |  |  |  |  | ● | ● |  |
| 直接医療費 |  |  |  |  |  |  |  |  |

● ：必須

※1 ：本臨床研究に関する全ての調査・観察・検査は、研究対象者から文書による同意を得た後に実施する。ただし、同意取得前に通常診療として実施した調査・観察・検査等の結果は、登録前360日以内であれば研究データとして利用可能とする。

**7.2.2　観察・検査項目**

本研究における観察・検査項目は以下の通りである。実施時期については、「7.2.1　スケジュール」に従う。

| 実施項目 | | 観察・検査項目 | |
| --- | --- | --- | --- |
| 同意取得・登録・割付 | | 文書による同意取得日 | |
| 患者背景情報 | 一般的所見 | 性別、生年月日、身体所見（身長、体重、BMI、）、喫煙歴、  併存疾患、SA（American Society of Anesthesiologists）分類、内服薬（抗血栓薬、ステロイド等）職業、罹病期間、頚椎手術の既往 | |
|  | DCMの臨床所見/画像所見 | 術前診断 | CSM/OPLL |
|  |  | JOAスコア | 17点満点 |
|  |  | 健康関連QOL（EQ-5D） | 0-1 |
|  |  | 頚部痛、上肢痛、上肢しびれの程度（VAS） | 0-100mm |
|  |  | NDI | 50点満点 |
|  |  | 単純X線 | 頚椎可動域、矢状面バランス(C2-7角、C-SVA、C7 slope)、  OPLLの場合、：骨化巣形態(分節型/連続型/混合型)、高位ならびに最大骨化占拠率 |
|  |  | MRI | 最大狭窄レベル、T2矢状断髄内信号変化レベル、C4/5傍脊柱筋断面積(mm^2^) |
| 手術情報 | | 手術日、脊椎脊髄指導医の参加（なし／術者として参加／助手として参加）、術式（拡大椎弓高位、ドーム状椎弓切除の有無）、手術時間、出血量、拡大椎弓保持法（プレート法／縫合糸アンカー法）、使用機器（プレート/縫合糸アンカーの種類）、使用したインプラントのサイズ（長さ、大きさ）、輸血量（術中及び術後3日まで）、使用した止血製剤の種類、ドレーンの有無、頚椎固定術移行の有無（移行なし／頚椎後方固定移行/頚椎前方固定移行） | |
| 術中合併症/有害事象 | | 発生した場合は、合併症名、転帰、合併症への対応、頚椎手術との関連性、治療内容・治療実施日、入院期間延長の有無などを記載する。 | |
| 術後経過 | | 術後外固定の種類・有無・期間  入院日・退院日→術後在院日数  退院時の転帰  入院中の再手術の有無 | |
| 術後早期合併症／有害事象（術後30日以内） | | 問診、診察等により、有害事象の有無を確認する。  有害事象が発生した場合は、事象名、発現時期、転帰、重篤性（再手術の有無）、頚椎手術との関連性、治療内容・治療実施日、入院期間延長の有無などを記載する。 | |
| 問診・診察 | | 術後定期の外来にて、問診・診察を行う。 | |
| 単純X線 | | 頚椎(正面/側面/前屈側面/後屈側面)：C2-7角/C-SVA/C7 slopeを確認する  全脊柱立位(正面/側面): SVA/腰椎前弯角/胸椎後弯角/Pelvic incidence、既存椎体骨折を確認する | |
| 頚椎単純CT | | 各椎間スライスに合わせた水平断像、矢状断像、冠状断像：OPLLの有無/Lamina retention rate、Hingeの骨折/骨癒合を確認する。 | |
| 頚椎単純MRI | | T2およびT1矢状断像/水平断像  術前：最大狭窄レベル、髄内信号変化の有無、C4/5傍脊柱筋面積、硬膜管面積  術後： C4/5傍脊柱筋面積、硬膜管面積、硬膜管後方圧迫分類 | |
| 術後晩期合併症／有害事象（術後31日から術後2年以内） | | 問診、診察等により、有害事象の有無を確認する。  有害事象が発生した場合は、事象名、発現時期、程度、転帰、重篤性（再手術の有無）、頚椎手術との関連性、治療内容・治療実施日、入院の有無などを記載する。 | |
| 転帰調査 | | 生存（最終生存確認日、死亡の有無、死亡日、死亡理由）  再手術（再手術の有無、再手術日、再手術の理由、再手術方法）  研究中止（中止の有無、中止日、中止理由） | |
| 直接医療費 | | 入院費用（手術を除く診療報酬，手術関連費用[手術，手術薬剤，麻酔，麻酔薬剤]）  外来費用（診療，検査，薬剤，その他）  有害事象に対する入院再治療費用（手術を除く入院時診療報酬，手術関連費用[手術，手術薬剤，麻酔，麻酔薬剤]）  上記の費用をレセプトから研究分担者が収集する。 | |

**7.3　併用薬（療法）に関する規定**

**7.3.1　併用制限薬（療法）**

特に無し

**7.3.2　併用禁止薬（療法）**

特に無し

**7.4　研究対象者への指導事項**

研究責任（分担）医師は、研究開始前に研究対象者に対して以下の指導を行う。

1. 他科・他院を受診する際は、必ず研究に参加していることを当該医師または薬剤師に告げるとともに、可能な限り事前に担当医師に相談するよう指導する。

なお、事前に相談ができなかった場合は、事後に必ず担当医師に報告するよう指導する。

1. 体調不良が認められた場合は速やかに担当医師に報告し、受診の必要性について相談するよう指導する。
2. 本臨床研究に関する情報を、SNS等に公開しないよう、指導する。

**7.5　研究終了後の対応**

研究対象者が、研究参加終了後においても研究の結果により得られた最善の診断および治療を受けることができるように努める。

**7.6　研究対象者ごとの研究中止基準**

以下の中止基準に該当する場合は、研究を中止する。

1. 登録後９０日以内にプロトコール治療（椎弓形成術）が実施できなかった場合
2. 研究対象者の自由意思による同意撤回の申し入れがあった場合
3. 登録後に、画像診断もしくは臨床診断により椎弓形成手術が適応ではないと判断された場合
4. 術中所見にて椎弓形成が不可能もしくは椎弓形成前に頚椎固定術移行した場合
5. 術中の合併症（椎弓骨折、椎体骨折）で術中または術後に固定が必要となった場合
6. 臨床研究法および施行規則違反、選択基準違反または除外基準違反など、研究計画書からの重大な逸脱が判明した場合
7. 研究計画書の遵守が不可能になった場合
8. 研究対象者の妊娠が判明した場合
9. 研究全体が中止された場合
10. 研究対象者が死亡した場合
11. その他、研究責任者または担当医師により、研究の継続が困難と判断された場合

※プロトコール治療中止日は、1～3）・5）～9）・11）の場合はプロトコール治療中止と判断した日、4）の場合は手術日、 10）の場合は死亡日とする。

【設定根拠】

研究を倫理的に実施するため、また、研究対象者の安全性に配慮して設定した。

【中止時の対応方法】

研究責任医師および研究分担医師は、中止基準に該当するため研究を中止した場合には、当該研究対象者に対し適切な措置を講じ、中止の日付・時期、理由、経過等を診療記録等に明記する。

なお、研究治療開始後に同意の撤回があった場合は、その原因が医薬品等の効果不発揮または有害事象（または疾病等）によるものか、あるいは偶発的事象（転居など）によるものかをできるだけ明らかにする。

臨床的に意義のある異常（臨床検査値を含む）が認められる場合は、適切な検査を行い、医学的に許容できる範囲に戻るまで、あるいは担当医師が追跡調査の必要がないと判断するまで、追跡調査を行う。研究中止後も持続する有害事象（または疾病等）については、事象が消失するか、担当医師が追跡調査の必要がないと判断するまで、追跡調査を実施する。研究参加の同意を撤回された研究対象者のこれまで得られたデータは、対象者の意思により、同意撤回書を提出するまでの情報を匿名化して研究解析に用いる、あるいは全ての情報を破棄する。

**８．評価に関する事項**

**8.1　評価指標（エンドポイント）**

**8.1.1　主要評価項目（Primary endpoint）**

手術前後の頚椎JOAスコア改善率

**8.1.2　副次評価項目（Secondary endpoint）**

1. 手術時間
2. 出血量
3. 術後1年・2年時における頚椎JOAスコア改善率のMCID達成割合
4. 術後1年・2年時における健康関連QOL（EQ-5D）
5. 術後1年・2年時における頚部痛、上肢痛、上肢しびれの程度（VAS）
6. 術後1年・2年時における手術前後のNDI
7. 術後1年時・2年時における拡大椎弓保持率（Retention rate） ^3)^
8. 術後1年時・2年時におけるHinge骨折の割合
9. 術後１年時・2年時における骨癒合の割合^4）^
10. 術後１年・2年時における頚椎アライメント（C-SVA, C2-7角、C7 slope）
11. 術後1年時・2年時における傍脊柱筋断面積（C4/5レベル）
12. 術後１年・2年時における硬膜管面積(C3/4,C4/5,C5/6,C6/7レベル)
13. 術後１年・2年時における硬膜管後方圧迫分類
14. 増分費用効果比（ICER）
15. 手術合併症発生割合

・術中合併症割合（手術開始から手術終了（閉創）まで）

・術後早期合併症発生割合（手術終了（閉創）から術後 30 日以内）

・術後晩期合併症発生割合（術後 31 日から術後2年以内）

・死亡率　（全期間：手術関連以外の死亡含む）

・術後2年以内の再手術率

※術中/術後合併症の評価には日本脊椎脊髄病学会データベース(JSSR-DB)の評価基準（https://ssl.jssr.gr.jp/db/）を用いる。

**8.2　評価指標に関する評価、記録**

**＜主要評価項目＞**

手術前後の頚椎JOAスコア改善率

［定義］

手術前と手術後1年または2年時に医師が評価した頚椎JOAスコアを用いる、改善率の計算式は過去の報告^4)^の通り下記の通りの計算式を用いる。

頚椎JOAスコア改善率＝(術後スコア-術前スコア)/(17-術前スコア)×100(%)

主たる解析時点は手術後1年時とする。最終解析として手術後2年時の結果も示す。

副次評価項目３）～13）についても同様。


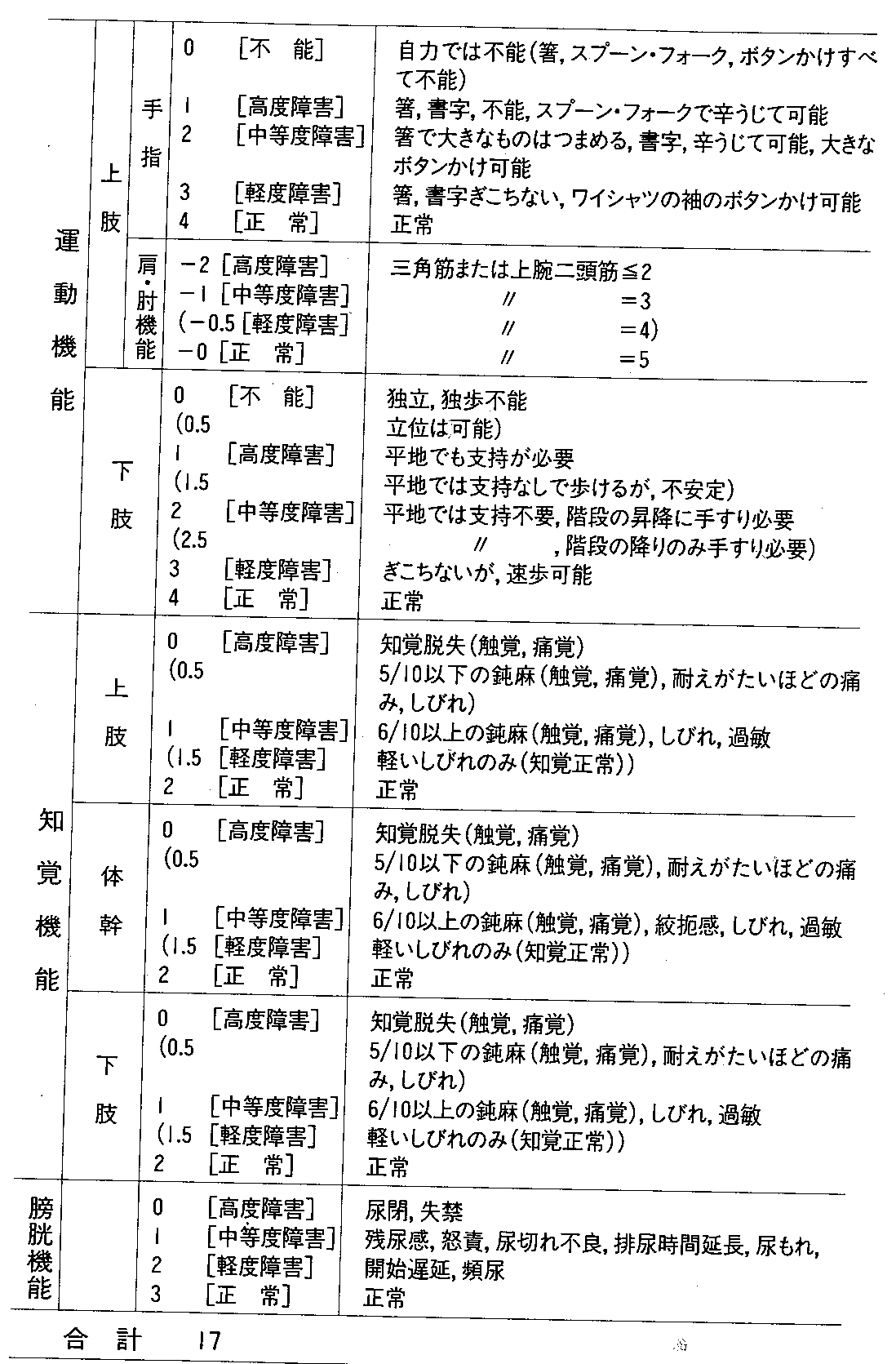


頚椎JOAスコア　(平林　冽ほか：日本整形外科学会頚髄症治療成績判定基準. 日整会誌, 68:490-503.1994)

**＜副次評価項目＞**

1. 手術時間
2. 出血量

［定義］

手術時間は麻酔記録上の手術時間とする。

出血量は麻酔記録上の出血量とする。出血「少量」の記録の場合は出血　10mlとする。

③　手術前後における頚椎JOAスコア改善率のMCID達成割合

［定義］

JOAスコア改善率の定義は＜主要評価項目＞の記載内容を参照のこと。

過去の報告^19)^に従ってJOAスコア改善率のMCIDは52.8%と設定し、各群の術後1年時および2年時のMCID達成率を評価する。

④　健康関連QOL（EQ-5D）

［定義］

日本語版で作成されたアンケート用紙（別紙添付）の患者自身の記入によって取得する。患者が頚髄症による書字困難でアンケート記入ができない場合、家族または医療従事者が記入を補助する。

手術前、術後１年・２年時における健康関連QOLを、EQ-5Dで評価する。なお、EQ-5Dは⑪増分費用効果比を算出する際の効用値としても使用する。

⑤　頚部痛、上肢痛、上肢しびれの程度（VAS）

［定義］

日本語版で作成されたアンケート用紙（別紙添付）の患者自身の記入によって取得する。患者が頚髄症による書字困難でアンケート記入ができない場合、家族または医療従事者が記入を補助する。

手術前、術後１年・２年時における痛み・しびれの程度を、0～100ｍｍのVASで評価する。

⑥　頚椎疾患による健康関連QOL（NDI）

［定義］

日本語版で作成されたアンケート用紙（別紙添付）の患者自身の記入によって取得する。患者が頚髄症による書字困難でアンケート記入ができない場合、家族または医療従事者が記入を補助する。

手術前、術後１年・２年時における頚椎疾患による健康関連QOLをNDI合計点で評価する。


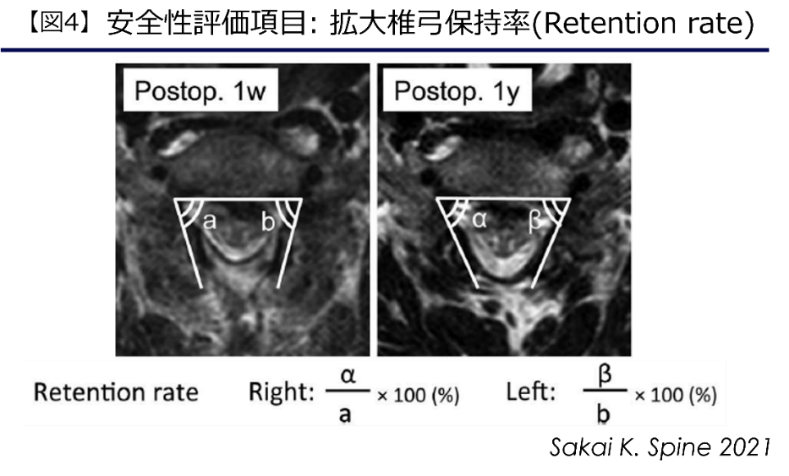
⑦ 術後1年時・2年時における拡大椎弓保持率（Retention rate）

［定義］

術後1年時・2年時のCTから拡大した各椎弓において、過去の報告^7)^に従ってRetention rateを算出する（図4）。また患者ごとにretention rateの平均値を算出する。

⑧ 術後1年時・2年時におけるHinge骨折の割合

⑨　術後１年時・2年時における骨癒合の割合

［定義］

術後1年時・2年時のCT画像から、拡大した各椎弓における、過去の報告^8)^に従ってHinge（椎間関節内側部に作成した溝）の骨折および骨癒合の有無を評価する。いずれも水平断像で評価し、骨折は対象椎弓の骨皮質の連続性が全く断たれたもの、骨癒合は掘削した溝部分が対象椎弓で一部でも骨性に連続性が生じたものと定義し、それぞれの椎弓高位および左右を記録する。

⑩　術後１年・2年時における頚椎アライメント（C-SVA, C2-7角、C7 slope）


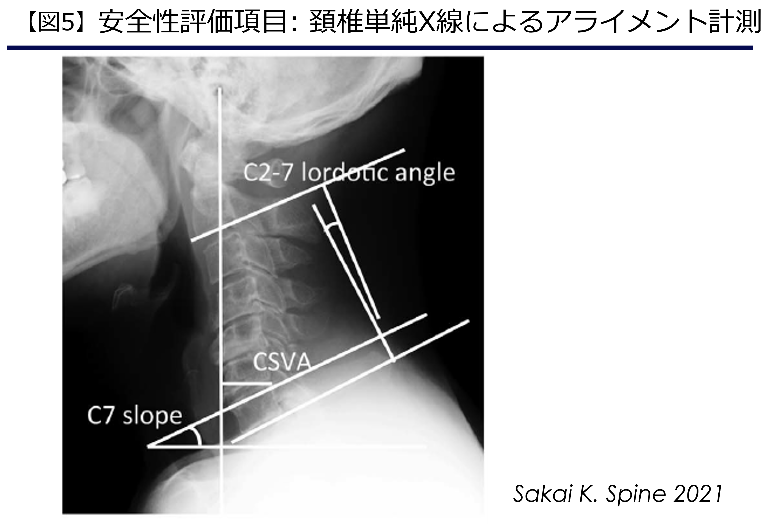
［定義］

術後1年時・2年時における単純X線画像から、頚椎アライメント（C-SVA、C2-7角、C7Slope）を過去の報告^7)^に準じて計測する（図5）。なおC2-7角は前弯を(+)として表記する。

　　　⑪　術後1年時・2年時における傍脊柱筋断面積（C4/5レベル）


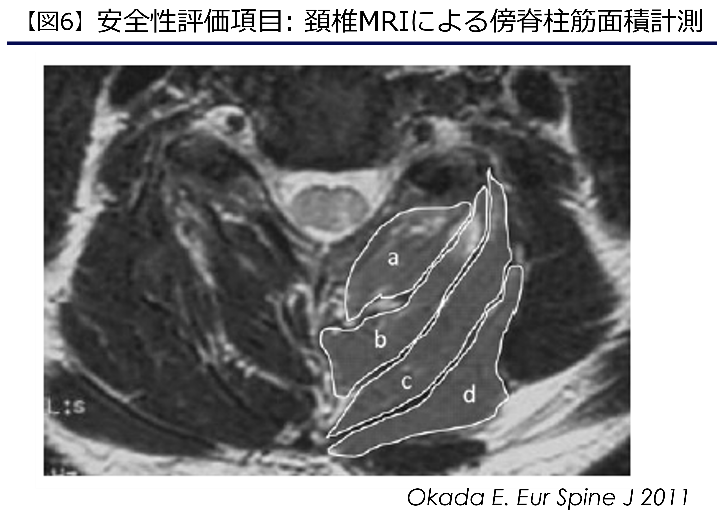
［定義］

T2水平断像のC4/5のスライスにおける傍脊柱筋の断面積を、過去の報告^20)^に準じて計測(mm^2^)し、左右それぞれの合計値を算出する（図6）。

（a：多裂筋、b：頚半棘筋、c：頭半棘筋、d：頭板状筋）

⑫　術後１年・2年時における硬膜管面積(C3/4,C4/5,C5/6,C6/7レベル)

［定義］

T2水平断像のC3/4,C4/5,C5/6,C6/7の各スライスにおける硬膜管の断面積を、過去の報告^21)^に準じて計測(mm^2^)する（図7）。

⑬　術後１年・2年時における硬膜管後方圧迫分類

［定義］

T2矢状断像の中央のスライスにおける硬膜管後方圧迫分類を、過去の報告^22)^に準じて評価する（図7）。


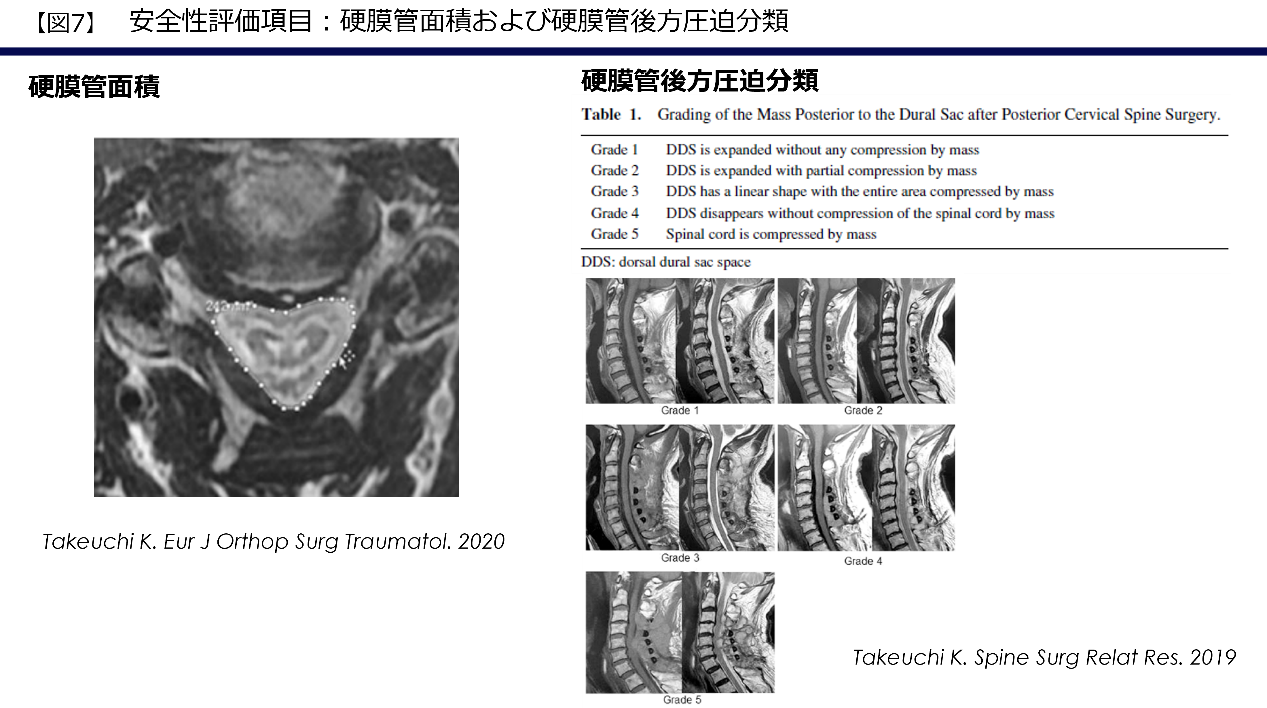


⑭　増分費用効果比（ICER）

［定義］

退院時、術後1年および2年時に各患者の手術施行施設における直接医療費を収集する。直接医療費は、入院治療費、外来治療費を下記の診療を対象として収集し、①②③を合計する。

① DCMに対する手術時の入院治療費（手術関連費用[手術，手術薬剤，麻酔，麻酔薬剤]）、手術を除く診療報酬

②　DCMに対する手術日以降の外来費用（診療，検査，薬剤，その他）

③　有害事象に対する入院再治療費用（手術を除く入院時診療報酬，手術関連費用[手術，手術薬剤，麻酔，麻酔薬剤]）

費用対効果の評価指標は、増分費用対効果(ICER)を用いる。ICERは試験治療群の費用から対照群の費用を差し引いて算出される増分費用を、試験治療群の効果から対照群の効果を差し引いて算出される増分効果で割って算出する。

ICER=$\frac{試験治療群の費用-対照群の費用}{試験治療群の効果-対照群の効果}$

効果指標には、QALY(quality-adjusted life year:質調整生存年)を用いる。QALYはEQ-5Dで算出されるQOLを再手術あるいは死亡患者を考慮に入れたマルコフモデルを用いて算出し、効用値および費用値は中央社会保険医療協議会が推奨するように年間割引率2%を適用する（https://c2h.niph.go.jp/tools/guideline/guideline_ja.pdf）。

⑮ 手術合併症発生割合

手術合併症については術中、および術後早期合併症発生割合（手術終了（閉創）より術後30日以内）、術後晩期合併症発生割合（術後31日目以降から術後2年以内）に分ける。

合併症に関して死亡率（研究期間のあらゆる死亡）と術後2年以内の再手術率は独立して記録する。

**・術中合併症発生割合**

［定義］

解析対象集団を分母とし、手術開始から手術終了（閉創）までに発生した合併症について記載する。特に着目する合併症として、日本脊椎脊髄病学会レジストリー(JSSR-DB: https://ssl.jssr.gr.jp/db/)の登録項目に従って下記（表A）の合併症を抽出し、頻度・割合を記載する。

合併症が発現した場合は、合併症名、転帰、合併症への対応、程度、頚椎手術との関連性、治療内容・治療実施日、入院期間延長の有無等を、診療記録および症例報告書に記載する。

表A　特に着目する術中合併症

| **合併症** | **合併症への対応** |
| --- | --- |
| 硬膜損傷 | 一次修復、フィブリン糊、人工硬膜、脂肪移植、スパイナルドレナージ、その他 |
| 大量出血（2000ml以上） | 同種血輸血、自己血輸血、術中回収血、経過観察、その他 |
| 神経損傷(脊髄)/神経損傷(神経根) | ステロイド投与、経過観察、椎弓切除等、その他 |
| 大血管損傷/椎骨動脈損傷/その他の動脈損傷 | 圧迫止血のみ、当該科による縫合、専門家(血管外科等)による処置、カテーテル、その他 |
| 椎体損傷/関節突起骨折 | インプラントの追加・入れ替え、その他 |
| 高位誤認 | 上位椎(間)への誤認手術、下位椎(間)への誤認手術、その他 |
| 従来法への変更 | 従来法(桐田-宮崎法)：(変更椎レベル)、椎弓切除：(変更椎レベル)、その他 |

**・術後早期合併症発生割合（手術終了（閉創）から術後 30 日以内）**

［定義］

解析対象集団を分母とし、手術終了（閉創）から術後 30 日以内に発生した合併症について記載する。特に着目する合併症として、日本脊椎脊髄病学会レジストリー(JSSR-DB: https://ssl.jssr.gr.jp/db/)の登録項目に従って下記（表B：手術関連合併症、表C：全身合併症）の合併症を抽出し、頻度・割合を記載する。

合併症が発現した場合は、合併症名、転帰、合併症への対応、程度、頚椎手術との関連性、治療内容・治療実施日、入院期間延長の有無等を、診療記録および症例報告書に記載する。

表B　特に着目する術後早期合併症(手術関連)

| **合併症** | **合併症への対応** |
| --- | --- |
| 血管損傷(大血管損傷/椎骨動脈損傷/その他の血管損傷) | 経過観察、当該科による処置、専門家(血管外科等)による処置、カテーテル、その他 |
| 硬膜外血種 | 椎弓切除、血腫除去 |
| 神経症状・筋力関連(術後上肢麻痺/術後下肢麻痺/術後感覚神経麻痺/膀胱直腸障害/上記以外の合併症) | 経過観察、再手術、ステロイド投与、その他薬物治療、その他 |
| 脊柱支持組織損傷(椎体損傷/関節突起骨折) | インプラントの追加・入れ替え/その他 |
| 硬膜損傷(髄液漏/髄膜炎/頭蓋内病変[硬膜下血腫等]) | 保存(経過観察/スパイナルドレナージ)、再手術(追加処置、硬膜縫合、フィブリン糊、人工硬膜、脂肪移植、スパイナルドレナージ)、抗生剤投与 |
| 固定金属関連(Implant破損、Implant脱転、スクリューの位置不良) | 経過観察、インプラント入れ替え、インプラント追加、その他 |
| Surgical Site Infection(浅層/深層) | 洗浄、かん流、インプラント抜去、その他 |
| 高位誤認 | 上位椎(間)への誤認手術、下位椎(間)への誤認手術、その他 |
| その他(体位による術後障害)、眼障害[角膜損傷など])、失明、嚥下障害、その他 | 経過観察、追加手術 |

表C　特に着目する術後早期合併症(全身合併症)

| **合併症** | **合併症への対応** |
| --- | --- |
| 急性心不全 | 経過観察、保存的治療、内視鏡治療・IVR等、手術治療、その他 |
| 心筋梗塞 | 経過観察、保存的治療、内視鏡治療・IVR等、手術治療、その他 |
| 肺梗塞 | 経過観察、保存的治療、内視鏡治療・IVR等、手術治療、その他 |
| 肺塞栓 | 経過観察、保存的治療、内視鏡治療・IVR等、手術治療、その他 |
| 末梢静脈血栓症(症候性) | 経過観察、保存的治療、内視鏡治療・IVR等、手術治療、その他 |
| 呼吸不全 | 経過観察、保存的治療、内視鏡治療・IVR等、手術治療、その他 |
| 誤嚥性肺炎 | 経過観察、保存的治療、内視鏡治療・IVR等、手術治療、その他 |
| 肺炎 | 経過観察、保存的治療、内視鏡治療・IVR等、手術治療、その他 |
| 無気肺 | 経過観察、保存的治療、内視鏡治療・IVR等、手術治療、その他 |
| 肺水腫 | 経過観察、保存的治療、内視鏡治療・IVR等、手術治療、その他 |
| 脳梗塞 | 経過観察、保存的治療、内視鏡治療・IVR等、手術治療、その他 |
| 術後せん妄 | 経過観察、保存的治療、内視鏡治療・IVR等、手術治療、その他 |
| 認知症 | 経過観察、保存的治療、内視鏡治療・IVR等、手術治療、その他 |
| 腸閉塞 | 経過観察、保存的治療、内視鏡治療・IVR等、手術治療、その他 |
| 偽膜性腸炎 | 経過観察、保存的治療、内視鏡治療・IVR等、手術治療、その他 |
| 消化管穿孔・胃穿孔 | 経過観察、保存的治療、内視鏡治療・IVR等、手術治療、その他 |
| 膵炎 | 経過観察、保存的治療、内視鏡治療・IVR等、手術治療、その他 |
| 肝炎 | 経過観察、保存的治療、内視鏡治療・IVR等、手術治療、その他 |
| 胆嚢炎 | 経過観察、保存的治療、内視鏡治療・IVR等、手術治療、その他 |
| 急性腎不全 | 経過観察、保存的治療、内視鏡治療・IVR等、手術治療、その他 |
| 尿路感染症 | 経過観察、保存的治療、内視鏡治療・IVR等、手術治療、その他 |
| DIC | 経過観察、保存的治療、内視鏡治療・IVR等、手術治療、その他 |
| 敗血症 | 経過観察、保存的治療、内視鏡治療・IVR等、手術治療、その他 |
| 入院中の転倒・転落による骨折 | 経過観察、保存的治療、内視鏡治療・IVR等、手術治療、その他 |
| その他 | 経過観察、保存的治療、内視鏡治療・IVR等、手術治療、その他 |

- **術後晩期合併症発生割合（術後 31 日から術後2年以内）**

［定義］

解析対象集団を分母とし、術後 31 日から術後2年以内に発生した合併症について記載する。特に着目する合併症として、下記（表D）の合併症を抽出し、頻度・割合を記載する。

合併症が発現した場合は、合併症名、転帰、合併症への対応、頚椎手術との関連性、治療内容・治療実施日、入院期間延長の有無等を、診療記録および症例報告書に記載する。

**表D　特に着目する術後晩期合併症**

| **合併症** | **合併症詳細および対応** |
| --- | --- |
| 神経症状 | 部位（手術椎間、隣接椎間、他部位）、責任高位、対応処置、転帰 |
| 局所後弯増悪 | 高位、対応処置、転帰 |
| すべり増悪 | 高位、前方・後方、対応処置、転帰 |
| 全身合併症/併存症 | 名称、対応処置、転帰 |
| Instrument関連 | ゆるみ・脱転、対応処置、転帰 |
| 感染 | 表層・深層、対応処置、転帰 |
| その他 | 合併症名、対応処置、転帰 |

**8.3　評価指標に関する解析の方法ならびに時期**

「10．統計的な解析に関する事項」参照。

**９．有害事象・手術合併症・不具合の記録、報告等に関する事項**

**9.1　有害事象・手術合併症・不具合に関する記録、報告、解析の方法**

**１）有害事象**

有害事象とは、実施された研究との因果関係の有無を問わず、研究対象者に生じたすべての好ましくない、または意図しない傷病若しくはその徴候（臨床検査値の異常を含む。）をいう。

登録前から発現している症状や疾病は併存疾患として取り扱い、有害事象としない。ただし、手術合併症（後述）も有害事象に含まれるが、本研究では、8.2⑨表A～Dのとおり術中手術合併症と術後早期合併症（手術関連・全身合併症）術後晩期合併症の有害事象を分けて集計することとする。

評価の対象とする期間は、手術後2年までとする。

有害事象が発現した場合は、合併症名、転帰、合併症への対応、頚椎手術との関連性、治療内容・治療実施日、入院期間延長の有無等を、診療記録および症例報告書に記載する。また、定期的に発生状況を集計し、モニタリング報告書に記載する。

**２）手術合併症**

本研究における手術合併症とは、プロトコール治療として実施した手術に関連して研究対象者に生じた好ましくない、または意図しない傷病若しくはその徴候（臨床検査値の異常を含む。）と定義する。手術合併症は、全身麻酔合併症、術中合併症（手術開始から手術終了（閉創）まで）、術後早期合併症（手術終了（閉創）から術後 30 日以内）、術後晩期合併症（術後 31 日から術後2年以内）に分類する。評価の対象とする期間は手術後2年までとする。

手術合併症が発現した場合は、合併症名、転帰、合併症への対応、程度、治療内容・治療実施日、入院期間延長の有無等を、診療記録および症例報告書に記載する。また、定期的に発生状況を集計し、モニタリング報告書に記載する。

程度は、有害事象共通用語規準（Common Terminology Criteria for Adverse Events：CTCAE）version5.0に基づき判定する。

Grade 1：軽症、無症状または軽度の症状がある、臨床所見または検査所見のみ、治療不要

Grade 2：中等症、最小限・局所的・非侵襲的治療を要する、年齢相応の身の回り以外の日常動作の制限

Grade 3：重症または医学的に重大であるが直ちに生命を脅かすものではない、入院または入院期間の延長を要する、活動不能・動作不能、身の回りの日常生活動作の制限

Grade 4：生命を脅かす、緊急処置を要する

Grade 5：有害事象による死亡

1. **重篤な有害事象・手術合併症**

以下のいずれかに該当するものを重篤な有害事象・手術合併症とする。

1） 死亡

2） 死亡につながるおそれのある疾病等

3） 治療のために医療機関への入院または入院期間の延長が必要とされる疾病等

4） 障害

5） 障害につながるおそれのある疾病等

6） 1）から 5）に準じて重篤である疾病等

7） 後世代における先天性の疾病または異常

評価の対象とする期間は、手術後２年までとする。

有害事象・手術合併症の事象名、合併症名、転帰、合併症への対応、治療内容・治療実施日、入院期間延長の有無等を、診療記録および症例報告書に記載する。また、定期的に発生状況を集計し、モニタリング報告書に記載する。

**４）不具合**

不具合とは、機器の破損、作動不良等広く品質、安全性、性能等に関する機器の具合がよくないことをいい、設計、交付、保管、使用のいずれの段階によるものであるかを問わない。本研究では、対照群（プレート法）に使用するスクリューおよびミニプレート、試験治療群（縫合糸アンカー法）に使用する縫合糸アンカーについて、不具合に該当する事象が発生した場合には、診療記録および症例報告書に記載する。また、定期的に発生状況を集計し、モニタリング報告書に記載する。また、必要に応じて、当該機器の製造販売企業と不具合に関する情報を共有する（個人情報を含まないような形で）。

**9.2　予期される有害事象**

　　本研究における予期される有害事象（手術合併症を含む）は以下のとおり。

1）術中合併症

- - - 硬膜損傷
- 大量出血(2000ml以上)
- 神経損傷(脊髄)/神経損傷(神経根)
- 大血管損傷/椎骨動脈損傷/その他の動脈損傷
- 椎体損傷/関節突起骨折
- 高位誤認
- 従来法への変更

2）術後早期合併症 (手術関連)

- - - 血管損傷(大血管損傷/椎骨動脈損傷/その他の血管損傷)
- 硬膜外血種
- 神経症状・筋力関連(術後上肢麻痺/術後下肢麻痺/術後感覚神経麻痺/膀胱直腸障害/上記以外の合併症)
- 脊柱支持組織損傷(椎体損傷/関節突起骨折)
- 硬膜損傷(髄液漏/髄膜炎/頭蓋内病変[硬膜下血腫等])
- 固定金属関連(Implant破損、Implant脱転、スクリューの位置不良)
- Surgical Site Infection(浅層/深層)
- 高位誤認
- その他(体位による術後障害)、眼障害[角膜損傷など])、失明、嚥下障害

術後早期合併症(全身合併症)

- 急性心不全
- 心筋梗塞
- 肺梗塞
- 肺塞栓
- 末梢静脈血栓症(症候性)
- 呼吸不全
- 誤嚥性肺炎
- 肺炎
- 無気肺
- 肺水腫
- 脳梗塞
- 術後せん妄
- 認知症
- 腸閉塞
- 偽膜性腸炎
- 消化管穿孔・胃穿孔
- 膵炎
- 肝炎
- 胆嚢炎
- 急性腎不全
- 尿路感染症
- DIC
- 敗血症
- 入院中の転倒・転落による骨折
- その他

1. 術後晩期合併症

- 神経症状
- 局所後弯増悪
- すべり増悪
- 全身合併症/併存症
- Instrument関連（ゆるみ・脱転）
- 感染（表層・深層）
- その他

**9.3　有害事象・手術合併症・不具合発生時の対応**

**9.3.1　有害事象・手術合併症・不具合の発生時の対応**

担当医師は、有害事象（手術合併症を含む。以下同様）・不具合を認めたときは、直ちに適切な処置を行うとともに、問診記録等の原資料ならびに症例報告書に齟齬なく記載する。また、プロトコール治療を中止した場合や、有害事象に対する治療が必要となった場合には、研究対象者にその旨を伝える（有害事象発生時の補償については、「15. 金銭の支払い及び保障に関する事項」参照）。

**9.3.2　重篤な有害事象・手術合併症・不具合の発生時の対応**

研究期間中のすべての重篤な有害事象（手術合併症を含む。以下同様）、研究終了（中止）後に研究との関連性が疑われる重篤な有害事象については、速やかに研究対象者に適切な処置を施すとともに、研究代表者に報告する。研究代表者は、所定の書式（【安全性報告書式2】等）を用いて速やかに（原則として第一報は2週間以内に）研究機関の長（病院長）に報告するとともに、倫理審査委員会等、臨床研究監視委員会に報告＊する。また、速やかにすべての研究機関の長（病院長）にも通知する。

＊：東京科学大学「倫理審査申請システム」から報告する。

**9.3.3　予測できない重篤な有害事象・手術合併症・不具合の発生時の対応**

予測できない重篤な有害事象（手術合併症を含む。以下同様）が発生した場合には、速やかに研究対象者に適切な処置を施すとともに、研究代表者に報告する。研究代表者は、所定の書式（【書式安1】等）を用いて速やかに（原則として第一報は2週間以内に）研究機関の長（病院長）に報告するとともに、倫理審査委員会等、臨床研究監視委員会に報告＊する。また、速やかにすべての研究機関の長（病院長）にも通知する。

＊：東京科学大学「倫理審査申請システム」から報告する。

※「9.2 予期される有害事象」に記載の事象については、既知／予測できる事象とみなす。

## **9.4****有害事象・手術合併症・不具合発生後の研究対象者の観察**

有害事象（手術合併症を含む。以下同様）発生後の研究対象者の観察については、有害事象が消失・回復するまで、あるいは担当医師が追跡の必要がないと判断するまで、追跡調査を行う。

**10．統計的な解析に関する事項**

本研究の統計解析計画の概要を以下に示す。なお、統計解析計画の詳細は、別途、統計解析計画書に定める。統計解析計画書において、解析方法等について本研究計画書の概要から変更する場合があるが、主要評価項目の定義や解析方法等、本臨床研究の結果解釈に大きく影響する内容が変更される場合には、本研究計画書を改訂する。

**10.1　解析対象集団**

**10.1.1　最大の解析対象集団 (Full Analysis Set、 FAS)**

本研究に登録され無作為化された症例のうち、プロトコール治療（頚椎椎弓形成術）を受けた症例の集団と定義する。ただし、無作為化後のデータが全くない症例は除外する。

**10.1.2　安全性解析対象集団 (Safety Analysis Set、 SAS)**

本研究に登録され無作為化された症例のうち、プロトコール治療（頚椎椎弓形成術）が行われたすべての症例の集団と定義する。

**10.2　目標登録数と設定根拠**

目標登録数：216例

（対照群［プレート法］：108例、試験治療群［縫合糸アンカー法］：108例）

【設定根拠】

主要評価項目である手術前後の頚椎JOAスコア改善率について、本研究と同様の患者集団を対象とした済生会川口総合病院の後ろ向きパイロット研究では、プレート法で49.9±22.8%（21例）、縫合糸アンカー法で52.6±32.9%（20例）であった（未発表データ）。さらに、過去の報告^3)^では、圧迫性脊髄症におけるMCIDは52.8%と報告されている。

パイロット研究および過去のMCIDに関する結果から、本研究における対照群（プレート法）における頸椎JOAスコア改善率を50％(標準偏差40％)、非劣性マージンを20%に設定し、有意水準（片側）0.025、検出力0.9とすると、必要症例数は両群合わせて172例となる。約20％の脱落を見込んで、目標症例数を両群合わせて216名と設定した。

当該目標登録数は見込み登録数として取り扱い、当該見込み登録数を超過することを許容する。ただし、研究代表者は、本研究における各実施医療機関の実登録数を積算した症例数が本研究の目標登録数を超過しないように管理する。研究全体の目標登録数を追加する必要がある場合は、事前に倫理審査委員会等の承認を得る。

**10.3　症例の取扱い**

原則として登録された症例については、研究代表者と統計解析責任者等が協議の上、症例の取扱いを決定する。新たな問題が起こった場合の症例の取扱いについても、研究代表者および統計解析責任者等が協議の上決定し、いずれの場合も、症例取扱いの決定内容について記録に残す。

**10.4　データの取扱い**

データ集計・解析時におけるデータの取扱いについては、原則として以下に示す通りとする。疑義が生じた場合は、研究代表医師および統計解析責任者が協議の上、決定する。欠測値の補完は行わない。詳細については、別途、統計解析計画書に記載する。

**10.5　統計解析項目および解析計画**

全ての有効性評価について、FASを対象とした解析を実施する。

安全性の解析は、SASにおける解析を実施する。

統計解析の詳細はデータ固定前に別途作成する統計解析計画書に規定する。

**10.5.1　研究対象者の背景の要約**

各解析対象集団における研究対象者の背景データの分布および要約統計量を、割付群ごとに算出する。名義変数および順序変数については、カテゴリの頻度および割合を割付群ごとに示す。連続変数については要約統計量（例数、平均値、標準偏差、最小値、中央値、最大値）を割付群ごとに算出する。

**10.5.2　主要評価項目の解析**

・手術前後の頚椎JOAスコア改善率

FASを対象に、術後1年時における頚椎JOAスコア改善率について、共分散分析を用いて、各群の最小二乗平均値とその95%信頼区間を算出する。また、最小二乗平均の群間差（縫合糸アンカー法 – プレート法）とその95%信頼区間、およびp値を算出する。解析モデルには、群、術前JOAスコア、年齢（64歳以下、65～74歳、75歳以上）、性別、術前診断（頚椎症性脊髄症（CSM）、後縦靭帯骨化症（OPLL））を共変量として含める。

　術後1年時における最小二乗平均の群間差について、95%信頼区間の下限が–20%を上回っていた場合、縫合糸アンカー法はプレート法に対して非劣性であることが検証されたと判断する。また、副次的解析として、FASを対象に、術後1年時および術後２年時における手術前後の頚椎JOAスコア改善率を応答変数としたMixed-effects Model for Repeated Measures（MMRM）による解析を行う。解析モデルには、群、時点、群と時点の交互作用、術前JOAスコア、年齢（64歳以下、65～74歳、75歳以上）、性別、術前診断（CSM、OPLL）を固定効果として含める。上記のモデルを用いて、術後1年時および術後２年時における手術前後の頚椎JOAスコア改善率の最小二乗平均値と95%信頼区間を算出する。また、術後1年時および術後２年時における手術前後の頚椎JOAスコア改善率の群間差とその95%信頼区間、p値を算出する。

**10.5.3　副次評価項目の解析**

①　手術時間

②　出血量

FASを対象に、要約統計量（例数、平均値、標準偏差、最小値、中央値、最大値）を群別に算出する。群間比較としてStudentのt検定を行い、p値を算出する。

③　手術前後の頚椎JOAスコア改善率のMCID達成割合

FASを対象に、術後1年時、および術後2年時における頚椎JOAスコア改善率のMCID（JOAスコア改善率52.8％）の達成割合を群別に算出する。群間比較として、MCID（JOAスコア改善率52.8％）の達成割合の差およびその95％信頼区間を算出し、Fisherの正確検定を行う。信頼区間の算出にはClopper-Pearson法を用いる。

④　健康関連QOL（EQ-5D）

⑤　頚部痛、上肢痛、上肢しびれの程度（VAS）

⑥　頚椎疾患による健康関連QOL（NDI）

FASを対象に、各評価項目の術前から術後1年時までの変化量について、主要評価項目と同様の解析を行う。

また、各評価項目の術前から術後1年時および術後２年時までの変化量について、副次評価項目②と同様の解析を行う。

⑦　術後1年時・2年時における拡大椎弓保持率（Retention rate）

FASを対象に、拡大椎弓保持率の術前から術後1年時までの変化量について、主要評価項目と同様の解析を行う。

また、拡大椎弓保持率の術前から術後1年時および術後２年時までの変化量について、副次評価項目②と同様の解析を行う。

⑧　術後1年時・2年時におけるHinge骨折の割合

⑨　術後１年時・2年時における骨癒合の割合

FASを対象に、術後1年時、および術後2年時における各評価項目の割合を群別に算出する。群間比較として、各評価項目の割合の差およびその95％信頼区間を算出し、Fisherの正確検定を行う。信頼区間の算出にはClopper-Pearson法を用いる。

　⑩　術後１年・2年時における頚椎アライメント（C-SVA, C2-7角、T1 slope）

FASを対象に、各評価項目の術前から術後1年時までの変化量について、主要評価項目と同様の解析を行う。

また、各評価項目の術前から術後1年時および術後２年時までの変化量について、副次評価項目②と同様の解析を行う。

⑪　術後1年時・2年時における傍脊柱筋断面積（C4/5レベル）

FASを対象に、傍脊柱筋断面積の術前から術後1年時までの変化量について、主要評価項目と同様の解析を行う。

また、傍脊柱筋断面積の術前から術後1年時および術後２年時までの変化量について、副次評価項目②と同様の解析を行う。

⑫　術後１年・2年時における硬膜管面積(C3/4,C4/5,C5/6,C6/7レベル)

FASを対象に、FASを対象に、硬膜管面積の術前から術後1年時・2年時までの変化量について、主要評価項目と同様の解析を行う。

また、硬膜管面積の術前から術後1年時および術後２年時までの変化量について、副次評価項目②と同様の解析を行う。

⑬　術後１年・2年時における硬膜管後方圧迫分類

FASを対象に、術後１年・2年時における硬膜管後方圧迫分類をGrade1-3のmild, Grade4-5のsevereに分類する。術後１年・2年時における硬膜管後方圧迫分類のsevereの割合を群別に算出する。群間比較として、各評価項目の割合の差およびその95％信頼区間を算出し、Fisherの正確検定を行う。信頼区間の算出にはClopper-Pearson法を用いる。

⑭　増分費用効果比（ICER）

対照群に対する試験治療群のICERを算出する。

算出方法の詳細については8.1.2章参照のこと。

⑮　手術合併症発生割合

SASを対象に、以下の区分別に、各合併症の発現例数および割合を割付群ごとに算出する。

・術中合併症割合(手術開始から手術終了（閉創）まで)

・術後早期合併症発生割合（手術終了（閉創）から術後 30 日以内）

・術後晩期合併症発生割合（術後 31 日から術後2年以内）

**10.5.4　サブグループ解析**

以下に示す因子に基づくサブグループ解析を行う。これらの解析は十分な検出力を担保して行うものでなく、また多重性の調整も行わないため、各サブグループ解析の結果はあくまで探索的な結果と解釈する。その他、解析時点において臨床的意義が期待される臨床病理学的特徴や予後等に関連したサブグループ解析を行う。

＜サブグループ解析を予定している因子＞

・ 年齢（64歳以下／65歳-74歳／75歳以上）

・ 性別（男性／女性）

・ BMI（25以下／25より大きい）

・ 術前診断（CSM／OPLL）

・ 拡大椎弓数（2／3／4）

・　硬膜後方圧迫分類（Grade1-3／Grade4-5）

**10.6　中間解析**

中間解析は実施しない。

**10.7　主たる解析**

主要評価項目および有効性の副次評価項目に関する主たる解析は、術後1年時のデータを用いて実施する。最終登録症例の術後1年時の評価を完了したのちに、主たる解析を実施する。

詳細はデータ固定前に別途作成する統計解析計画書に規定する。

**10.8　最終解析**

最終登録症例の術後２年時の評価の終了を以て追跡期間終了とする。追跡期間終了後、データが得られた症例が固定された後に、最終解析を行う。統計解析責任者が「解析報告書」をまとめ、研究代表者に提出する。

**11．原資料等の閲覧に関する事項**

本研究における原資料とは、診療録、各種検査データ、手術記録、投薬記録、患者評価シート等とする。

研究責任者および研究機関の管理者は、本研究に関するモニタリングおよび監査ならびに倫理審査委員会および規制当局による調査を受け入れ、その際に、本研究に関する原資料等の全ての資料を直接閲覧に供することを保証する。

診療記録に記載が無く症例報告書に記載されたデータのうち、以下に示す記載項目は、症例報告書の記載を原資料とする。

1）合併症・有害事象の重篤度、程度、転帰、転帰日、プロトコール治療等との因果関係、因果関係を判定した理由

2）中止日、中止理由、中止の原因となった合併症・有害事象、中止後の経過および追跡調査の結果

3）担当医師コメント

**12．品質管理および品質保証に関する事項**

**12.1　モニタリング**

研究代表者は、研究が安全に、かつ研究計画書に従って実施されているか、データが正確に収集されているかを確認する目的で、モニタリングを実施する。また、モニタリングを担当するモニタリング担当者を指名する。モニタリング担当者は、研究期間を通じて本研究が最新の研究計画書および研究関連指針を遵守して実施されていることを確認し、その確認した結果について、モニタリング報告書を作成し、研究代表者に提出する。研究代表者はモニタリング報告書を検討し、問題点を研究機関の研究者と情報共有し、その改善に努める。モニタリング担当者は、モニタリングの際に得た研究対象者の個人情報を漏らしてはならない。

① 登録状況：登録数－累積／期間別、群／施設別

② 適格性：不適格例／不適格の可能性のある患者：群／施設

③ 治療前背景因子：群

④ プロトコール治療中／治療終了の別、中止／終了理由：群／施設

⑤ プロトコール逸脱：群／施設

⑥ 重篤な有害事象：群／施設

⑦ 有害事象・不具合：群

⑧ 全生存期間：全登録例

⑨ その他、試験の進捗や安全性に関する問題点

**12.2　監査**

本研究は、現在保険診療下で実施されている手術手技に関する比較試験であることから、研究者等が品質を確保することとし、監査は実施しない。ただし、研究対象者の安全性に重大な影響を与える事象等が発生した場合等は、必要に応じて監査の実施を検討する。

# **13．倫理的な配慮に関する事項**

**13.1　法令等の遵守**

本研究に関係するすべての研究者は、「ヘルシンキ宣言」（2013年10月、日本医師会訳）^A）^及び「人を対象とする生命科学・医学系研究に関する倫理指針」（令和４年３月10日一部改正）^B）^ならびに関連通知を遵守して本研究を実施する。

A） http://dl.med.or.jp/dl-med/wma/helsinki2013j.pdf

B） https://www.mhlw.go.jp/content/000909926.pdf

**13.2　予期される利益、負担および不利益**

１）予期される利益

本研究に参加することにより研究対象者に直接の利益は生じない。研究成果により将来の医療の進歩に貢献できる可能性がある。

２）予期される不利益

対照治療（プレート法）と試験治療（縫合糸アンカー法）の技術的な難易度の比較について、脊椎手術に習熟している医師が施行する場合差はなく、また合併症の発生頻度や安全性その他についても差はないと考えられる。実際に、海外で行われた片開き式椎弓形成術のプレート法と縫合糸アンカー法の短期成績に関するメタアナライシスでは、手術時間や出血量、JOAスコアにおいてプレート法群と縫合糸アンカー法群で有意差はないと報告されている^23^。このことから、本研究に参加することに伴う特段の不利益はないと考える。

また、本研究では、各研究機関内に日本脊椎脊髄病学会　脊椎脊髄外科指導医が勤務していることを研究参加の条件とする。これにより、手術の質および安全性を担保し、研究対象者のリスクを最小化するとともに、質の高い臨床研究とすることが可能と考える。

３）予期される負担

本研究に参加することによる、入院期間、来院回数、検査回数、診察・検査時間などは、通常診療と同程度である。なお、本研究に参加することによる研究対象者の費用負担は発生しない。（「15.　金銭の支払いおよび補償に関する事項」参照）。

４）リスクを最小化する方策

本研究では、椎弓形成手術が必要であり、もともと手術を予定している患者を研究対象者としているため、研究参加に伴い、通常診療を超える身体的リスクが生じる可能性は低いと考えているが、追跡期間を通じて、担当医師は、研究対象者の状態に十分に注意し、手術合併症・有害事象の早期発見に努める。

プロトコール治療に起因する合併症・有害事象が起きた場合は、対応を個別に検討する。研究対象者の個人情報の保護に対する配慮については、「21. 個人情報等の取扱い」参照。

**13.3　研究対象者に係わる遺伝的特徴等に関する研究結果や偶発的初見の取扱い**

本研究では研究対象者の健康、遺伝学的特徴に関する重要な知見が得られるような検査・解析は実施しない。

# **14．情報・試料等の収集、保管、廃棄に関する事項**

**14.1　情報の収集、保管、廃棄**

本研究では、収集する情報（研究データ）については、電子データ管理システム（EDCシステム）「eACReSS」に入力し、管理する。入力された情報は、東京科学大学病院のeACReSSサーバ内に保管される。

＊：「eACReSS」は、大学病院臨床試験アライアンス事業にて整備された臨床研究データを管理する専用システムである。

1. 保管場所：東京科学大学病院「eACReSS」サーバ内

※試験終了後は、光ディスク等を用いて研究代表者に研究データを移管する。

1. 保管責任者：吉井 俊貴（研究代表者）
2. 保存期間：主たる論文等の発表後10年以上　（本学規定に従う）
3. 廃棄方法：保管期間終了後に廃棄する場合は、復元不可能な状態に処理して廃棄する。
4. 二次利用の可能性：　■あり　　　□なし

なお、EDCシステムに入力する以外の研究実施に係る書類、原資料（問診記録、ワークシート等）等の紙媒体については、各研究研究機関の施錠可能な保管庫で保管する。

① 保管責任者：各研究研究機関の研究責任者

② 保存期間：研究終了後10年間

③ 廃棄方法：シュレッダー等の復元不可能な状態に処理して廃棄する。

また、将来の研究のために、本研究で得られた研究対象者のデータを利用する可能性がある。これらの将来の研究を実施する場合には、別途研究計画書を作成し、改めて倫理審査委員会等で審査を受けた上で実施する（「21.2 データの二次利用」参照）。

**14.2　試料の収集、保管、廃棄**

本研究では、研究対象者から血液、組織、細胞、体液、排泄物及びこれらから抽出したDNA等を採取・使用する予定はない。

**15．金銭の支払いおよび補償に関する事項**

**15.1　金銭の支払い（研究対象者の費用負担）**

本研究におけるプロトコール治療（プレート法または縫合糸アンカー法による椎弓形成手術）は、保険診療の範囲内であり、本研究に参加することによる、研究対象者の追加の費用負担は発生しない。研究対象者の費用負担は通常診療で発生する費用の範囲内である。

**15.2　補償に関する事項**

本研究は、通常の医療の範囲を超える医療行為を行わないが、臨床研究保険に加入している。未知の副作用等が発生し、入院相当の治療を行った場合には、加入する臨床研究保険により医療費・医療手当が支払われる。その他の本研究の実施に伴い生じた健康被害に対しては医療の提供等により適切な措置を講じる他、本研究における通常の医療行為に起因する研究対象者の健康被害に備えて、研究に参加する医師は医師賠償責任保険には必ず加入する。

**16．情報の公表**

- 本研究は、研究の実施に先立ち、臨床研究データベース（jRCT臨床研究等提出・公開システム（<https://jrct.niph.go.jp/>））に研究内容の登録を行う。
- 研究の成果は国内外の研究会・学会、論文等にて公表されるが、その際は、研究対象者を特定できる情報を含まない形で公表される。

**17．実施期間**

総研究期間：2025年10月17日(jRCT公表日)～2031年3月31日（5.5年）

（予定登録期間：３年、追跡期間：手術後2年、解析期間：0.5年）

**18．研究対象者に対する説明および同意**

すべての研究対象者から自由意思に基づく文書による同意を得る。同意は、同意文書に署名することで文書として記録される。本研究計画書を承認する同一の倫理審査委員会等により承認された説明文書及び同意文書を用いる。

研究対象者は、正当な根拠がなくとも、自由に研究への参加を拒否するまたはいつでも研究を中止することができる。研究対象者は拒否または中止により不利益を被ることはない。

担当医師（もしくは研究代表者が指名した研究協力者）が適切かつ重要な情報を提供し、平易な言葉を用いて十分に説明した後に、同意文書に、研究対象者及び同意取得者（担当医師）の両者が自ら署名し、日付を記入する。なお、研究協力者が補足的に説明した場合はその研究協力者も署名し、日付を記入する。研究対象者は説明文書及び署名した同意文書の写しを受取り、原本は研究機関にて保管する。

研究計画書が改訂され、研究の手順が大幅に追加または変更される場合、研究対象者は追加の同意文書への署名を依頼されることがある。

《研究対象者に対する説明事項》

同意説明文書には、以下の事項を記載する。

1. 研究の名称及び当該研究の実施について研究機関の長の許可を受けている旨
2. 研究機関の名称及び研究責任者の氏名（多機関共同研究を実施する場合には、共同研究機関の名称及び共同研究機関の研究責任者の氏名を含む）
3. 研究の目的及び意義
4. 研究の方法（研究対象者から取得された試料・情報の利用目的及び取扱いを含む）及び期間
5. 研究対象者として選定された理由
6. 研究対象者に生じる負担並びに予測されるリスク及び利益
7. 研究が実施又は継続されることに同意した場合であっても随時これを撤回できる旨（研究対象者等からの撤回の内容に従った措置を講じることが困難となる場合があるときは、その旨及びその理由を含む）
8. 研究が実施又は継続されることに同意しないこと又は同意を撤回することによって研究対象者等が不利益な取扱いを受けない旨
9. 研究に関する情報公開の方法
10. 研究対象者等の求めに応じて、他の研究対象者等の個人情報等の保護及び当該研究の独創性の確保に支障がない範囲内で研究計画書及び研究の方法に関する資料を入手又は閲覧できる旨並びにその入手又は閲覧の方法
11. 個人情報等の取扱い（個人が特定できないような措置を行う場合にはその方法、仮名加工情報又は匿名加工情報を作成する場合にはその旨を含む）
12. 試料・情報の保管及び廃棄の方法
13. 研究の資金源その他の研究機関の研究に係る利益相反、及び個人の収益その他の研究者等の研究に係る利益相反に関する状況
14. 研究により得られた結果等の取扱い
15. 研究対象者等及びその関係者からの相談等への対応（遺伝カウンセリングを含む）
16. 研究対象者等に経済的負担又は謝礼がある場合には、その旨及びその内容
17. 通常の診療を超える医療行為を伴う研究の場合には、他の治療方法等に関する事項
18. 通常の診療を超える医療行為を伴う研究の場合には、研究対象者への研究実施後における医療の提供に関する対応
19. 侵襲を伴う研究の場合には、当該研究によって生じた健康被害に対する補償の有無及びその内容
20. 研究対象者から取得された試料・情報について、研究対象者等から同意を受ける時点では特定されない将来の研究のために用いられる可能性又は他の研究機関に提供する可能性がある場合には、その旨と同意を受ける時点において想定される内容

㉑ 侵襲（軽微な侵襲を除く）を伴う研究であって介入を行うものの場合には、研究対象者の秘密が保全されることを前提として、モニタリングに従事する者及び監査に従事する者並びに倫理審査委員会が、必要な範囲内において当該研究対象者に関する試料・情報を閲覧する旨

**19．利益相反に関する事項**　

本研究は、東京科学大学　整形外科学分野の研究費（運営費およびオリンパス・テルモバイオマテリアル株式会社との共同研究費等）により実施する。手術に用いる機器の製造販売企業からの資金や便益等の提供はなく、研究者が企業とは独立に、研究計画立案・実施・結果の解析・結果の公表を行うものである。なお、本研究に係る研究者の利益相反状況ついては、各研究研究機関の利益相反管理体制（各研究機関の利益相反委員会等）の規定に従って管理される。利益相反の開示内容の変更が生じた場合には同委員会等に申告を行う。

**20．知的財産権**

本研究により得られた結果やデータ、知的財産権は、研究代表者（または本研究の研究機関）に帰属する。具体的な取扱いや配分については協議して決定するものとする。

**21．個人情報等の取扱い**

**21.1　個人情報の保護**

本研究の実施に係るデータ類および同意文書等を取扱う際は、研究対象者の個人情報保護に十分配慮する。症例報告書等の記載は識別コードを用いて行う。本研究の結果を公表する際は、研究対象者を特定できる情報を含まないようにする。本研究で収集する情報やデータは、氏名、イニシャル、患者IDなどの個人情報をはずし、新たな符号をつけて加工して個人が識別できないようにする。また、これらの情報の管理は、「14．情報・試料等の収集、保管、廃棄」の項に従い適切に対応するほか、個人情報保護に関して研究機関内に別途規程や手順がある場合はその規程や手順に従い適切に対応する。

**21.2　データの二次利用**

本研究で得られたデータは改めて倫理審査委員会等の承認を経て二次利用（附随研究）される可能性があり、記録の保存に記載された期間を超えて保存される可能性があるが、その場合も研究対象者の個人情報は「21.1 個人情報の保護」の項と同様の方法で保護される。

**22．研究計画書の遵守および研究計画書の変更**

**22.1　研究計画書の遵守**

本研究に携わる研究者は、研究対象者の安全と人権を損なわない限り、研究計画書を遵守して本研究を実施する。

**22.2　研究計画書の変更**

研究代表者は、本研究開始後に研究計画書（説明文書および同意文書を含む）の変更が必要になった場合、倫理審査委員会で変更内容とその理由等について、再度審査を受け、承認を得る。承認後、その変更内容を研究機関の定める手順に従い研究機関の長（病院長）に通知するとともに、他の研究機関の研究責任者にその旨を情報提供する。情報提供を受けた研究責任者は、当該研究機関の手順に従いその変更内容を研究機関の管理者に報告または管理者の実施許可を得る。すべての研究責任者及び担当医師は、倫理審査委員会等の承認または研究機関の長（病院長）の承認を得る前に、変更した研究計画書（説明文書および同意文書）にて研究を実施してはならない。

**23．研究計画書からの逸脱（不適合）の取扱い**

• 研究責任者及び担当医師は、研究代表者の事前の合意及び倫理審査委員会の事前の審査に基づく研究機関の長（病院長）の承認を得る前に、研究計画書からの逸脱あるいは変更を行ってはならない。

• 研究責任者及び担当医師は、緊急回避等やむをえない理由により、倫理審査委員会の事前の承認を得る前に、研究計画書からの逸脱あるいは変更を行うことができる。その際には、研究責任者及び担当医師は、逸脱または変更の内容及び理由ならびに研究計画書等の改訂が必要であればその案を速やかに、倫理審査委員会に提出し、倫理審査委員会及び研究機関の長（病院長）の承認を得るものとする。

• 研究責任者及び担当医師は、研究計画書からの逸脱があった場合は、逸脱事項をその理由とともにすべて記録しなければならない。

• 研究責任者及び担当医師は、不適合のうち特に重大なものが判明した場合においては、速やかに研究機関の研究機関の長（病院長）に報告するとともに、研究代表者に通知する。研究代表者は、倫理審査委員会等に報告し、必要な対応を行う。また、研究代表者は、速やかに他の研究責任者等に情報提供する。

**24．研究機関の長への報告と方法**

研究実施期間中は、研究代表者及び研究責任者は1年に1回、研究の実施状況について、倫理審査委員会及び研究機関の長（病院長）に報告する。

また、以下の項目に該当する内容については、研究代表者及び研究責任者は、速やかに倫理審査委員会及び研究機関の長（病院長）に文書で報告する。

• 研究の倫理的妥当性もしくは科学的合理性を損なう事実、情報または損なうおそれのある情報で研究継続に影響を与えると考えられるもの

• 研究の実施の適正性もしくは研究結果の信頼を損なう事実、情報又は損なう恐れの有る情報

• 重篤な有害事象の発生が認められた場合

• 研究の進捗状況や有害事象の発生状況及び研究が終了（中止含む）した場合

**25．研究の中止**

- 研究代表者が 研究の中止または中断を決定した場合には、速やかに倫理審査委員会と同時に研究機関の長（病院長）にその理由とともに報告＊する。
- 「人を対象とする生命科学・医学系研究に関する倫理指針」に不適合の程度が重大で中止とされた場合には、研究機関の長（病院長）は厚生労働大臣に報告をする。研究対象者は速やかにプロトコール治療を中止する。

＊：東京科学大学「倫理審査申請システム」から報告する。

**26．研究の終了**

- 統計解析報告書の完成を以て、研究の終了とする。研究が終了した場合には、倫理審査委員会と同時に研究機関の長（病院長）に報告するとともに、他の研究機関の研究責任者に情報提供する。情報提供を受けた研究責任者は提供を受けた情報を当該研究機関の管理者に報告する。

※東京科学大学「倫理審査申請システム」を通じて報告する。

- 研究代表者は、研究の終了をjRCT臨床研究等提出・公開システムにもその旨を報告する。

**27．参考資料・引用文献**

1. Nouri A, Tetreault L, Singh A, Karadimas SK, Fehlings MG. Degenerative Cervical Myelopathy: Epidemiology, Genetics, and Pathogenesis. Spine (Phila Pa 1976) 2015;40:E675-93.

2. Nagata K, et al. The prevalence of cervical myelopathy among subjects with narrow cervical spinal canal in a population-based magnetic resonance imaging study: the Wakayama Spine Study. Spine J 2014; 14(12): 2811-2817.

3. 日本整形外科学学会　日本脊椎脊髄病学会監修.頚椎症性脊髄症　診療ガイドライン2020　改訂第3版.P35-37

4. Hirabayashi K, Watanabe K, Wakano K, Suzuki N, Satomi K, Ishii Y. Expansive open-door laminoplasty for cervical spinal stenotic myelopathy. Spine (Phila Pa 1976) 1983;8:693-9.

5. Miyazaki K, Kirita Y. Extensive simultaneous multisegment laminectomy for myelopathy due to the ossification of the posterior longitudinal ligament in the cervical region. Spine (Phila Pa 1976) 1986;11:531-42.

6. 日本整形外科学学会　日本脊椎脊髄病学会監修.頚椎症性脊髄症　診療ガイドライン2020　改訂第3版.P48-54

7. Sakai K, Hirai T, Arai Y, Maehara H, Torigoe I, Inose H, Tomori M, Sakaki K, Yuasa M, Matsukura Y, Oyaizu T, Morishita S, Yoshii T, Okawa A. Laminar Closure in Double-door Laminoplasty for Cervical Spondylotic Myelopathy with Nonkyphotic Alignment. Spine (Phila Pa 1976) 2021;46:999-1006.

8. Fujishiro T, Nakano A, Baba I, Fukumoto S, Nakaya Y, Neo M. Double-door cervical laminoplasty with suture anchors: evaluation of the clinical performance of the constructs. Eur Spine J 2017;26:1121-8.

9. Taniyama T, Hirai T, Yamada T, Yuasa M, Enomoto M, Yoshii T, Kato T, Kawabata S, Inose H, Okawa A. Modified K-line in magnetic resonance imaging predicts insufficient decompression of cervical laminoplasty. Spine (Phila Pa 1976) 2013;38:496-501.

10. Hirai T, Okawa A, Arai Y, Takahashi M, Kawabata S, Kato T, Enomoto M, Tomizawa S, Sakai K, Torigoe I, Shinomiya K. Middle-term results of a prospective comparative study of anterior decompression with fusion and posterior decompression with laminoplasty for the treatment of cervical spondylotic myelopathy. Spine (Phila Pa 1976) 2011;36:1940-7.

11. Yoshiyama T, Fujibayashi S, Otsuki B, Shimizu T, Murata K, Matsuda S. Preoperative and Postoperative Factors Affecting Patient Satisfaction with Double-Door Laminoplasty for Cervical Spondylotic Myelopathy. Spine Surg Relat Res 2023;7:421-7.

12. Hoshi K, Kurokawa T, Nakamura K, et al. Expansive cervical laminoplasties--observations on comparative changes in spinous process lengths following longitudinal laminal divisions using autogenous bone or hydroxyapatite spacers. Spinal Cord 1996;34:725-8.

13. Kaito T, Hosono N, Makino T, et al. Postoperative displacement of hydroxyapatite spacers implanted during double-door laminoplasty. J Neurosurg Spine 2009;10:551-6.

14. Kimura A, Seichi A, Inoue H, Hoshino Y. Long-term results of double-door laminoplasty using hydroxyapatite spacers in patients with compressive cervical myelopathy. Eur Spine J 2011;20:1560-6.

15. Takeoka Y, Yurube T, Maeno K, et al. Improved bone bonding of hydroxyapatite spacers with a high porosity in a quantitative computed tomography-image pixel analysis: A prospective 1-year comparative study of the consecutive cohort undergoing double-door cervical laminoplasty. JOR Spine 2020;3:e1080.

16. Park HG, Zhang HY, Lee SH. Box-shape cervical expansive laminoplasty: clinical and radiological outcomes. Korean J Spine 2014;11:152-6.

17. Oh CH, Ji GY, Hur JW, Choi WS, Shin DA, Lee JB. Preliminary Experiences of the Combined Midline-Splitting French Door Laminoplasty with Polyether Ether Ketone (PEEK) Plate for Cervical Spondylosis and OPLL. Korean J Spine 2015;12:48-54.

18. Wu W, Zhang S, Yan T. Initial clinical experiences of the muscle-preserving double door cervical laminoplasty with adjustable mini plates. Front Surg 2022;9:1049937.

19. Kato S, Oshima Y, Matsubayashi Y, et al. Minimum Clinically Important Difference and Patient Acceptable Symptom State of Japanese Orthopaedic Association Score in Degenerative Cervical Myelopathy Patients. Spine 2019;44:691-7.

20. Okada E, Matsumoto M, Ichihara D, et al. Cross-sectional area of posterior extensor muscles of the cervical spine in asymptomatic subjects: a 10-year longitudinal magnetic resonance imaging study. Eur Spine J 2011;20:1567-73.

21. Ratliff JK, Cooper PR. Cervical laminoplasty: a critical review. J Neurosurg 2003;98:230-8.

22. Takeuchi K, Yokoyama T, Wada KI, et al. A New Grading of Epidural Hematoma or Scar Formation after Posterior Cervical Spine Surgery: Evaluation of Perioperative Related Factors, Distributions, and Clinical Outcomes after Surgery. Spine Surg Relat Res 2019;3:285-94.

23. Mo Z, Li D, Zhang R, et al. Comparison of three fixation modalities for unilateral open-door cervical laminoplasty: a systematic review and network meta-analysis. Neurosurg Rev 2020;43:813-23.
