# Supplementary material for: Clip-plate versus suture-anchor in double-door laminoplasty for degenerative cervical myelopathy: Protocol for a multicenter, non-inferiority, randomized controlled trial
Source: PLoS One. 2026 Apr 24;21(4):e0339103. doi: 10.1371/journal.pone.0339103 (PMC13108724; doi:10.1371/journal.pone.0339103)
Supplement: S3 File — (DOCX) [file pone.0339103.s003.docx]

A Non-Inferiority, Randomized Controlled Trial to Compare the Efficacy of Double-door Laminoplasty Using Mini-plates Versus Suture-Anchors in Degenerative Cervical Myelopathy.

Clinical Research Protocol

I2024-243

Principal Investigator: Toshitaka Yoshii

Department of Orthopedic Surgery, Insitute of Science Tokyo

Address: 1-5-45 Yushima, Bunkyo-ku, Tokyo, 113-8519, Japan

TEL: 03-5803-5279

FAX: 03-5803-0142

e-mail: yoshii.orth@tmd.ac.jp

February 27, 2025 Version 1.0

October 17, 2025 Version 1.1

(Added registration number, revised study period based on jRCT publication date)

**List of Abbreviations**

| ASA | American Society of Anesthesiologists |
| --- | --- |
| CSM | cervical spondylotic myelopathy |
| C-SVA | Cervical sagittal vertical axis |
| CTCAE | Common Terminology Criteria for Adverse Events |
| DCM | degenerative cervical myelopathy |
| FAS | Full analysis set |
| ICER | Incremental cost-effectiveness ratio |
| JOA | Japanese Orthopaedic Association |
| jRCT | Japan Registry of Clinical Trials |
| MCID | Minimal clinically important difference |
| NDI | Neck Disability Index |
| OPLL | Ossification of the Posterior Longitudinal Ligament |
| PPS | Per protocol set |
| QOL | Quality of Life |
| SAS | Safety analysis set |

**目次**

[**１．Overview** 7](#_Toc213878564)

[**２．Clinical Research Implementation Structure** 10](#_Toc213878565)

[**３．Background of the Clinical Study** 11](#_Toc213878566)

[**3.1 About the Target Disease** 11](#_Toc213878567)

[**3.2 Standard Treatment for Target Diseases** 12](#_Toc213878568)

[**3.3 Rationale for Study Design** 14](#_Toc213878569)

[**3.4 Significance of This Study** 14](#_Toc213878570)

[**４．Objectives of the Clinical Study** 15](#_Toc213878571)

[**５．Details Regarding the Clinical Research** 15](#_Toc213878572)

[**5.1　 Primary and Secondary Endpoints** 15](#_Toc213878573)

[**5.2　Clinical Study Design and Outline** 18](#_Toc213878574)

[**5.2.1　Clinical Study Design** 18](#_Toc213878575)

[**5.2.2　Clinical Study Outline** 19](#_Toc213878576)

[**5.3　 Case Registration and Allocation Method** 19](#_Toc213878577)

[**5.4　 Expected Participation Period for Study Subjects** 20](#_Toc213878578)

[**5.5　 Criteria for Terminating the Entire Clinical Study** 20](#_Toc213878579)

[**６．Criteria for Selection and Exclusion of Research Subjects** 20](#_Toc213878580)

[**6.1　 Research Subjects (Target Disease)** 20](#_Toc213878581)

[**6.2　 Inclusion Criteria** 21](#_Toc213878582)

[**6.3　Exclusion Criteria** 21](#_Toc213878583)

[**７．Matters Concerning Treatment for Study Participants** 22](#_Toc213878584)

[**7.1　Protocol Treatment Implementation Procedure** 22](#_Toc213878585)

[**7.1.1　Informed Consent Acquisition** 22](#_Toc213878586)

[**7.1.2　Registration and Randomization** 22](#_Toc213878587)

[**7.1.3　Protocol Treatment** 22](#_Toc213878588)

[**7.1.4　Regarding Treatment Changes** 24](#_Toc213878589)

[**7.1.5　Perioperative Management** 25](#_Toc213878590)

[**7.1.6　Surveillance** 25](#_Toc213878591)

[**7.1.7　Follow-up Treatment** 25](#_Toc213878592)

[**7.2　Observation/Examination Items and Schedule** 26](#_Toc213878593)

[**7.2.1　Observation/Examination Schedule** 26](#_Toc213878594)

[**7.2.2　Observation and Examination Items** 27](#_Toc213878595)

[**7.3　 Concomitant Medications (Therapies) Regulations** 30](#_Toc213878596)

[**7.4** **Instructions for Research Participants** 30](#_Toc213878597)

[**7.5　 Post-Study Measures** 30](#_Toc213878598)

[**7.6　 Individual Research Discontinuation Criteria** 30](#_Toc213878599)

[**８．Evaluation Matters** 31](#_Toc213878600)

[**8.1** **Evaluation Indicators (Endpoints)** 31](#_Toc213878601)

[**8.1.1　 Primary endpoint** 31](#_Toc213878602)

[**8.1.2　Secondary endpoint** 32](#_Toc213878603)

[**8.2** **Evaluation and Recording of Assessment Indicators** 32](#_Toc213878604)

[**8.3** **Methods and Timing for Analyzing Evaluation Indicators** 42](#_Toc213878605)

[**９．Matters Concerning Recording, Reporting, etc. of Adverse Events, Surgical Complications, and Malfunctions** 42](#_Toc213878606)

[**9.1** **Methods for Recording, Reporting, and Analyzing Adverse Events, Surgical Complications, and Malfunctions** 42](#_Toc213878607)

[**9.2** **Anticipated Adverse Events** 44](#_Toc213878608)

[**9.3　Response to Adverse Events, Surgical Complications, and Malfunctions** 45](#_Toc213878609)

[**9.3.1　Response to Adverse Events, Surgical Complications, and Malfunctions** 45](#_Toc213878610)

[**9.3.2　Response to Serious Adverse Events, Surgical Complications, and Malfunctions** 45](#_Toc213878611)

[**9.3.3　Response to Unforeseen Serious Adverse Events, Surgical Complications, or Malfunctions** 46](#_Toc213878612)

[**9.4　Observation of Research Subjects Following Adverse Events, Surgical Complications, or Malfunctions** 46](#_Toc213878613)

[**10．Matters Concerning Statistical Analysis** 46](#_Toc213878614)

[**10.1　Analysis Population** 46](#_Toc213878615)

[**10.1.1　Full Analysis Set (FAS)** 46](#_Toc213878616)

[**10.1.2　Safety Analysis Set (SAS)** 47](#_Toc213878617)

[**10.2　Target Enrollment Numbers and Rationale** 47](#_Toc213878618)

[**10.3　Case Handling** 47](#_Toc213878619)

[**10.4　Data Handling** 48](#_Toc213878620)

[**10.5　Statistical Analysis Items and Analysis Plan** 48](#_Toc213878621)

[**10.5.1　Summary of Subject Background** 48](#_Toc213878622)

[**10.5.2　Analysis of Primary Endpoints** 48](#_Toc213878623)

[**10.5.3　Secondary Outcome Analysis** 49](#_Toc213878624)

[**10.5.4　Subgroup Analysis** 51](#_Toc213878625)

[**10.6　Interim Analysis** 51](#_Toc213878626)

[**10.7　Primary Analysis** 51](#_Toc213878627)

[**10.8　Final Analysis** 51](#_Toc213878628)

[**11．Matters Concerning Access to Original Data and Related Materials** 51](#_Toc213878629)

[**12．Matters Concerning Quality Control and Quality Assurance** 52](#_Toc213878630)

[**12.1　Monitoring** 52](#_Toc213878631)

[**12.2　Audit** 52](#_Toc213878632)

[**13．Ethical Considerations** 53](#_Toc213878633)

[**13.1　Compliance with Laws and Regulations** 53](#_Toc213878634)

[**13.2　Expected Benefits, Burdens, and Harms** 53](#_Toc213878635)

[**13.3　Handling of Research Findings and Incidental Findings Related to Genetic Characteristics of Research Subjects** 54](#_Toc213878636)

[**14．Matters Concerning Collection, Storage, and Disposal of Information and Samples** 54](#_Toc213878637)

[**14.1　Collection, Storage, and Disposal of Information** 54](#_Toc213878638)

[**14.2** **Sample Collection, Storage, and Disposal** 55](#_Toc213878639)

[**15．Matters Concerning Monetary Payments and Compensation** 55](#_Toc213878640)

[**15.1** **Monetary Payments (Research Subject Expenses)** 55](#_Toc213878641)

[**15.2** **Compensation** 55](#_Toc213878642)

[**16．Information Disclosure** 55](#_Toc213878643)

[**17．Implementation Period** 55](#_Toc213878644)

[**18．Explanation and Consent for Research Participants** 56](#_Toc213878645)

[**19．Matters Concerning Conflicts of Interest** 57](#_Toc213878646)

[**20．Intellectual Property Rights** 58](#_Toc213878647)

[**21．Handling of Personal Information** 58](#_Toc213878648)

[**21.1** **Protection of Personal Information** 58](#_Toc213878649)

[**21.2** **Secondary Use of Data** 58](#_Toc213878650)

[**22．Compliance with the Research Protocol and Protocol Amendments** 58](#_Toc213878651)

[**22.1** **Compliance with the Research Protocol** 58](#_Toc213878652)

[**22.2** **Changes to the Research Protocol** 58](#_Toc213878653)

[**23．Handling of Deviations (Non-Compliance) from the Research Protocol** 59](#_Toc213878654)

[**24．Reporting to the Head of the Research Institution and Method** 59](#_Toc213878655)

[**25．Discontinuation of Research** 60](#_Toc213878656)

[**26．Research Termination** 60](#_Toc213878657)

[**27．References** 60](#_Toc213878658)

**１．Overview**

| **Study Title** | A Non-Inferiority, Randomized Controlled Trial to Compare the Efficacy of Double-door Laminoplasty Using Mini-plates Versus Suture-Anchors in Degenerative Spondylotic Myelopathy. |
| --- | --- |
| **Study Purpose** | To demonstrate that the conventional, less expensive suture anchor method (suture anchor technique) does not have inferior efficacy or safety compared to the plate method (mini-plate), which is becoming one of the standard surgical approaches in bilateral cervical laminoplasty (Suture-anchor), which uses inexpensive suture anchors and has been traditionally performed. |
| **Study Design** | Multicenter, open-label randomized controlled trial |
| **Nature of Study** | Verification research |
| **Treatment for Study Participants** | <Control Group>  Bilateral laminoplasty using mini-plates  (Device used: LAMINAclip2, Olympus Terumo Biomaterial Co., Ltd.)  <Experimental Treatment Group>  Bilateral laminectomy using suture-anchors  (Device used: LAMIFIX, Olympus-Terumo Biomaterial Co., Ltd.) |
| **Subjects** | Patients with degenerative spondylotic myelopathy (DCM) requiring cervical laminectomy surgery |
| **Inclusion Criteria** | Patients meeting all of the following criteria will be enrolled in this clinical study.  1) Patients with spinal cord symptoms due to DCM (cervical spondylotic myelopathy [CSM] or ossification of the posterior longitudinal ligament [OPLL]) scheduled for cervical laminoplasty  2) Patients with spinal cord stenosis at the C3-7 level on MRI or CT  3) Patients aged 20 years or older but under 90 years at the time of consent acquisition  4) Patients who provide written informed consent to participate in this clinical study. |
| **Exclusion Criteria** | Patients meeting any of the following criteria will not be included in this clinical study.  1) Patients for whom cervical laminoplasty is an inappropriate surgical procedure (e.g., patients with significant cervical kyphosis, anterior spinal cord compression, or cervical spinal instability on MRI or CT).  2) Patients with concomitant foraminal stenosis (requiring concomitant posterior foraminal enlargement)  3) Patients with spinal infection  4) Patients with spinal tumors (including metastatic tumors)  5) Patients with traumatic spinal cord injury)  6) Patients with a history of cervical spine surgery  7) Patients undergoing maintenance dialysis  8) Patients with cerebral palsy  9) Patients with Parkinson's disease  10) Pregnant women or women who may be pregnant  11) Inappropriate case judged by doctor in charge |
| **Evaluation Items** | **Primary Endpoint**  **Recovery rate in cervical JOA score before and after surgery**  Recovery rate in cervical JOA score = (Postoperative score - Preoperative score) / (17 - Preoperative score) × 100 (%)  The primary analysis time point is postoperative 1 year. Results at 2 years post-surgery will also be presented as a final analysis. The same applies to secondary endpoints 3) to 13).  *JOA: Japanese Orthopaedic Association  **Secondary Endpoints**  1) Operative time  2) Estimated blood loss  3) Proportion achieving the minimal clinically important difference (MCID) in cervical JOA score recovery rate at postoperative 1 and 2 years  4) Health-related QOL (EQ-5D) at postoperative 1 and 2 years  5) Severity of neck pain, upper limb pain, and upper limb numbness (VAS) at postoperative 1 and 2 years  6) Neck disability index (NDI) at postoperative 1 and 2 years compared to preoperative  7) Retention rate of enlarged lamina at postoperative 1 and 2 years (Retention rate)  8) Percentage of hinge fractures at postoperative 1 and 2 years  9) Percentage of bone union of gutter at postoperative 1 and 2 years  10) Sagittal alignment of cervical spine at postoperative 1 and 2 years (C-SVA, C2-7 angle, T1 slope)  11) Paraspinal muscle cross-sectional area (C4/5 level) at postoperative 1 and 2 years  12) Dural sac cross-sectional area (C3/4, C4/5, C5/6, C6/7 levels) at postoperative 1 and 2 years  13) Grading of the mass posterior to the dural sac at postoperative 1 and 2 years  14) Incremental Cost-Effectiveness Ratio (ICER)  15) Surgical complication incidence |
| **Protocol Treatment** | 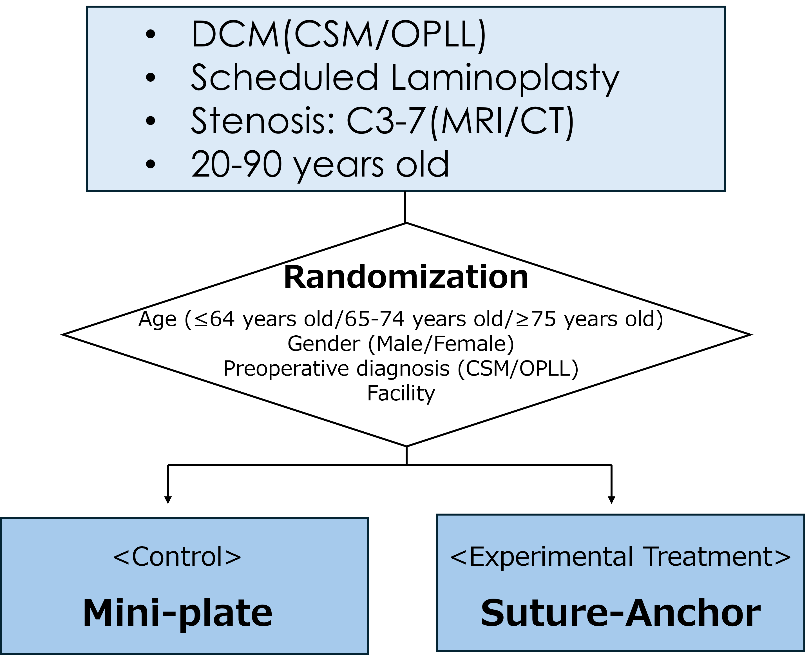  The principal investigator (or designated surgeon) will perform surgery (mini-plate or suture-anchor) according to the allocation results. Surgery will be performed by an orthopedic surgeon experienced in cervical laminoplasty. The surgical method and pre- and postoperative evaluation schedule are as follows.  **【Surgical Method】**  Under general anesthesia, a midline longitudinal skin incision is made from C2 to C7 in the prone position. Dissection is performed up to the medial facet joints. A double-door laminoplasty is performed on the laminae of C3-6, enlarging 2-4 laminae.  Dome-shaped laminectomy of C2 and C7 is added as needed.  ＜Control Group＞ Mini-Plate  Screws are placed in the expanded laminae at C3-6, and a plate is applied. Screw and plate sizes are determined according to the lamina shape in each case.  ＜Experimental Treatment Group＞ Suture-Anchor  An anchor is placed in the lateral mass at any level from C3-6. Sutures are applied to the ligamentum flavum and sutured to maintain the expanded position.  **[Pre- and Postoperative Evaluation Schedule]**  1) Pre-operative: X-ray, CT, MRI, clinical evaluation  2) Postoperative 1 week: CT  3) Postoperative 1 year: X-ray, CT, MRI, clinical evaluation, direct medical costs  4) Postoperative 2 years: X-ray, CT, MRI, clinical evaluation, direct medical costs |
| **Target Study Population** | 216cases |
| **Research Period** | Total Research Period: October 17, 2025 (jRCT Publication Date) to March 31, 2031 (5.5 years)  (Planned Registration Period: 3 years, Follow-up Period: 2 years after registration completion, Analysis Period: 0.5 years) |
| **Number of Research Facilities** | 3 facilities (Institute of Science Tokyo Hospital, Saiseikai Kawaguchi General Hospital, Saku Medical Center) |

**２．Clinical Research Implementation Structure**

| Role | Name | Title | Affiliation/Department, etc.* | Contact |
| --- | --- | --- | --- | --- |
| Principal Investigator | Toshitaka Yoshii | Professor | Institute of Science Tokyo Dept. of Orthopedic Surg. | 03-5803-5279 |
| Coordinating Manager  (Research Office) | Kentaro Yamada | Assistant Professor | Institute of Science Tokyo Dept. of Orthopedic Surg. | 03-5803-5279 |
| Data Management Officer | Yasunori Sakamaki | URA | Institute of Science Tokyo Hospital. Health Science R&D Center | 03-5803-5465 |
| Monitoring Officer | Koetsu Kuwamoto | URA | Institute of Science Tokyo Hospital. Health Science R&D Center | 03-5803-5465 |
| Statistical Analysis Lead | Akihiro Hirakawa | Professor | Institute of Science Tokyo Dept. of Clinical Statistics | 03-5803-5150 |
| Statistical Analysis Staff | Hiroyuki Sato | Lecturer | Institute of Science Tokyo Dept. of Clinical Statistics | 03-5803-5150 |
|  | Ryoichi Hanazawa | Specially Appointed Assistant Professor |  |  |
|  | Ryo Kitabayashi | Specially Appointed Assistant Professor |  |  |

<Research Implementation Medical Institutions>

①　Institute of Science Tokyo, Dept. of Orthopedic Surg.

Address:〒113-8510　 1-5-45 Yushima, Bunkyo-ku, Tokyo

Phone：03-5803-5279

Principal Investigator: Toshitaka Yoshii (Professor)

②　Saiseikai Kawaguchi General Hospital, Dept. of Orthopedic Surg.

Address:〒332-8558　 5-11-5 Nishikawaguchi, Kawaguchi-shi, Saitama

Phone：0570-08-1551

Principal Investigator: Kenichiro Sakai (Department Head)

③　Saku Medical Center,　Dept. of Orthopedic Surg.

Address:〒385-0051 3400-28 Nakagomi, Saku City, Nagano

Phone：[0267-62-8181](https://www.google.com/search?q=%E4%BD%90%E4%B9%85%E5%8C%BB%E7%99%82%E3%82%BB%E3%83%B3%E3%82%BF%E3%83%BC&sca_esv=4518de3dcf4930bc&sxsrf=ADLYWIImcjXcC8O9TVzg3vByYSjiJuhtLw%3A1737425724450&source=hp&ei=PAOPZ-e_GO_b2roPk5TS8Q0&iflsig=AL9hbdgAAAAAZ48RTJbBZBIwAAg3MG4chxHkWT47jpL9&ved=0ahUKEwjnzYGZ34WLAxXvrVYBHROKNN4Q4dUDCBk&uact=5&oq=%E4%BD%90%E4%B9%85%E5%8C%BB%E7%99%82%E3%82%BB%E3%83%B3%E3%82%BF%E3%83%BC&gs_lp=Egdnd3Mtd2l6IhjkvZDkuYXljLvnmYLjgrvjg7Pjgr_jg7wyBRAAGIAEMgUQABiABDIFEAAYgAQyBRAAGIAEMgUQABiABDIFEAAYgAQyBRAAGIAEMgUQABiABDIFEAAYgAQyBRAAGIAESMMxUNwBWIAucAh4AJABAJgBwAGgAb0hqgEEMC4yNbgBA8gBAPgBAZgCHaAC4ByoAgvCAgYQswEYhQTCAgoQABgDGOoCGI8BwgIHEAAYgAQYBMICEBAAGIAEGLEDGIMBGAQYigXCAgsQABiABBixAxiDAcICDRAAGIAEGLEDGIMBGATCAgoQABiABBixAxgEwgIMEAAYgAQYBBhGGPkBwgIPEAAYgAQYsQMYBBhGGPkBwgIIEAAYgAQYsQPCAg4QABiABBixAxiDARiKBcICBhAAGAQYHpgDCfEFSys5ZuKbjT-SBwQ4LjIxoAeJZg&sclient=gws-wiz)

Principal Investigator: Kazuyuki Fukushima (Director)

**３．Background of the Clinical Study**

## **3.1 About the Target Disease**

Degenerative spondylotic myelopathy (DCM) is a disease concept encompassing both Cervical Spondylotic Myelopathy (CSM), which involves compressive spinal cord lesions due to progressive changes without ligament ossification, and Ossification of the posterior longitudinal ligament (OPLL) (Figure 1).


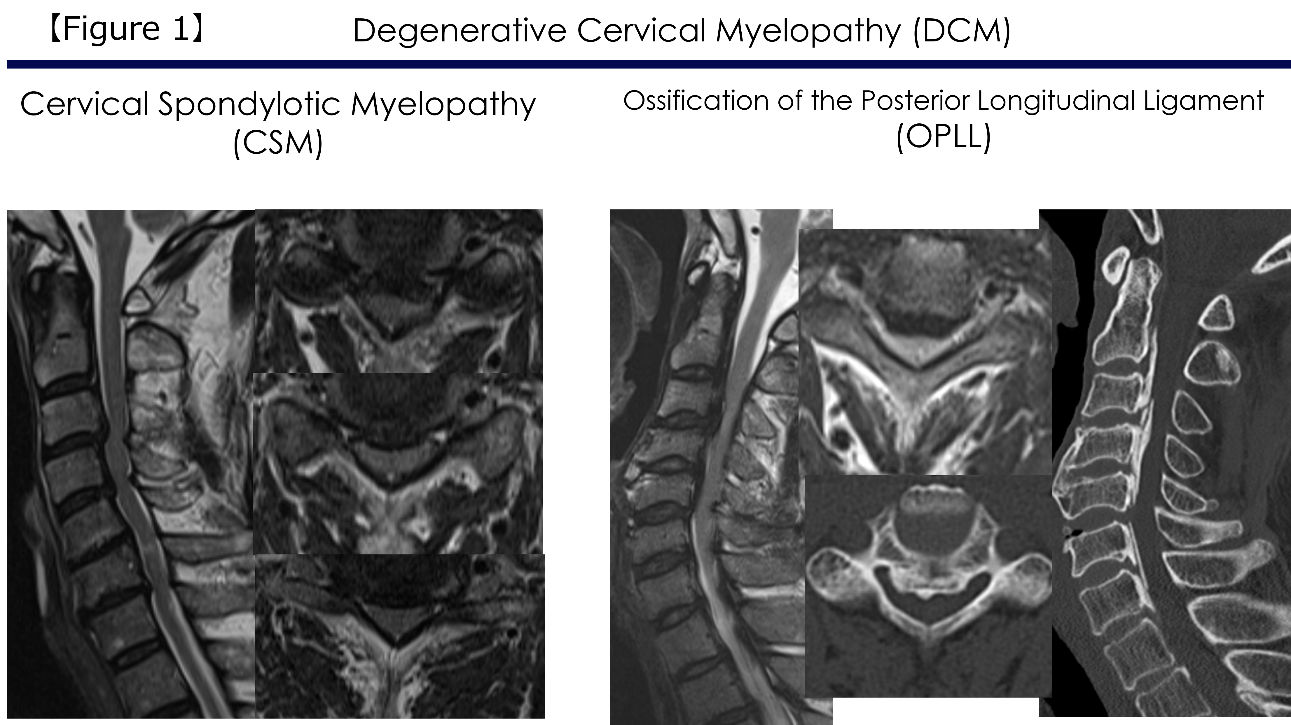


The prevalence of DCM has been reported to vary between ethnic groups. In the United States, the prevalence is reported to be 41-605 per million people (0.004-0.06%)^1)^. In contrast, in Japan, a health screening of 959 residents in Wakayama Prefecture reported a DCM prevalence of 10.1%^2)^, indicating it is a disease with a high prevalence in East Asians. Compression of the cervical spinal cord causes sensory disturbances (numbness in the upper and lower limbs), hand clumsiness, and spastic gait. As the condition progresses, it leads to motor paralysis, sensory disturbances, and bladder/bowel dysfunction. The effectiveness of conservative treatment is limited, and surgical intervention becomes the first choice when symptoms advance³⁾.

## **3.2 Standard Treatment for Target Diseases**

Surgical treatment for DCM has developed primarily in Japan, as mentioned above, due to its high prevalence among Asians. Extensive laminectomy was commonly performed in the 1970s and 1980s. However, issues such as postoperative progression of cervical kyphosis and recurrence of spinal cord symptoms due to posterior dural scarring after laminectomy were noted. Consequently, in the late 1980s, Japanese surgeons developed cervical laminoplasty as a posterior approach technique. Two techniques for cervical laminoplasty have been proposed: the open-door technique^4)^ and the double-door technique^5)^ (Figure 2).


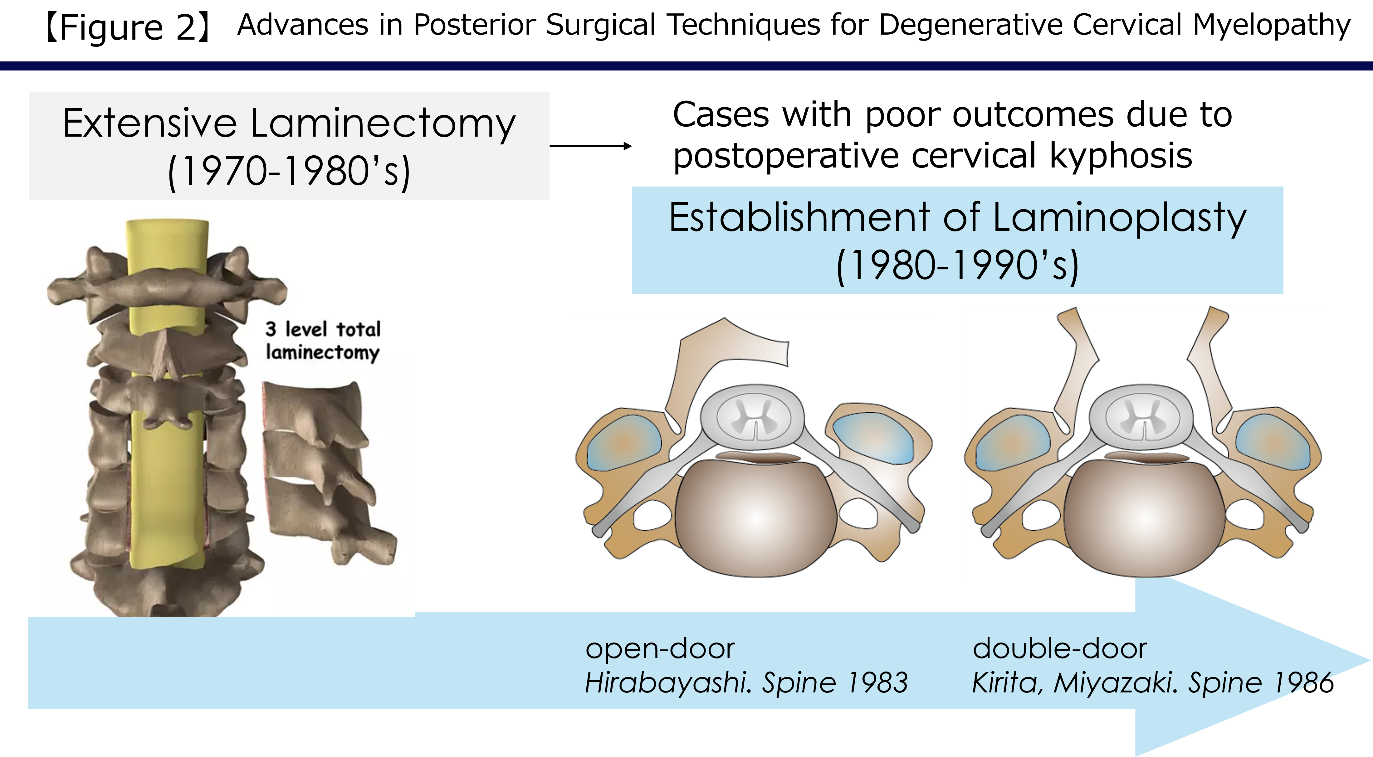


Reports indicate no difference in postoperative outcomes between the unilateral and bilateral approaches, establishing laminaoplasty as the standard treatment for DCM without kyphotic deformity or anterior compression^6)^.

Problems associated with the bilateral laminoplasty approach include reported worsening of myelopathy due to reclosure of the enlarged lamina and cervical kyphosis^7,8)^. Therefore, to prevent reclosure of the enlarged lamina, the suture-anchor method (Fig. 3 left)^9-11)^ and the lamina spacer method (Fig. 3 center)^12-15)^ were developed. In recent years, the mini-plate method (Fig. 3 right)^16-18)^ has become widespread.


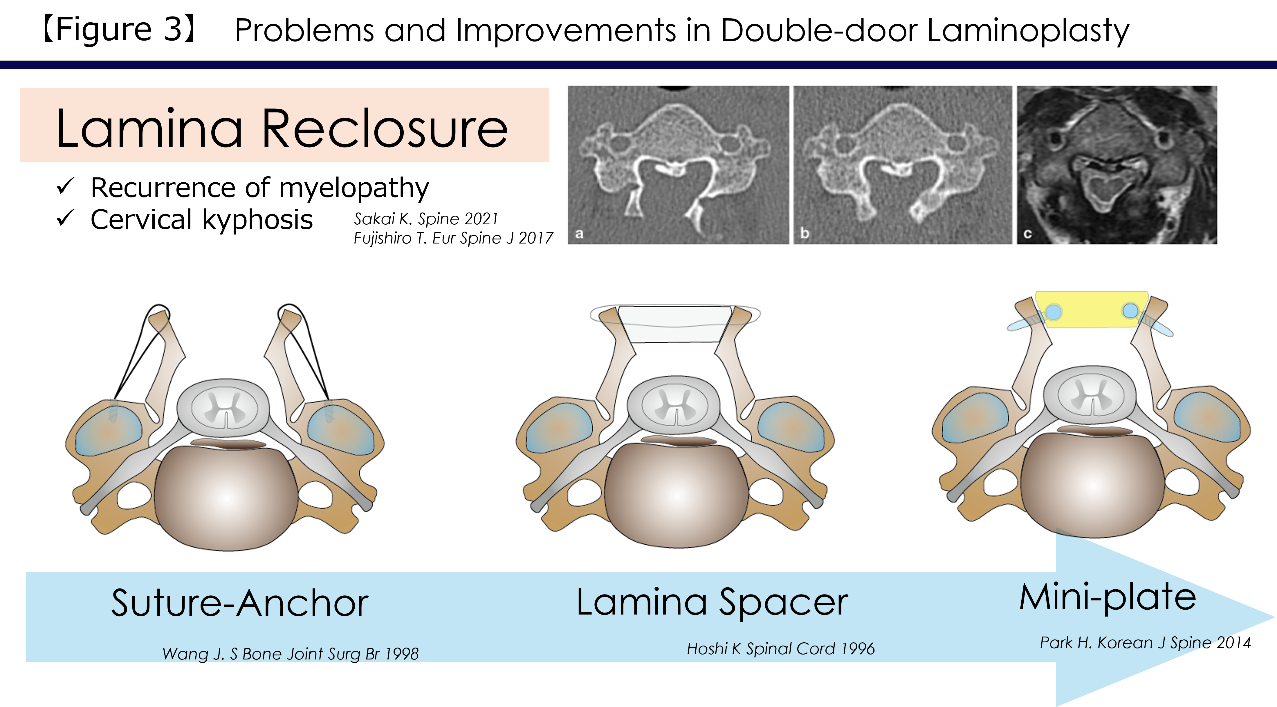


|  | Suture-Anchor | Laminar Spacer | Mini-plate |
| --- | --- | --- | --- |
| reclosure of enlarged laminae | 1.8-5.5% | 2.9-21.3%  (Spacer dislodgement rate) | 3.4-13.3%  (Screw dislodgement rate) |
| JOA score recovery rate | 42.4-59.8% | 43.2-52.3％ | 47.1-83.4% |
| Technical complexity | Simple | Complex | Simple |
| Cost (material fee) | Low  138,000yen／2 laminae | low  120,800 yen／4 laminae | High  506,700 yen／３laminae |
| Surgical Fee (Medical Fee) | 727,800 yen／5 laminae | | |

　　　※JOA score：Japanese Orthopaedic Association score

The suture anchor method has also reported favorable surgical outcomes with JOA score recovery rates of 42.4-59.8%^9-11)^. On the other hand, the plate method has been reported to offer the advantages of a simpler surgical procedure and superior fixation strength^16-18)^. However, no high-quality randomized controlled trials have been conducted to determine whether the plate method yields superior surgical outcomes compared to the conventional suture anchor method, and no conclusion has been reached.

Furthermore, even with the plate method, reclosure of the enlarged lamina due to screw loosening has been reported in 3.4-13.3% of cases. No comparative studies including safety aspects have been reported to date to determine whether the plate method is safer than the suture anchor method, which has a lamina reclosure rate of 1.8-5.5%.

While the surgical fees for the three methods are the same, material costs differ significantly. Under Japan's insurance reimbursement system, the plate method costs ¥506,700 for use on three laminae, which is higher than the ¥138,000 for the suture anchor method used on two laminae. To date, no cost-effectiveness analysis has been conducted, and the benefits of using the more expensive plate method remain unclear.

## **3.3 Rationale for Study Design**

Therefore, this randomized non-inferiority trial aims to demonstrate that the conventional, less expensive suture anchor method is non-inferior in efficacy and safety to the plate method, which is becoming one of the current standard procedures.

The primary endpoint, representing the most relevant measure of efficacy for this surgery, is the JOA score. The pre- and post-operative recovery rate in the JOA score^4)^ is commonly used as an indicator of therapeutic effect in surgical treatment. Therefore, the primary endpoint is defined as the recovery rate in the cervical JOA score between pre- and post-operative measurements. The cervical JOA score is the most widely used international indicator for evaluating treatment outcomes in compressive myelopathy.

Cervical JOA Score Recovery Rate = (Postoperative Score - Preoperative Score) / (17 - Preoperative Score) × 100 (%)

*JOA: Japanese Orthopaedic Association

The primary analysis time point was set at 1 year postoperatively, based on the rationale that recovery rates do not change significantly beyond 1 year postoperatively¹⁴⁾. Results at 2 years postoperatively were also evaluated as a final analysis.

## **3.4 Significance of This Study**

　　To date, no high-quality randomized controlled trials have been reported comparing cervical laminoplasty techniques and materials. The anticipated outcome of this study is to demonstrate that the traditional suture anchor method is non-inferior in efficacy and safety to the plate method, which is becoming the current standard of care, and that it is also less expensive. Cost-effectiveness analysis will also be performed as a secondary endpoint. The results of this study will provide important evidence for discussing the necessity of the plate method, which requires expensive materials, and are expected to contribute to future surgical technique selection. provide crucial evidence for discussing the necessity of the plate method, which requires expensive materials, and contribute to future surgical method selection.

# **４．****Objectives of the Clinical Study**

To verify, through a randomized controlled trial, that the conventional, low-cost suture-anchor method does not show inferior efficacy or safety compared to the mini-plate method—a double-door laminectomy using plates, which is becoming one of the current standard procedures—in patients with DCM requiring cervical laminoplasty.

**５．Details Regarding the Clinical Research**

**5.1　 Primary and Secondary Endpoints**

**１）Primary Endpoint**

Recovery rate in cervical JOA score before and after surgery

[Rationale]

The cervical JOA score is the most widely used international indicator for evaluating treatment outcomes in DCM. Since the recovery rate in the JOA score before and after surgery^4)^ is commonly used to evaluate the therapeutic effect of surgical treatment, the recovery rate in the cervical JOA score before and after surgery was set as the primary endpoint. The main analysis time point was set at postoperative 1 year, based on the rationale that the recovery rate does not change significantly after 1 year post-surgery^14)^. Results at postoperative 2 years will also be evaluated as a final analysis.

**２）Secondary Endpoints**

1) Operative time

2) Estimated blood loss

3) Proportion achieving the minimal clinically important difference (MCID) in cervical JOA score recovery rate at postoperative 1 and 2 years

4) Health-related QOL (EQ-5D) at postoperative 1 and 2 years

5) Severity of neck pain, upper limb pain, and upper limb numbness (VAS) at postoperative 1 and 2 years

6) Neck disability index (NDI) at postoperative 1 and 2 years compared to preoperative

7) Retention rate of enlarged lamina at postoperative 1 and 2 years (Retention rate)

8) Percentage of hinge fractures at postoperative 1 and 2 years

9) Percentage of bone union of gutter at postoperative 1 and 2 years

10) Sagittal alignment of cervical spine at postoperative 1 and 2 years (C-SVA, C2-7 angle, T1 slope)

11) Paraspinal muscle cross-sectional area (C4/5 level) at postoperative 1 and 2 years

12) Dural sac cross-sectional area (C3/4, C4/5, C5/6, C6/7 levels) at postoperative 1 and 2 years

13) Grading of the mass posterior to the dural sac at postoperative 1 and 2 years

14) Incremental Cost-Effectiveness Ratio (ICER)

15) Surgical complication incidence

[Rationale]

(1)(2) The Clinical Question (CQ) that led to this study's design is: “While the plate method is a simple surgical technique, it is expensive. Could the less expensive conventional method (suture-anchor) be equally effective and safe compared to the mini-plate?” Since the simplicity of a surgical technique can be measured by operating time and blood loss, these were set as secondary endpoints as direct measures to answer the CQ.

(3) In recent years, evaluating treatment effectiveness has commonly involved not only simple comparisons using patient-reported outcome measures but also assessment via the MCID (Minimal Clinically Important Difference). If treatment yields a change exceeding the MCID threshold, the treatment is considered effective. The MCID for recovery in the JOA score in compressive myelopathy has been reported as 52.8%^19)^. As secondary endpoints, comparisons between treatment groups at 1 and 2 years will be made not only for the direct intergroup comparison of recovery rates but also for the proportion achieving the MCID.

(4) ～(6)　The JOA score is a physician-led assessment of spinal cord symptoms originating from the cervical spine. Patient-reported outcomes such as EQ-5D, VAS, and NDI were set as secondary endpoints. Furthermore, EQ-5D will also be used as the utility value when calculating the incremental cost-effectiveness ratio.

(7) For safety assessment, the retention rate^7)^ (Figure 4), an objective indicator for enlarged lamina reclosure, was set as a secondary endpoint.


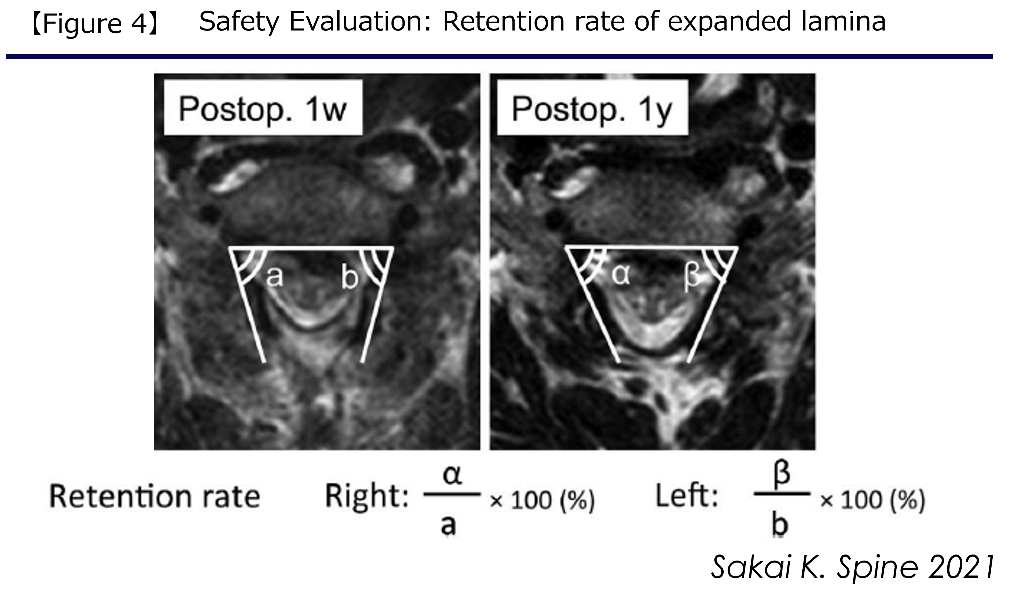


(8) As a safety endpoint, the proportion of hinge fractures in enlarged laminar segments on CT^8)^ was set as a secondary endpoint.

(9) As an endpoint for surgical goal achievement, the proportion of hinge fractures showing bone union on CT was set as a secondary endpoint.

(10) Postoperative cervical kyphotic deformity is a morphological change that leads to recurrence of spinal cord symptoms and poor outcomes in cervical laminoplasty^7-9)^. As indicators of kyphotic deformity, the C2-7 lordotic angle and C-SVA were set as secondary endpoints, along with the C7 slope as a factor influencing these (Figure 5).


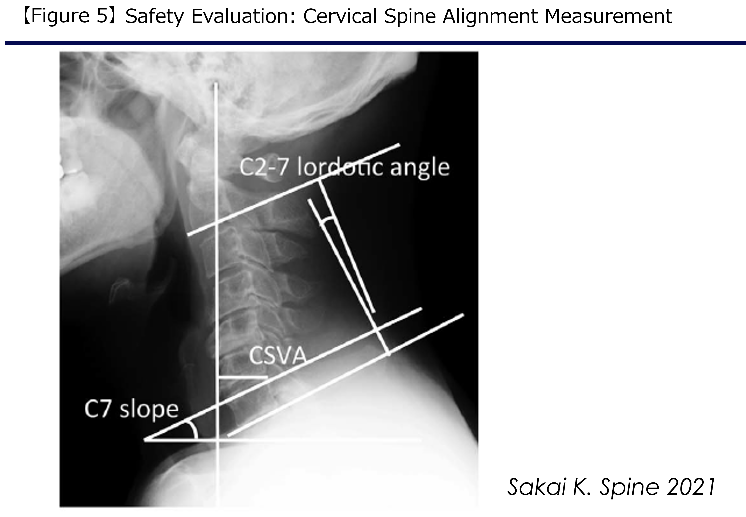


(11) Reduction in the cross-sectional area of the paraspinal muscles (Fig. 6), which are cervical extensors, has been reported as a factor influencing occipital pain^20)^ and as a factor involved in post-laminoplasty reclosure^7)^. Therefore, the postoperative paraspinal muscle area at the C4-5 level was set as a secondary endpoint.


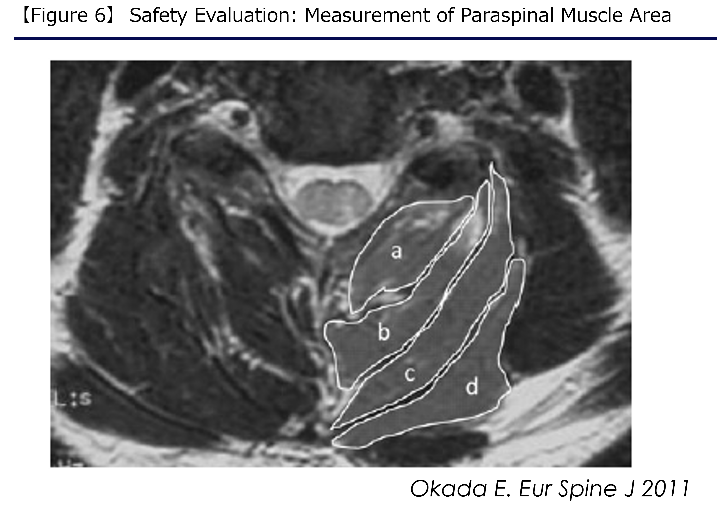


(12)(13) Post-laminectomy membrane, caused by posterior dural scarring after laminectomy, is considered a factor in spinal cord re-compression^21)^. However, its incidence rate across different laminoplasty techniques remains unclear. Postoperative dural canal area and the Grading of the mass posterior to the dural sac ^22)^ (Figure 7) were set as secondary endpoints to evaluate spinal cord re-compression.


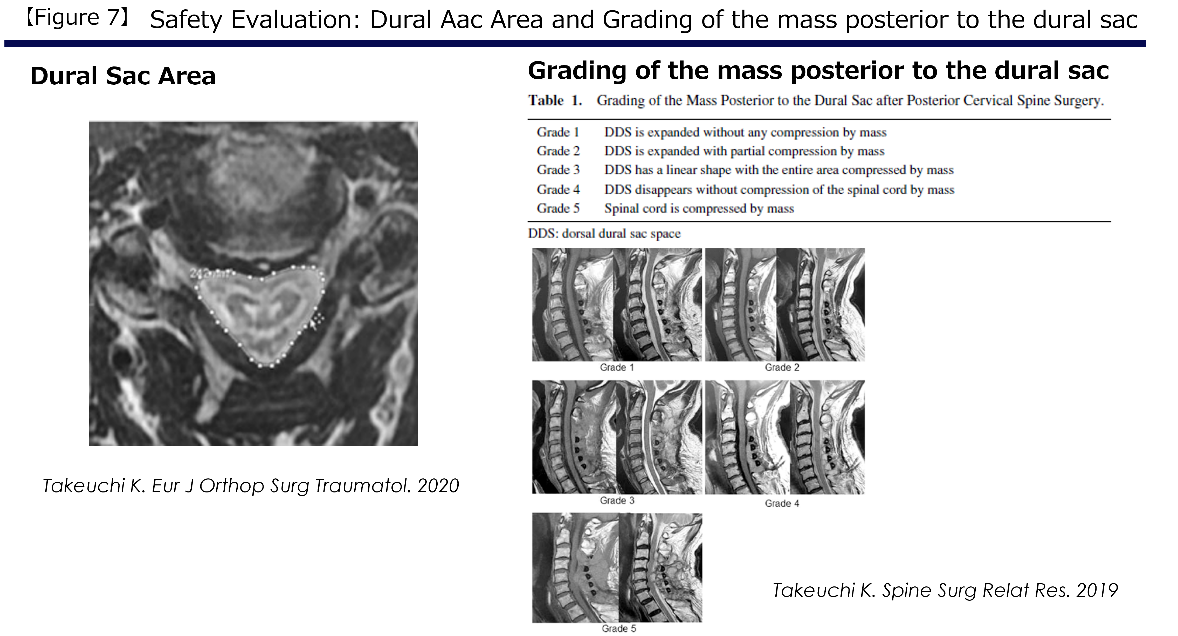


(14) A key objective of this study is to demonstrate that the experimental treatment (suture- anchor) is non-inferior in efficacy and safety to the control treatment (mini-plate), while being less expensive and offering superior cost-effectiveness. As an indicator for comparing the cost-effectiveness between the experimental and control groups, the commonly used measure, ICER, was set as a secondary endpoint.

(15) The incidence of surgical complications, including reoperations, is a commonly used indicator for evaluating the clinical utility of surgical treatment. Therefore, it was set as a secondary endpoint.

## **5.2　Clinical Study Design and Outline**

**5.2.1　Clinical Study Design**

1) Nature of the Study: Verification research

2) Randomization: Randomized Controlled

3) Blinding: Non-blinded

4) Control: Treatment control

5) Allocation: Parallel group comparisons

**5.2.2　Clinical Study Outline**


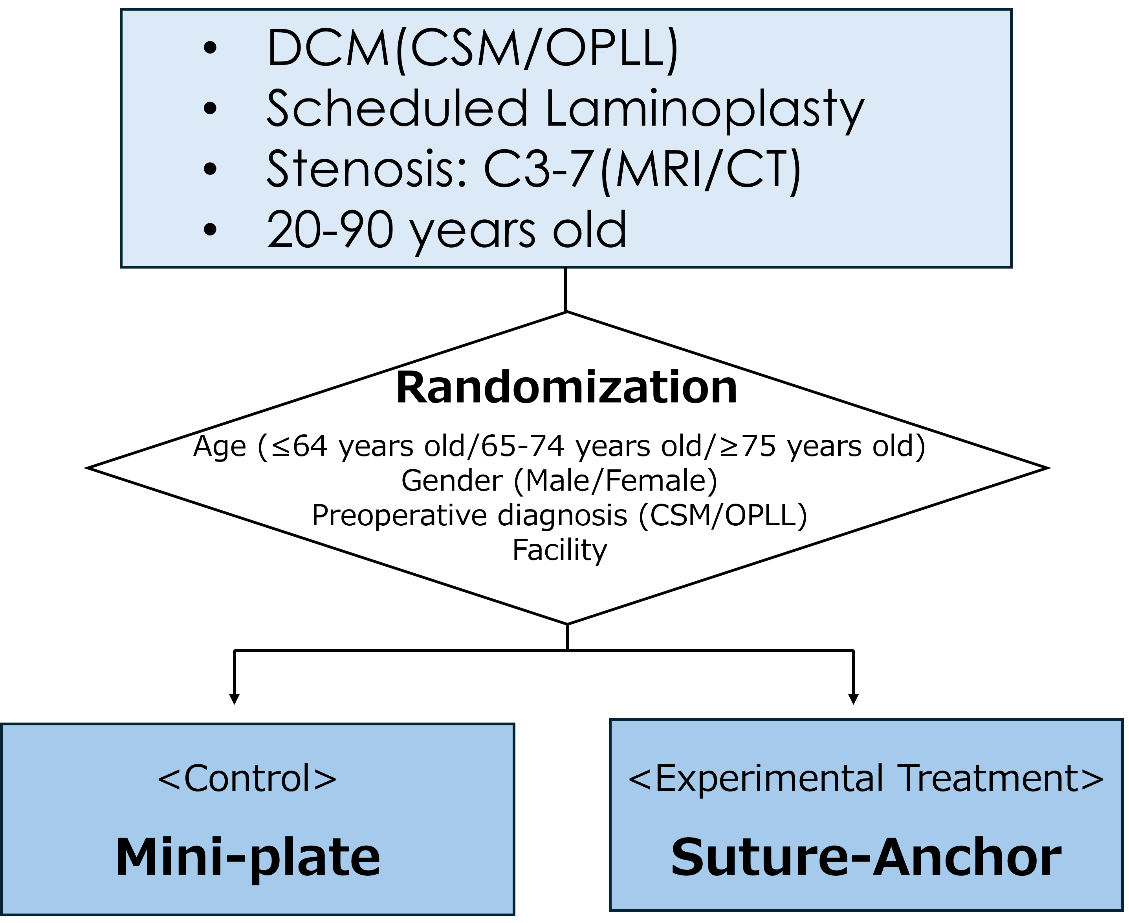


**5.3　 Case Registration and Allocation Method**

1) Case Registration Method

The principal (or co-principal) investigator confirms that patients who have provided written informed consent meet all inclusion criteria and do not meet any exclusion criteria. Subsequently, personnel involved in this clinical study enter patient information into the EDC system to register and allocate the study subjects.

The subject's registration number and the method of laminectomy stabilization (plate method or suture anchor method) will be displayed in the EDC system and also recorded in a printable/downloadable PDF file. Study personnel shall confirm the subject's registration number and the assigned laminectomy stabilization method (plate method or suture anchor method), and record this information in the subject roster or print and retain the PDF containing the assignment results.

2) Allocation Method

- Allocation Method: Minimization Method
- Allocation Adjustment Factors: ① Facility, ② Age, ③ Gender, ④ Preoperative Diagnosis

[Rationale]

① Facility (Institute of Science Tokyo Hospital / Saiseikai Kawaguchi General Hospital / Saku Medical Center)

To account for potential differences between facilities in patient background, short-term outcomes, etc.

② Age (64 years or younger / 65–74 years / 75 years or older)

③ Gender (Male / Female)

Age and gender are common prognostic factors in treatment outcomes for DCM patients.

④ Preoperative Diagnosis (Cervical Spondylotic Myelopathy (CSM) / Ossification of the Posterior Longitudinal Ligament (OPLL))

Treatment prognosis has been reported to differ depending on the pathology.

**5.4　 Expected Participation Period for Study Subjects**

Expected participation period: Approximately 2 years

(Period from registration to surgery: Maximum 90 days; Follow-up period: 2 years post-surgery)

After the expected participation period ends, if any adverse events suspected to be related to the study are identified, follow-up will continue until the principal (or sub-principal) investigator determines the study subject's safety is assured. Note that this follow-up period shall not be included in the study period.

**5.5　 Criteria for Terminating the Entire Clinical Study**

The Principal Investigator shall consider whether to continue the study if any of the following occur:

1) If important information affecting the conduct or continuation of the study, or other information critical for proper study conduct, becomes known.

2) If subject recruitment proves difficult and it is judged that the planned number of cases cannot be achieved.

3) When the study objectives are achieved prior to reaching the planned number of cases or the planned period, as determined by interim analysis, etc.

4) When the Ethics Review Committee or similar body issues instructions for amendments to the study protocol, etc., and acceptance of these amendments is difficult.

5) When the Ethics Review Committee or similar body decides to terminate the study.

6) When a serious or persistent violation of the Clinical Research Act, its Enforcement Regulations, or this study protocol occurs.

**６．Criteria for Selection and Exclusion of Research Subjects**

**6.1　 Research Subjects (Target Disease)**

Patients with degenerative cervical myelopathy (DCM) requiring cervical laminoplasty.

**6.2　 Inclusion Criteria**

Patients meeting all of the following criteria will be enrolled in this clinical research.

1) Patients with spinal cord symptoms due to DCM (cervical spondylotic myelopathy [CSM] or ossification of the posterior longitudinal ligament [OPLL]) scheduled for cervical laminoplasty

2) Patients with spinal cord stenosis at the C3-7 level on MRI or CT

3) Patients aged 20 years or older but under 90 years at the time of consent acquisition

4) Patients who provide written informed consent to participate in this clinical study.

[Rationale]

1) These criteria were established to unify the pathophysiological characteristics of study subjects.

2) The inclusion of patients undergoing laminoplasty for DCM at the intervertebral levels was set to select subjects appropriate for comparing the control treatment (plating) with the experimental treatment (suture anchor fixation), aligning with the study's objective.

3) The age of 20 years or older was set considering the age and capacity at which informed consent can be obtained. The upper age limit of 90 years was set to ensure the safety of the study treatment.

4) This criterion was established to allow the attending surgeon to make a judgment considering all other general factors.

## **6.3　Exclusion Criteria**

Patients meeting any of the following criteria will not be included in this clinical study.

1) Patients for whom cervical laminoplasty is an inappropriate surgical procedure (e.g., patients with significant cervical kyphosis, anterior spinal cord compression, or cervical spinal instability on MRI or CT).

2) Patients with concomitant foraminal stenosis (requiring concomitant posterior foraminal enlargement)

3) Patients with spinal infection

4) Patients with spinal tumors (including metastatic tumors)

5) Patients with traumatic spinal cord injury

6) Patients with a history of cervical spine surgery

7) Patients undergoing maintenance dialysis

8) Patients with cerebral palsy

9) Patients with Parkinson's disease

10) Pregnant women or women who may be pregnant

11) Inappropriate case judged by doctor in charge

[Rationale]

1) Established because they may influence treatment prognosis, including the primary endpoint, the JOA score.

2) Established to standardize the preoperative status (symptoms of myelopathy) of study subjects.

3–9) Established because they may influence prognosis, including the primary endpoint, the JOA score.

10) Established from the perspective of safety and ethical considerations.

11) Established to allow the principal investigator (or co-investigator) to make a judgment considering all other general factors.

# **７．****Matters Concerning Treatment for Study Participants**

## **7.1　Protocol Treatment Implementation Procedure**

**7.1.1　Informed Consent Acquisition**

• The attending surgeons shall confirm the subject's diagnosis and other eligibility criteria, provide a written explanation of this study, obtain the subject's understanding of the study, and acquire written informed consent from the subject.

• For tests conducted to determine eligibility, data obtained within 360 days prior to registration may be used, even if collected before consent acquisition.

**7.1.2　Registration and Randomization**

- After obtaining written consent, the attending surgeon shall reconfirm the subject's eligibility and register the subject in the study EDC system. Registered subjects shall be randomly assigned by the EDC system to either the plate group or the suture anchor group.

※Registration and randomization must be completed by the day before surgery.

**7.1.3　Protocol Treatment**

- Protocol treatment (cervical laminoplasty) must be performed within 90 days of registration.
- If protocol treatment cannot be performed within 90 days for any reason, enter the reason for discontinuation in the case report form as “Protocol Treatment Discontinued”.
- If eligibility criteria are no longer met due to worsening of the primary disease or comorbidities between registration and the protocol treatment date (surgery date), the decision to continue study participation and perform surgery as protocol treatment is left to the discretion of the attending surgeon. The case report form must document that eligibility criteria were no longer met during the period from registration to the treatment date, along with details.
- Laminoplasty for DCM is performed using either the mini-plate or the suture-anchor.
- Surgery is performed under general anesthesia.
- A midline longitudinal skin incision is made from C2 to C7 in the prone position. Dissection is performed up to the medial facet joints.
- Perform a double-opening laminoplasty, expanding 2-4 laminae on the C3-6 vertebrae.
- Resect the tips of the spinous processes to expand the laminae. Use a high-speed drill to make a median longitudinal split in the midline of the laminae. Use the high-speed drill to create a groove at the medial aspect of the intervertebral joints, achieving a suitable hardness for lamina expansion. After creating a median longitudinal split and groove on all planned laminar expansions, gradually expand the lamina using a mucosal elevator. Medially transect the elevated ligamentum flavum, dissect adhesions to the dura mater, and confirm decompression by observing dura mater bulging. Control bleeding from the epidural venous plexus as needed.
- The attending surgeon shall perform the enlarged lamina fixation using either the plate method or the suture anchor method, according to the assignment results at registration.
- Dome-shaped laminectomy may be added to the cephalad and caudal laminae during lamina enlargement.
- Place a drain on the posterior aspect of the lamina prior to closure. Specifications and duration of drain placement are not stipulated.
- Regardless of any concomitant procedures, operative time, blood loss, and intraoperative/postoperative complications shall be included in the surgery. Details shall be recorded in the medical record (or surgical report) and case report.
- For methods other than the expanded lamina fixation technique, the surgical team shall perform the procedure using the settings and methods they deem optimal (see 7.1.4).
- Participation in the study requires that a Japanese Society of Spine Surgery and Related Research (JSSR) certified spine and spinal cord surgery instructor be employed at each participating institution.
- The attending surgeon shall perform surgery using the assigned enlarged lamina fixation method (plate method/suture anchor method) whenever possible. If a different enlarged lamina fixation method is used than the assigned group, the reason must be entered in the case report form. (See 7.1.4)

The procedures for the plate method and suture anchor method are outlined below.

**<Control Group> Mini-Plate**

After laminoplasty, bone holes are created on both sides of the expanded lamina to place LAMINAclip2 (Olympus Terumo Biomaterial Corporation) screws, and the screws are placed.

Using dedicated instruments, place the plate: LAMINAclip2 (Olympus-Terumo Biomaterial Corporation) over the screw head and secure it.

The size of the screws and plates is determined according to the shape of the lamina in each case.

The number of plates used is specified as two or more.


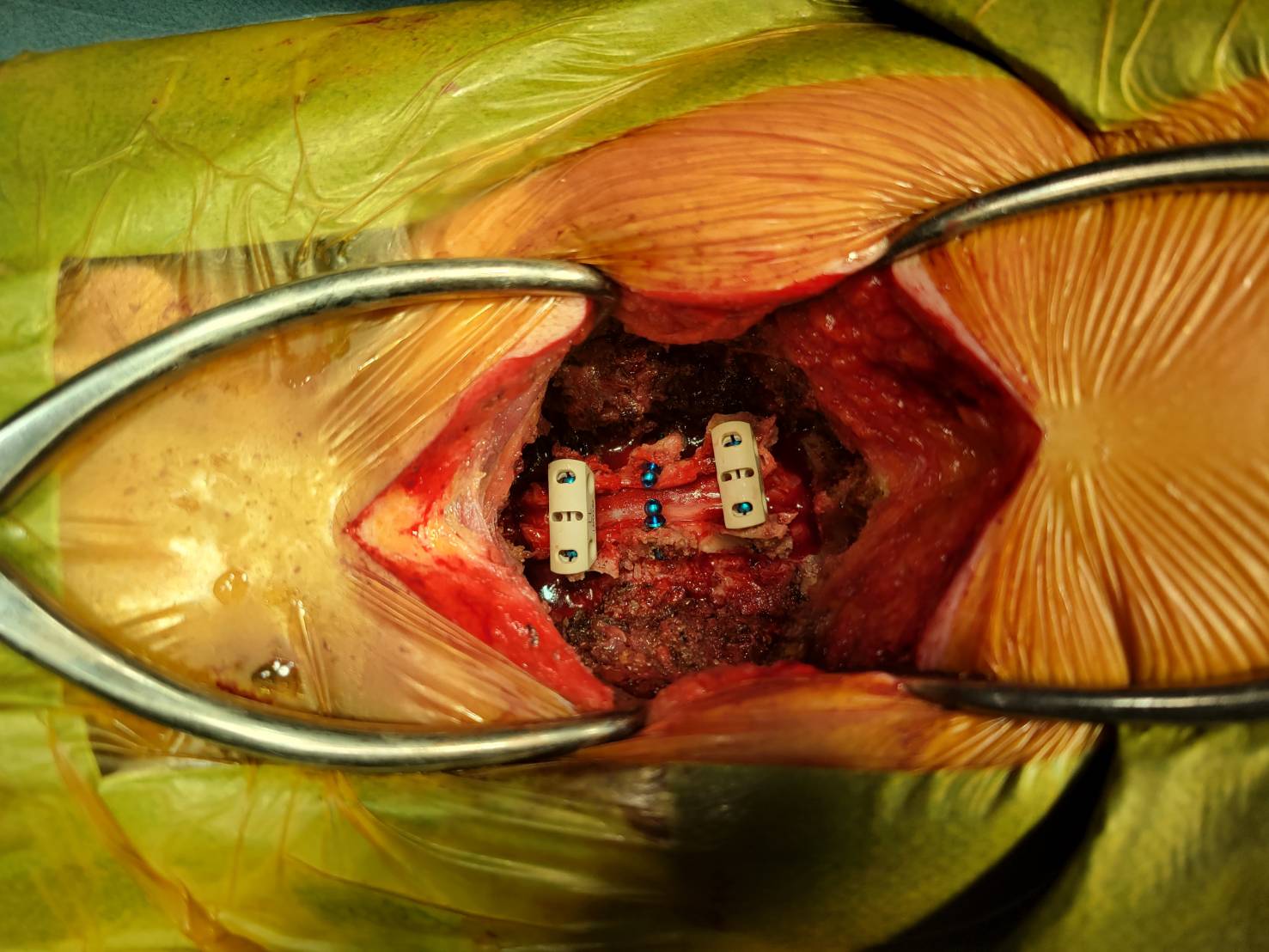

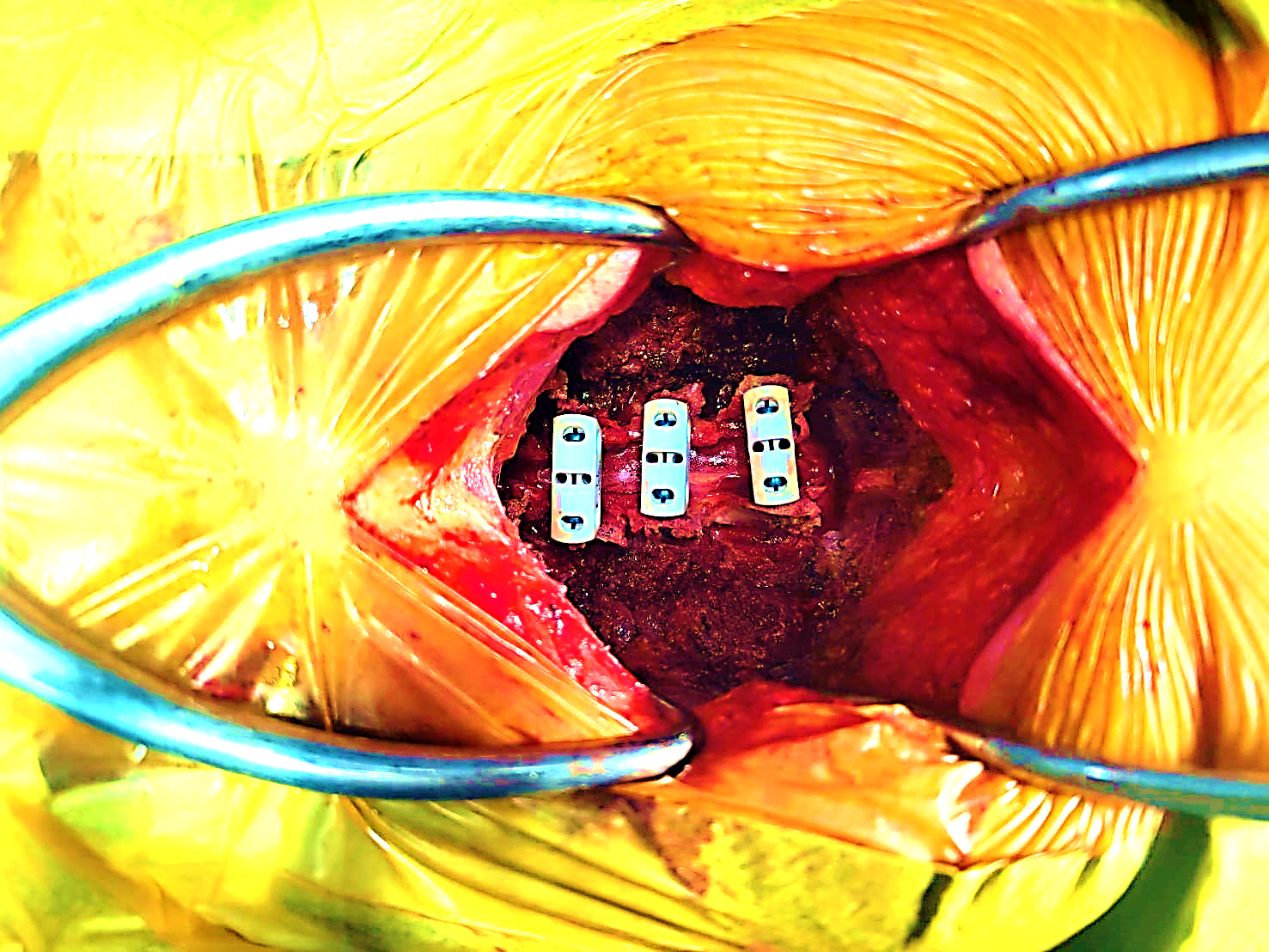

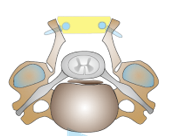


**<Experimental Treatment Group> Suture-Anchor**

After expanding the lamina, place LAMIFIX (Olympus Terumo Biomaterial Corporation) anchors on the lateral masses on both sides of the expanded lamina. Secure the expanded lamina by suturing the suture thread connected to the anchor to the transected yellow ligament between the expanded lamina.


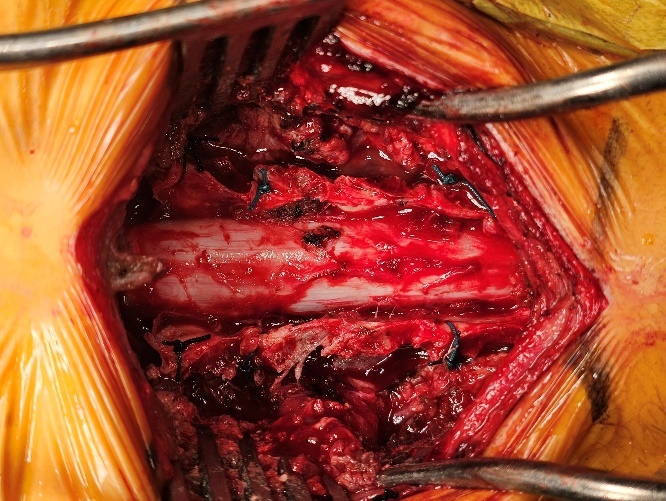

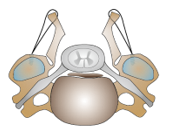


The number of suture anchors used is specified as two or more.

**7.1.4　Regarding Treatment Changes**

<When the Assigned Enlarged Laminar Retention Method Could Not Be Performed>

If the enlarged laminar retention method assigned at registration (mini-plate or suture-anchor) could not be performed for any reason, the attending surgeon shall perform dural decompression using the method they deem best. Prioritize the safety of the study subject and improvement of myelopathy, making appropriate judgments.

The reason the assigned laminoplasty with laminar fixation could not be performed and the laminoplasty with laminar fixation method actually performed must be entered in the case report form.

<If transitioning to posterior or anterior fusion>

If the attending surgeon determines significant cervical instability exists during surgery, or if it is deemed necessary to address intraoperative complications (e.g., laminar fracture, vertebral fracture), transition from laminoplasty to cervical posterior or anterior fusion. Prioritize the safety of the study subject, improvement of myelopathy, and spinal stability, making appropriate judgments.

- If transition to cervical fusion surgery occurs because laminoplasty was impossible, or if transition to cervical fusion surgery occurs before performing laminoplasty, the reasons must be documented in the case report form.
- For the patient in question, the study is discontinued (see 7.6), and surgery for DCM is performed using the settings and methods the surgical team deems optimal.

**7.1.5　Perioperative Management**

Perioperative management includes the type and duration of prophylactic antibiotics, preoperative and postoperative fluid management, timing of postoperative oral intake initiation and dietary content, wound and drain management, and postoperative cervical external fixation. The method of perioperative analgesic use should also follow the standard practice at each participating institution.

The presence or type of postoperative cervical external fixation is not specified, but details of any such fixation must be recorded in the case report form.

The timing of postoperative ambulation and the content of rehabilitation are not specified, but rehabilitation must be performed for all patients.

**7.1.6　Surveillance**

Postoperative investigations shall be conducted according to “7.2 Observation/Examination Items and Schedule”.

If surgical complications or other adverse events are identified, data for the items specified in 7.2.2 shall be collected and entered into the EDC system.

**7.1.7　Follow-up Treatment**

If the assigned expanded laminoplasty with anastomosis cannot be performed, subsequent treatment is not specified. However, the details of any treatment performed must be recorded in the case report form.

**7.2****Observation/Examination Items and Schedule**

**7.2.1　Observation/Examination Schedule**

The survey, observation, and evaluation items, along with the visit schedule for this study, are as follows.

|  | Pre-regist.  ~ Regist. | Surg. Date | PO 1 w | Discharge date | PO 30 d | PO 1y | PO 2 y |  |
| --- | --- | --- | --- | --- | --- | --- | --- | --- |
| Allowed Period |  |  |  |  | Within  ±14d | Within  ±56 d | Within ±56d | |
| Registration/Assignment | ● |  |  |  |  |  |  |  |
| Patient Background Information | ●※1 |  |  |  |  |  |  |  |
| Surgery (Protocol Treatment) |  | ● |  |  |  |  |  | |
| Surgery Information |  | ● |  |  |  |  |  |  |
| Intraoperative Complications/Adverse Events |  | ● |  |  |  |  |  |  |
| Early Postoperative Complications/Adverse Events |  |  |  |  |  |  |  |  |
| Medical History/Physical Examination | ●※1 |  | ● | ● | ● | ● | ● |  |
| JOA score | ●※1 |  |  |  |  | ● | ● |  |
| Cervical Plain X-ray  (Cervical Alignment) | ●※1 |  | ● |  |  | ● | ● |  |
| Cervical Plain CT  (Postoperative: Enlarged Laminar Retention Rate, Hinge Fracture/Bone Union) | ●※1 |  | ● |  |  | ● | ● |  |
| Cervical Plain MRI  (Paraspinal Muscle Cross-sectional Area) | ●※1 |  |  |  |  | ● | ● |  |
| Cervical Plain MRI  (Dural sac Area) | ●※1 |  |  |  |  | ● | ● |  |
| Cervical Plain MRI  (Grading of the mass posterior to the dural sac) |  |  |  |  |  | ● | ● |  |
| Late Postoperative Complications/Adverse Events |  |  |  |  |  |  |  |  |
| Health-Related QoL（EQ-5D） | ●※1 |  |  |  |  | ● | ● |  |
| Pain/Numbness Severity (VAS) | ●※1 |  |  |  |  | ● | ● |  |
| Cervical Disability Index (NDI) | ●※1 |  |  |  |  | ● | ● |  |
| Outcome Follow-up |  |  |  |  |  | ● | ● |  |
| Direct Medical Costs |  |  |  |  |  |  |  |  |

● ：Required

※1 : All surveys, observations, and tests related to this clinical study will be conducted after obtaining written consent from the research subjects. However, results from surveys, observations, tests, etc., performed as part of routine care prior to obtaining consent may be used as research data if they were conducted within 360 days prior to registration.

**7.2.2　Observation and Examination Items**

The observation and examination items for this study are as follows. The timing of implementation follows “7.2.1 Schedule”.

| Implementation Item | | Observation/Examination Item | |
| --- | --- | --- | --- |
| Consent Acquisition/Registration/Assignment | | Date of Written Consent Acquisition | |
| Patient Background Information | General Findings | Gender, Date of Birth, Physical Findings (Height, Weight, BMI), Smoking History,  Comorbidities, ASA (American Society of Anesthesiologists) Classification, Oral Medications (Antithrombotic Agents, Steroids, etc.), Occupation, Duration of Illness, History of Cervical Spine Surgery | |
|  | Clinical/Imaging Findings of DCM | Preoperative Diagnosis | CSM/OPLL |
|  |  | JOA score | 17 points |
|  |  | Health-Related QOL（EQ-5D） | 0-1 |
|  |  | Severity of Neck Pain, Upper Limb Pain, Upper Limb Numbness (VAS) | 0-100mm |
|  |  | NDI | Maximum score 50 |
|  |  | Plain X-ray | Cervical spine range of motion, sagittal balance (C2-7 angle, C-SVA, C7 slope),  For OPLL: Ossification lesion morphology (segmental/continuous/mixed), highest level and maximum ossification occupancy rate |
|  |  | MRI | Level of maximum stenosis, Level of T2 sagittal intramedullary signal change, C4/5 paraspinal muscle cross-sectional area (mm^2^) |
| Surgical Information | | Date of surgery, Participation of spinal surery specialist (None / Participated as surgeon / Participated as assistant), Surgical procedure (level of laminoplasty, Presence/absence of dome-shaped laminectomy), Operative time, Blood loss, method (mini-plate/suture-anchor), implant type (mini-plate/suture-anchor), implant size (length, dimensions), blood transfusion volume (intraoperative and up to 3 days postoperatively), type of hemostatic agent used, presence of drain, transition to cervical fusion (none/transition to posterior cervical fusion/transition to anterior cervical fusion) | |
| Intraoperative Complications/Adverse Events | | If any occur, record the complication name, outcome, response to the complication, relationship to cervical spine surgery, treatment details/date of treatment, and whether hospitalization was extended. | |
| Postoperative Course | | Type/presence/duration of postoperative external fixation  Admission date/discharge date → Postoperative hospital stay days, Outcome at discharge, Whether reoperation was performed during hospitalization | |
| Early Postoperative Complications/Adverse Events (within 30 days postoperatively) | | Confirm the presence of adverse events through medical history and physical examination.  If an adverse event occurs, document the event name, onset time, outcome, severity (including whether reoperation was required), relationship to cervical spine surgery, treatment details/date of treatment, and whether hospitalization was extended. | |
| Medical History/Physical Examination | | Conduct medical history and physical examination during regular postoperative outpatient visits. | |
| Plain X-ray | | Cervical spine (AP/Lateral/Anterior flexion lateral/Posterior flexion lateral): Confirm C2-7 angle/C-SVA/C7 slope  Full spine standing (AP/Lateral): Confirm SVA/Lumbar lordosis angle/Thoracic kyphosis angle/Pelvic incidence, check for existing vertebral fractures | |
| Cervical Plain CT | | Horizontal, sagittal, and coronal slices at each intervertebral level: Confirm presence of OPLL/Lamina retention rate, hinge fractures/gutter bone union. | |
| Cervical Plain MRI | | T2 and T1 sagittal/horizontal images  Preoperative: Maximum stenosis level, presence of intramedullary signal changes, C4/5 paraspinal muscle area, dural sac area  Postoperative: C4/5 paraspinal muscle area, dural sac area, Grading of the mass posterior to the dural sac | |
| Late Postoperative Complications/Adverse Events (From 31 days post-op to within 2 years post-op) | | If an adverse event occurs, document: event name, onset timing, severity, outcome, seriousness (whether reoperation required), relationship to cervical spine surgery, treatment details/date, and hospitalization status. | |
| Outcome Investigation | | Survival (Last confirmed survival date, presence/absence of death, date of death, cause of death)  Reoperation (Reoperation presence/absence, reoperation date, reason for reoperation, reoperation method)  Study Discontinuation (Discontinuation presence/absence, discontinuation date, reason for discontinuation) | |
| Direct Medical Costs | | Hospitalization Costs (Medical fees excluding surgery, surgery-related costs [surgery, surgical drugs, anesthesia, anesthetic drugs])  Outpatient Costs (Consultations, tests, drugs, other)  Hospitalization Re-treatment Costs for Adverse Events (Hospitalization medical fees excluding surgery, surgery-related costs [surgery, surgical drugs, anesthesia, anesthetic drugs])  Research collaborators collect the above costs from medical claims. | |

**7.3　 Concomitant Medications (Therapies) Regulations**

**7.3.1** **Concomitant Restricted Medications (Therapies)**

None in particular

**7.3.2** **Concomitant Prohibited Medications (Therapies)**

None in particular

**7.4** **Instructions for Research Participants**

The principal (sub-investigator) investigator shall provide the following instructions to research participants before the study begins.

1) Advise participants that when visiting other departments or hospitals, they must inform the attending surgeon or pharmacist of their participation in the study and, whenever possible, consult with their attending surgeon beforehand.

If prior consultation is not possible, instruct them to report to their attending surgeon afterward.

2) Advise participants that if they experience any physical discomfort, they should promptly report it to their attending surgeon and consult about the need for medical care.

3) Instruct participants not to disclose information about this clinical research on social media or similar platforms.

**7.5　 Post-Study Measures**

Efforts will be made to ensure research subjects can receive the best possible diagnosis and treatment based on the study results even after their participation ends.

**7.6　 Individual Research Discontinuation Criteria**

The study will be discontinued if any of the following criteria are met.

1) If the protocol treatment (laminoplasty) cannot be performed within 90 days of registration.

2) If the research subject voluntarily requests to withdraw consent.

3) If, after registration, imaging or clinical diagnosis determines that laminoplasty is not indicated.

4) If intraoperative findings indicate laminoplasty is impossible or if cervical fusion is performed instead of laminoplasty.

5) If intraoperative complications (lamina fracture, vertebral body fracture) necessitate fixation during or after surgery

6) If a major deviation from the protocol is identified, such as violation of the Clinical Research Act and its enforcement regulations, selection criteria, or exclusion criteria

7) If compliance with the protocol becomes impossible

8) If the subject becomes pregnant

9) If the entire study is discontinued

10) If the research subject dies

11) Other cases where the principal investigator or attending surgeon determines continuation of the study is difficult

*The protocol treatment discontinuation date is defined as: the date of determination for cases 1-3), 5)-9), and 11); the surgery date for case 4); and the date of death for case 10).

[Rationale]

These criteria were established to ensure ethical research conduct and to safeguard the safety of research subjects.

[Procedure upon Discontinuation]

When discontinuing the study due to meeting discontinuation criteria, the principal investigator and co-investigators shall take appropriate measures for the subject and clearly document the date/timing of discontinuation, reason, and course of events in the medical records.

Furthermore, if consent is withdrawn after the start of the study treatment, efforts shall be made to clarify whether the cause is due to lack of efficacy of the drug, adverse events (or disease, etc.), or incidental events

(such as relocation). If clinically significant abnormalities (including laboratory values) are identified, appropriate tests shall be performed, and follow-up shall continue until values return to medically acceptable ranges or until the attending surgeon determines follow-up is unnecessary. For adverse events (or diseases, etc.) persisting after study discontinuation, follow-up shall continue until the event resolves or the attending surgeon determines follow-up is unnecessary.

Data obtained from research subjects who withdraw their consent to participate in the study shall be handled according to the subject's wishes: either anonymized and used for research analysis up to the point of submitting the withdrawal of consent form, or all information shall be destroyed.

**８．Evaluation Matters**

**8.1** **Evaluation Indicators (Endpoints)**

**8.1.1　 Primary endpoint**

Recovery rate in cervical JOA score before and after surgery

**8.1.2　Secondary endpoint**

(1) Operative time

(2) Estimated blood loss

(3) Proportion achieving the MCID for recovery in cervical JOA score at postoperative 1 and 2 years

(4) Health-related QOL (EQ-5D) at postoperative 1 and 2 years

(5) Severity of neck pain, upper limb pain, and upper limb numbness (VAS) at postoperative 1 and 2 years

(6) NDI at postoperative 1 and 2 years compared to preoperative

(7) Retention rate of expanded lamina at postoperative 1 and 2 years ^3)^

(8) Proportion of hinge fractures at postoperative 1 and 2 years

(9) Percentage of gutter bone union at postoperative 1 and 2 years^4)^

(10) Cervical spine alignment at 1 and 2 years post-surgery (C-SVA, C2-7 angle, C7 slope)

(11) Paraspinal muscle cross-sectional area (C4/5 level) at postoperative 1 and 2 years

(12) Dural sac area (C3/4, C4/5, C5/6, C6/7 levels) at postoperative 1 and 2 years

(13) Grading of the mass posterior to the dural sac at postoperative 1 and 2 years

(14) Incremental Cost-Effectiveness Ratio (ICER)

(15) Surgical Complication Rates

・Intraoperative complication rate (from start of surgery to completion/closure)

・Early postoperative complication rate (from completion/closure to within 30 days postoperatively)

・Late postoperative complication rate (from postoperative day 31 to within 2 years)

・Mortality rate (entire period: includes non-surgery-related deaths)

・Reoperation rate within 2 years

*For evaluating intraoperative/postoperative complications, use the evaluation criteria from the Japanese Society for Spine Surgery and Related Research database (JSSR-DB) (<https://ssl.jssr.gr.jp/db/>).

**8.2** **Evaluation and Recording of Assessment Indicators**

**＜Primary Endpoints＞**

Recovery rate in cervical JOA score before and after surgery

［Definition］

Use the cervical JOA score assessed by the surgeon before surgery and at postoperative 1 or 2 years. The recovery rate calculation formula is as previously reported^4)^, using the following formula:

Cervical JOA Score Recovery Rate = (Postoperative Score - Preoperative Score) / (17 - Preoperative Score) × 100 (%)

The primary analysis time point is postoperative 1 year. Results at postoperative 2 years are also presented as a final analysis.

The same applies to secondary endpoints 3) to 13).


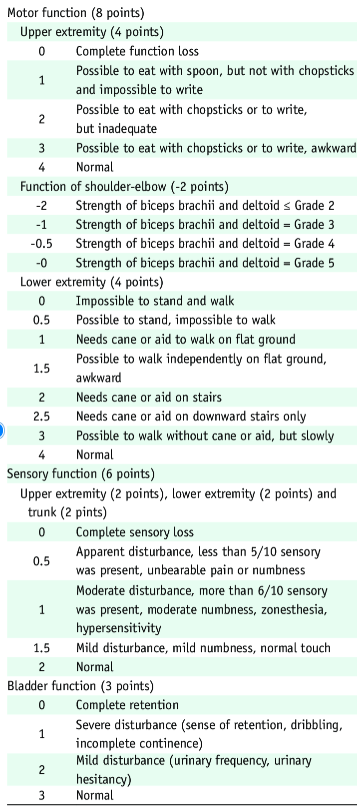


Cervical JOA Score (Hirabayashi R et al: Japanese Orthopaedic Association Criteria for Evaluating Treatment Outcomes of Cervical Spinal Cord Disorders. J Orthop Surg Jpn, 68:490-503.1994)

**＜Secondary Endpoints＞**

(1) Operative Time

(2) Estimated Blood loss

[Definition]

Operating time is defined as the time recorded in the anesthesia record.

Blood loss is defined as the amount recorded in the anesthesia log. If “minor bleeding” is recorded, it is considered 10 ml of blood loss.

(3) Proportion achieving the MCID in cervical JOA score recovery rate at postoperative 1 and 2 years

[Definition]

Refer to the description under <Primary Evaluation Items> for the definition of JOA score recovery rate.

Following previous reports19), the MCID for JOA score recovery rate is set at 52.8%. The MCID achievement rate at 1 year and 2 years postoperatively is evaluated for each group.

(4)　 Health-related QOL (EQ-5D) at postoperative 1 and 2 years

[Definition]

Obtained via patient self-completion of the Japanese-language questionnaire (attached separately). If the patient cannot complete the questionnaire due to writing difficulties caused by cervical myelopathy, family members or healthcare professionals will assist with completion.

Health-related QOL will be assessed using the EQ-5D before surgery, postoperative 1 year, and 2 years. Note: The EQ-5D is also used as the utility value when calculating the incremental cost-effectiveness ratio (11).

(5) Severity of neck pain, upper limb pain, and upper limb numbness (VAS) at postoperative 1 and 2 years

[Definition]

Obtained through the patient's own completion of a questionnaire form created in Japanese (attached separately). If the patient cannot complete the questionnaire due to writing difficulties caused by cervical myelopathy, a family member or healthcare professional will assist with completion.

The severity of pain and numbness will be assessed using a 0–100 mm VAS before surgery and at postoperative 1 and 2 years.

(6) Neck disability index (NDI) at postoperative 1 and 2 years compared to preoperative

[Definition]

Data is collected via the Japanese version questionnaire (attached separately) completed by the patient themselves. If the patient cannot complete the questionnaire due to writing difficulties caused by cervical myelopathy, family members or healthcare professionals will assist with completion.

Health-related QOL due to cervical spine disease is assessed before surgery, and postoperative 1 year and 2 years using the NDI total score.


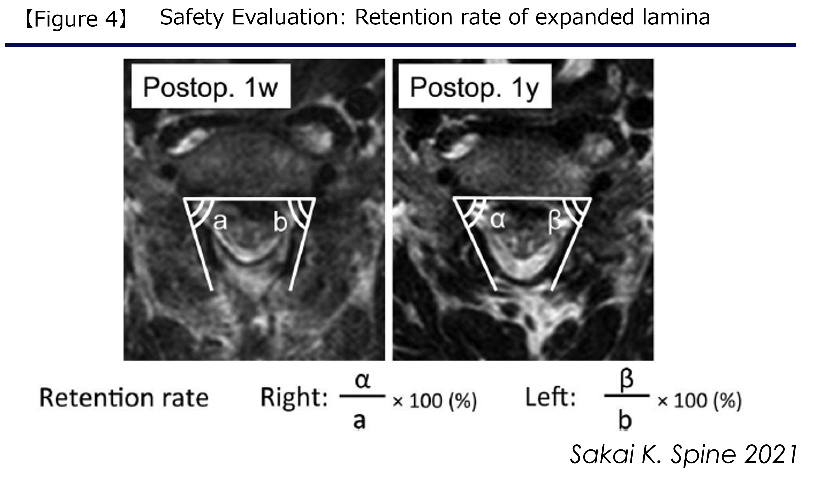
(7) Retention rate of enlarged lamina at postoperative 1 and 2 years (Retention rate)

[Definition]

The retention rate is calculated for each enlarged lamina identified on CT scans at postoperative 1 and 2 years, following previous reports^7)^ (Figure 4). The mean retention rate is also calculated for each patient.

(8) Proportion of hinge fractures at postoperative 1 and 2 years

(9) Percentage of bone union of gutter at postoperative 1 and 2 years

[Definition]

Using CT images at 1 and 2 years postoperatively, evaluate the presence or absence of hinge fractures (fractures of the groove created in the medial facet joint) and bone union in each enlarged lamina, following previous reports^8)^. Evaluate both on horizontal slices. A fracture is defined as a complete disruption of the cortical bone continuity of the target lamina. and bone union is defined as the formation of even partial bony continuity within the excavated groove region of the target lamina. The level and lateral side of each lamina are recorded.


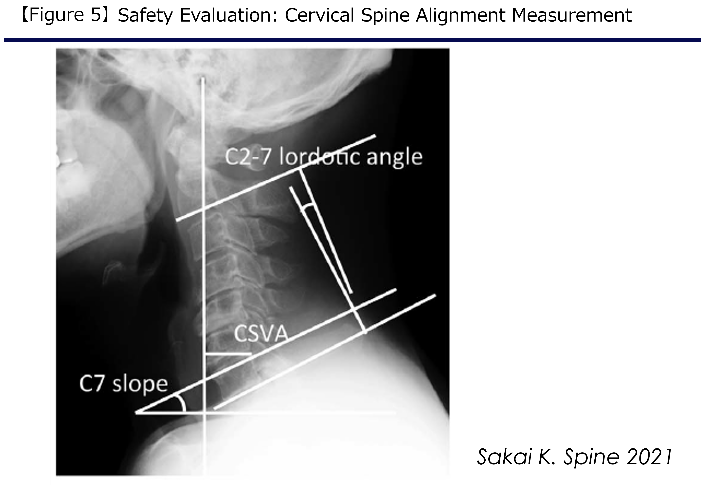
(10) Sagittal alignment of cervical spine at postoperative 1 and 2 years (C-SVA, C2-7 angle, T1 slope)

[Definition]

Cervical spine alignment (C-SVA, C2-7 angle, C7 slope) is measured from plain radiographs at postoperative 1 and 2 years, according to previous reports^7)^ (Figure 5). Note that the C2-7 angle is denoted with a positive (+) sign for lordosis.

(11) Paraspinal muscle cross-sectional area (C4/5 level) at postoperative 1 and 2 years


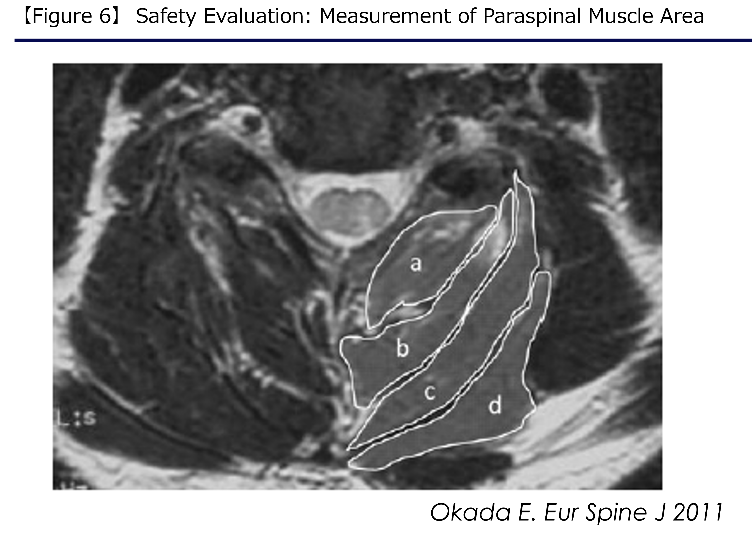
[Definition]

Measure the cross-sectional area of paraspinal muscles (mm^2^) on the C4/5 slice of T2-weighted images according to a previous report^20)^, and calculate the total values for each side (Figure 6).

(a: multifidus, b: cervical semispinalis, c: cervical splenius capitis, d: splenius capitis)

(12) Dural sac cross-sectional area (C3/4, C4/5, C5/6, C6/7 levels) at postoperative 1 and 2 years

[Definition]

Measure the dural canal cross-sectional area (mm^2^) on the T2-weighted horizontal slice at each of the C3/4, C4/5, C5/6, and C6/7 levels, following the method described in a previous report^21)^ (Figure 7).

(13) Grading of the mass posterior to the dural sac at postoperative 1 and 2 years

［Definition］

The classification of posterior dural canal compression on the central slice of the T2 sagittal image is evaluated according to a previous report^22)^ (Figure 7).


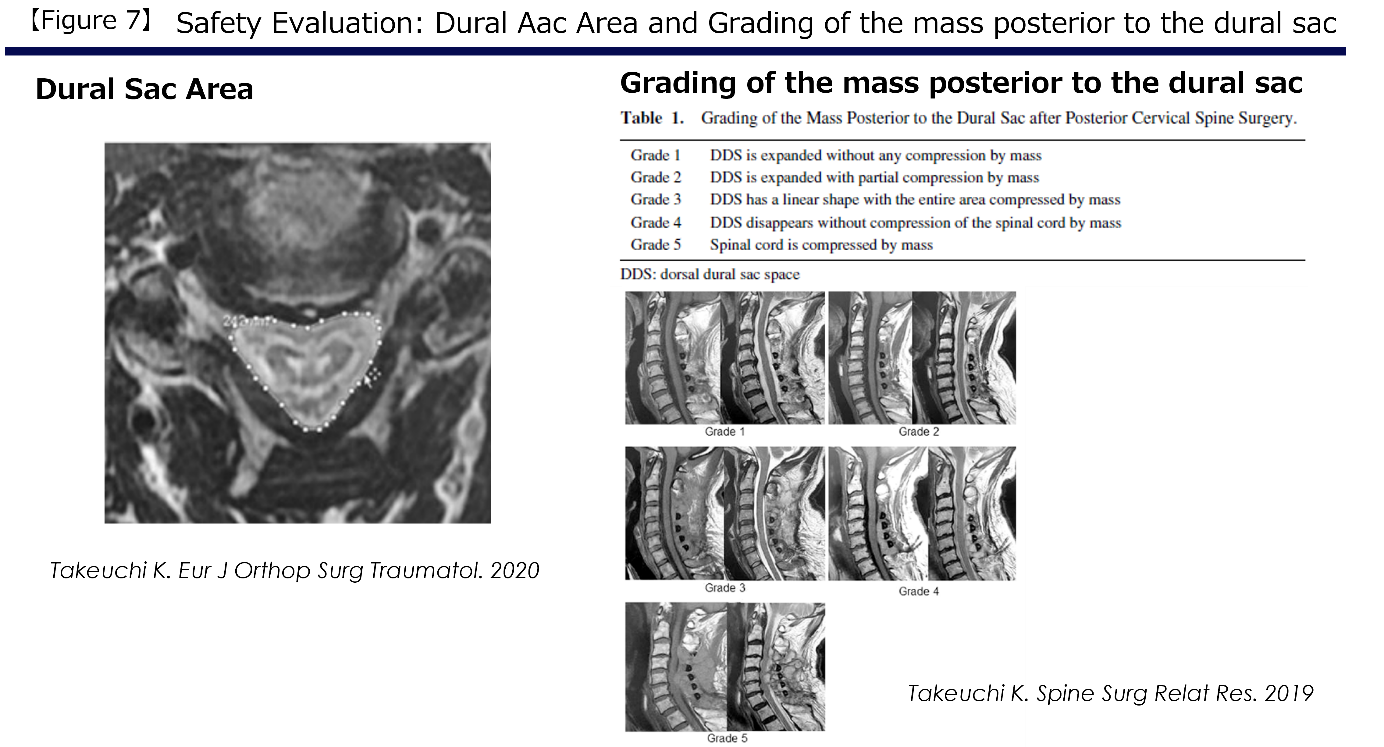


(14) Incremental Cost-Effectiveness Ratio (ICER)

[Definition]

Collect direct medical costs at the facility where surgery was performed for each patient at discharge, and at 1 and 2 years post-surgery. Direct medical costs include inpatient treatment costs and outpatient treatment costs for the following services, summing ①, ②, and ③.

① Inpatient treatment costs for DCM surgery (surgery-related costs [surgery, surgical drugs, anesthesia, anesthetic drugs]), excluding surgical fees

② Outpatient costs for DCM after the surgery date (consultations, tests, drugs, other)

③ Rehospitalization costs for adverse events (inpatient treatment fees excluding surgery, surgery-related costs [surgery, surgical drugs, anesthesia, anesthetic drugs])

The cost-effectiveness evaluation metric uses the Incremental Cost-Effectiveness Ratio (ICER). The ICER is calculated by subtracting the control group's costs from the trial treatment group's costs. (ICER). ICER is calculated by dividing the incremental cost—the difference between the trial treatment group's costs and the control group's costs—by the incremental effect—the difference between the trial treatment group's effect and the control group's effect.

The outcome measure used is QALY (quality-adjusted life year). QALY is calculated using a Markov model that accounts for patients requiring reoperation or experiencing death, based on QOL measured by EQ-5D. Utility values and cost values are discounted annually at a rate of 2%, as recommended by the Central Social Insurance Medical Council (https://c2h.niph.go.jp/tools/guideline/guideline_ja.pdf).

(15) Surgical complication incidence

Surgical complications are categorized into intraoperative, early postoperative (within 30 days after surgery completion [wound closure]), and late postoperative (from day 31 after surgery up to 2 years postoperatively) complication rates.

Mortality (all deaths during the study period) and reoperation rate within 2 years postoperatively are recorded independently for complications.

**・Intraoperative complication rate**

[Definition]

Report complications occurring from the start of surgery to its completion (wound closure), using the study population as the denominator. As complications of particular focus, extract the complications listed below (Table A) according to the registration items of the Japanese Spine Society Registry (JSSR-DB: https://ssl.jssr.gr.jp/db/) and report their frequency and rates.

When a complication occurs, the following details must be documented in the medical record and case report: complication name, outcome, management approach, severity, relationship to cervical spine surgery, treatment details/date of treatment, and whether hospitalization was prolonged.

Table A: Intraoperative Complications Requiring Special Attention

| **Complication** | **Management** |
| --- | --- |
| Dural tear | Primary repair, fibrin glue, artificial dura, fat grafting, spinal drainage, other |
| Massive bleeding (2000ml or more) | Allogeneic blood transfusion, autologous blood transfusion, intraoperative blood recovery, observation, other |
| Nerve Injury (Spinal Cord)/Nerve Injury (Nerve Root) | Steroid administration, observation, laminectomy, etc., other |
| Major Vessel Injury/Vertebral Artery Injury/Other Arterial Injury | Pressure hemostasis only, suturing by relevant department, specialist (vascular surgery, etc.) intervention, catheter, other |
| Vertebral body injury/Transverse process fracture | Implant addition/replacement, Other |
| Misidentification of level | Surgery performed on incorrect upper vertebra(e), Surgery performed on incorrect lower vertebra(e), Other |
| Change to conventional method | Conventional method (Kirita-Miyazaki method): (Level changed), Laminectomy: (Level changed), Other |

**・Early postoperative complication incidence rate (within 30 days after surgery completion (wound closure))**

[Definition]

Using the analysis population as the denominator, describe complications occurring within 30 days after surgery completion (wound closure). For complications of particular focus, extract those listed in the Japanese Spine Society Registry (JSSR-DB: https://ssl.jssr.gr.jp/db/).

If a complication occurs, document the complication name, outcome, management of the complication, severity, relationship to cervical spine surgery, treatment details/date of treatment, and whether hospitalization was prolonged.

Table B: Early Postoperative Complications Requiring Special Attention (Surgical-Related)

| **Complication** | **Management** |
| --- | --- |
| Vascular Injury (Major Vessel Injury/Vertebral Artery Injury/Other Vascular Injury) | Observation, Management by Relevant Department, Management by Specialist (Vascular Surgery, etc.), Catheterization, Other |
| Epidural hematoma | Laminectomy, hematoma evacuation |
| Neurological Symptoms/Muscle Strength Related (Postoperative Upper Limb Paralysis/Postoperative Lower Limb Paralysis/Postoperative Sensory Nerve Paralysis/Bladder/Rectal Dysfunction/Other Complications) | Observation, reoperation, steroid administration, other drug therapy, other |
| Spinal Support Tissue Injury (Vertebral Body Injury/Transverse Process Fracture) | Implant Addition/Replacement/Other |
| Dural Injury (Cerebrospinal Fluid Leak/Meningitis/Intracranial Lesion [subdural hematoma, etc.]) | Conservative management (observation/spinal drainage), reoperation (additional procedures, dural repair, fibrin glue, artificial dura, fat grafting, spinal drainage), antibiotic administration |
| Fixed Metal-Related (Implant fracture, Implant dislocation, Screw malposition) | Observation, implant replacement, implant addition, other  Surgical Site Infection (Superficial/Deep)  Cleaning, Irrigation, Implant Removal, Other |
| Surgical Site Infection (Superficial/Deep) | Wash-out, Irrigation, Implant Removal, Other |
| Misidentification of Level | Surgery on Upper Vertebrae (Intervertebral Space), Surgery on Lower Vertebrae (Intervertebral Space), Other |
| Other (Postoperative Impairment Due to Positioning), Ocular Impairment [Corneal Injury, etc.], Blindness, Dysphagia, Other | Observation, Additional Surgery |

Table C: Particularly Noteworthy Early Postoperative Complications (Systemic Complications))

| **Complication** | **Management** |
| --- | --- |
| Acute Heart Failure | Observation, Conservative Treatment, Endoscopic Treatment/IVR, etc., Surgical Treatment, Other |
| Myocardial Infarction | Observation, Conservative Treatment, Endoscopic Treatment/IVR, etc., Surgical Treatment, Other |
| Pulmonary infarction | Observation, Conservative Treatment, Endoscopic Treatment/IVR, etc., Surgical Treatment, Other |
| Pulmonary embolism | Observation, Conservative Treatment, Endoscopic Treatment/IVR, etc., Surgical Treatment, Other |
| Peripheral venous thrombosis (symptomatic) | Observation, Conservative Treatment, Endoscopic Treatment/IVR, etc., Surgical Treatment, Other |
| Respiratory Failure | Observation, Conservative Treatment, Endoscopic Treatment/IVR, etc., Surgical Treatment, Other |
| Aspiration Pneumonia | Observation, Conservative Treatment, Endoscopic Treatment/IVR, etc., Surgical Treatment, Other |
| Pneumonia | Observation, Conservative Treatment, Endoscopic Treatment/IVR, etc., Surgical Treatment, Other |
| Atelectasis | Observation, Conservative Treatment, Endoscopic Treatment/IVR, etc., Surgical Treatment, Other |
| Pulmonary Edema | Observation, Conservative Treatment, Endoscopic Treatment/IVR, etc., Surgical Treatment, Other |
| Cerebral Infarction | Observation, Conservative Treatment, Endoscopic Treatment/IVR, etc., Surgical Treatment, Other |
| Postoperative delirium | Observation, Conservative Treatment, Endoscopic Treatment/IVR, etc., Surgical Treatment, Other |
| Dementia | Observation, Conservative Treatment, Endoscopic Treatment/IVR, etc., Surgical Treatment, Other |
| Intestinal obstruction | Observation, Conservative Treatment, Endoscopic Treatment/IVR, etc., Surgical Treatment, Other |
| Pseudomembranous colitis | Observation, Conservative Treatment, Endoscopic Treatment/IVR, etc., Surgical Treatment, Other |
| Gastrointestinal Perforation/Gastric Perforation | Observation, Conservative Treatment, Endoscopic Treatment/IVR, etc., Surgical Treatment, Other |
| Pancreatitis | Observation, Conservative Treatment, Endoscopic Treatment/IVR, etc., Surgical Treatment, Other |
| Hepatitis | Observation, Conservative Treatment, Endoscopic Treatment/IVR, etc., Surgical Treatment, Other |
| Cholangitis | Observation, Conservative Treatment, Endoscopic Treatment/IVR, etc., Surgical Treatment, Other |
| Acute Renal Failure | Observation, Conservative Treatment, Endoscopic Treatment/IVR, etc., Surgical Treatment, Other |
| Urinary Tract Infection | Observation, Conservative Treatment, Endoscopic Treatment/IVR, etc., Surgical Treatment, Other |
| DIC | Observation, Conservative Treatment, Endoscopic Treatment/IVR, etc., Surgical Treatment, Other |
| Sepsis | Observation, Conservative Treatment, Endoscopic Treatment/IVR, etc., Surgical Treatment, Other |
| Fractures Due to Falls During Hospitalization | Observation, Conservative Treatment, Endoscopic Treatment/IVR, etc., Surgical Treatment, Other |
| Other | Observation, Conservative Treatment, Endoscopic Treatment/IVR, etc., Surgical Treatment, Other |

**Late Postoperative Complication Incidence Rate (31 days to 2 years postoperatively)**

[Definition]

Describe complications occurring between 31 days and 2 years postoperatively, using the analysis population as the denominator. The following complications (Table D) are specifically highlighted; their frequency and rates are documented.

When a complication occurs, the following details must be recorded in the medical records and case report: complication name, outcome, management of the complication, relationship to cervical spine surgery, treatment details/date of treatment, and whether hospitalization was prolonged.

**Table D: Particularly Noteworthy Late Postoperative Complications**

| **Complication** | **Complication Details and Management** |
| --- | --- |
| Neurological Symptoms | Location (operated intervertebral space, adjacent intervertebral space, other location), responsible level, management, outcome |
| Local Kyphosis Worsening | Level, management, outcome |
| Slippage Worsening | Level, anterior/posterior, management, outcome |
| Systemic Complications | Name, Management, Outcome |
| Instrument-related | Loosening/Displacement, Management, Outcome |
| Infection | Superficial/Deep, Management, Outcome |
| Other | Complication Name, Management, Outcome |

**8.3** **Methods and Timing for Analyzing Evaluation Indicators**

Refer to “10. Matters Concerning Statistical Analysis”.

**９．Matters Concerning Recording, Reporting, etc. of Adverse Events, Surgical Complications, and Malfunctions**

**9.1** **Methods for Recording, Reporting, and Analyzing Adverse Events, Surgical Complications, and Malfunctions**

**１）Adverse Events**

An adverse event refers to any unfavorable or unintended injury or illness (including abnormal laboratory values) occurring in a research subject, regardless of whether it is causally related to the conducted study.

Symptoms or diseases present prior to registration shall be treated as pre-existing conditions and not considered adverse events. However, surgical complications (described later) are also included as adverse events. In this study, adverse events shall be categorized and tallied separately as intraoperative surgical complications, early postoperative complications (surgery-related/systemic complications), and late postoperative complications, as outlined in Table A to D in Section 8.2(9).

The evaluation period shall extend up to 2 years post-surgery.

When an adverse event occurs, the following details shall be recorded in the medical records and case report form: complication name, outcome, management of the complication, relationship to cervical spine surgery, treatment details/date of treatment, and whether hospitalization was prolonged. Additionally, the occurrence status shall be periodically compiled and recorded in the monitoring report.

**2) Surgical Complications**

Surgical complications in this study are defined as any undesirable or unintended illness or injury (including abnormal laboratory values) that occurs in study subjects in relation to the surgery performed as part of the protocol treatment. Surgical complications are classified as: general anesthesia complications; intraoperative complications (from the start of surgery to its completion [wound closure]); early postoperative complications (from completion of surgery [wound closure] to within 30 days postoperatively); and late postoperative complications (from 31 days postoperatively to within 2 years postoperatively). The evaluation period extends up to 2 years postoperatively.

When a surgical complication occurs, the complication name, outcome, management of the complication, severity, treatment details/date of treatment, and whether hospitalization was prolonged must be documented in the medical record and case report. Additionally, occurrence data must be periodically compiled and recorded in a monitoring report.

Severity is determined based on the Common Terminology Criteria for Adverse Events (CTCAE) version 5.0.

Grade 1: Mild, asymptomatic or mild symptoms, clinical or laboratory findings only, no treatment required

Grade 2: Moderate, requires minimal, localized, non-invasive treatment, limitations in activities of daily living beyond age-appropriate self-care

Grade 3: Severe or medically significant but not immediately life-threatening, requiring hospitalization or prolongation of hospitalization, incapacitation or inability to perform activities, limitation in activities of daily living

Grade 4: Life-threatening, requiring urgent intervention

Grade 5: Death due to the adverse event

1. **Serious Adverse Events and Surgical Complications**

The following are considered serious adverse events or surgical complications:

1) Death

2) Diseases or conditions that may lead to death

3) Diseases or conditions requiring hospitalization or extended hospitalization for treatment

4) Disability

5) Diseases or conditions that may lead to disability

6) Diseases or conditions deemed serious equivalent to 1) to 5)

7) Congenital diseases or abnormalities in subsequent generations

The evaluation period shall be up to two years post-surgery.

Event names, complication names, outcomes, responses to complications, treatment details/treatment dates, and whether hospitalization was extended shall be recorded in medical records and case reports. Occurrence data shall be periodically compiled and recorded in monitoring reports.

**４）Malfunctions**

A malfunction refers broadly to any poor condition of the device related to quality, safety, performance, etc., such as device damage or operational failure, regardless of whether it occurs during design, delivery, storage, or use. In this study, if an event qualifying as a malfunction occurs with the screws and mini-plates used in the control group (plate method) or with the suture anchors used in the test treatment group (suture anchor method), it shall be recorded in the medical records and case report forms. Additionally, the occurrence status will be periodically compiled and recorded in the monitoring report. Furthermore, as necessary, information regarding the malfunction will be shared with the manufacturer of the device (in a form that does not include personal information).

**9.2** **Anticipated Adverse Events**

　　The anticipated adverse events (including surgical complications) in this study are as follows.

1) Intraoperative Complications

• Dural injury

• Massive bleeding (2000 ml or more)

• Nerve injury (spinal cord)/Nerve injury (nerve root)

• Major vessel injury/Vertebral artery injury/Other arterial injury

• Vertebral body injury/Transverse process fracture

• Misidentification of level

• Conversion to conventional method

2) Early postoperative complications (surgery-related)

• Vascular injury (major vessel injury/Vertebral artery injury/Other vascular injury)

• Epidural hematoma

• Neurological/muscle strength related (Postoperative upper limb paralysis/Postoperative lower limb paralysis/Postoperative sensory nerve paralysis/Bladder/rectal dysfunction/Other complications)

• Spinal support tissue injury (Vertebral body injury/Ligamentum flavum fracture)

• Dural injury (Cerebrospinal fluid leak/Meningitis/Intracranial lesion [Subdural hematoma, etc.])

• Implant-related (Implant fracture, Implant dislocation, Screw malposition)

• Surgical Site Infection (Superficial/Deep)

• Misidentification of level

• Other (Postoperative complications due to positioning), Eye complications [Corneal injury, etc.], Blindness, Dysphagia

Early postoperative complications (Systemic complications)

• Acute heart failure

• Myocardial infarction

• Pulmonary infarction

• Pulmonary embolism

• Peripheral venous thrombosis (symptomatic)

• Respiratory failure

• Aspiration pneumonia

• Pneumonia

• Atelectasis

• Pulmonary edema

• Cerebral infarction

• Postoperative delirium

• Dementia

• Intestinal Obstruction

• Pseudomembranous Colitis

• Gastrointestinal Perforation/Gastric Perforation

• Pancreatitis

• Hepatitis

• Cholangitis

• Acute Renal Failure

• Urinary Tract Infection

• DIC

• Sepsis

• Fractures Due to Falls During Hospitalization

• Other

3) Late Postoperative Complications

• Neurological symptoms

• Worsening of local kyphosis

• Worsening of slippage

• Systemic complications/comorbidities

• Instrument-related (loosening/dislocation)

• Infection (superficial/deep)

• Other

**9.3　Response to Adverse Events, Surgical Complications, and Malfunctions**

**9.3.1　Response to Adverse Events, Surgical Complications, and Malfunctions**

When the attending surgeon identifies an adverse event (including surgical complications; the same applies hereafter) or malfunction, they shall immediately take appropriate measures and record the details accurately in the original source documents (e.g., medical history records) and the case report form without discrepancy. Furthermore, if protocol treatment is discontinued or treatment for the adverse event becomes necessary, the subject must be informed accordingly (refer to “15. Matters Concerning Monetary Payments and Compensation” regarding compensation for adverse events).

**9.3.2　Response to Serious Adverse Events, Surgical Complications, and Malfunctions**

For all serious adverse events (including surgical complications; the same applies hereafter) and any serious adverse events suspected to be related to the study occurring after study completion (termination), appropriate treatment must be promptly administered to the research subject, and the principal investigator must be notified. The principal investigator must promptly report to the head of the research institution (hospital director) using the designated form ([Safety Report Form 2], etc.) (in principle, the initial report within two weeks) and report to the Ethics Review Committee or Clinical Research Oversight Committee*. Notify all heads of research institutions (hospital directors) promptly as well.

*: Report via Tokyo University of Science's “Ethics Review Application System”.

**9.3.3　Response to Unforeseen Serious Adverse Events, Surgical Complications, or Malfunctions**

When unforeseen serious adverse events (including surgical complications; the same applies below) occurs, promptly administer appropriate treatment to the research subject and report to the Principal Investigator. The Principal Investigator shall promptly (principally within two weeks for the initial report) report to the head of the research institution (hospital director) using the prescribed form ([Form An1], etc.), and report to the Ethics Review Committee or Clinical Research Monitoring Committee. Notify the head of all research institutions (hospital director).

*: Report via Tokyo University of Science's “Ethics Review Application System”.

※ Events described in “9.2 Anticipated Adverse Events” are considered known/predictable events.

## **9.4　Observation of Research Subjects Following Adverse Events, Surgical Complications, or Malfunctions**

Research subjects shall be observed following the occurrence of an adverse event (including surgical complications; the same applies hereafter) until the adverse event resolves or recovers, or until the attending surgeon determines that follow-up is unnecessary.

**10．Matters Concerning Statistical Analysis**

An overview of the statistical analysis plan for this study is provided below. Details of the statistical analysis plan are specified separately in the Statistical Analysis Plan. While the Statistical Analysis Plan may alter analysis methods, etc., from the overview in this Study Protocol, if changes significantly impact the interpretation of this clinical study's results—such as alterations to the definition of primary endpoints or analysis methods—this Study Protocol shall be revised.

**10.1　Analysis Population**

**10.1.1　Full Analysis Set (FAS)**

This population is defined as the group of cases enrolled and randomized in this study who received the protocol treatment (cervical laminoplasty). However, cases with no data whatsoever after randomization will be excluded.

**10.1.2　Safety Analysis Set (SAS)**

This is defined as the population of all cases enrolled and randomized in this study who underwent the protocol treatment (cervical laminoplasty).

**10.2　Target Enrollment Numbers and Rationale**

Target enrollment: 216 cases

(Control group [mini-plate]: 108 cases, Test treatment group [suture-anchor]: 108 cases)

[Rationale]

Regarding the primary endpoint, the recovery rate in cervical JOA scores before and after surgery, a retrospective pilot study at Saiseikai Kawaguchi General Hospital, which used a patient population similar to this study, reported rates of 49.9±22.8% (21 cases) for the plate method and 52.6±32.9% (20 cases) for the suture anchor method (unpublished data).

Furthermore, a previous report^3)^ indicated that the MCID for compressive myelopathy is 52.8%. Based on the pilot study and past MCID findings, the recovery rate in cervical JOA scores for the control group (plate method) in this study was set at 50% (standard deviation 40%), with a non-inferiority margin of 20%. With a significance level (one-sided) of 0.025 and

and a power of 0.9, the required sample size for both groups combined is 172 cases. Anticipating approximately 20% dropout, the target sample size for both groups combined was set at 216 patients.

This target enrollment number is treated as an anticipated enrollment number, and exceeding this anticipated number is permitted. However, the Principal Investigator (PI) shall manage the cumulative actual enrollment numbers across all participating institutions to ensure the total number of cases does not exceed the study's target enrollment. If additional enrollment is required to meet the overall study target, prior approval must be obtained from the Ethics Review Committee or equivalent body.

**10.3　Case Handling**

In principle, decisions regarding the handling of enrolled cases shall be made by the Principal Investigator and the Statistician, after consultation. Decisions on case handling arising from new issues shall also be made by the Principal Investigator and the Statistician, after consultation. In all cases, the details of the case handling decisions shall be documented.

**10.4　Data Handling**

Data handling during data collection and analysis shall generally follow the principles outlined below. In case of doubt, the principal investigator and the person responsible for statistical analysis shall consult and decide. Missing values shall not be imputed. Details shall be specified separately in the statistical analysis plan.

**10.5　Statistical Analysis Items and Analysis Plan**

All efficacy evaluations will be conducted using the FAS population.

Safety analyses will be conducted using the SAS population.

Details of the statistical analysis will be specified in a separate statistical analysis plan created before data locking.

**10.5.1　Summary of Subject Background**

The distribution and summary statistics of subject background data will be calculated for each analysis population, by allocation group. For nominal and ordinal variables, the frequency and proportion of categories will be shown by allocation group. For continuous variables, summary statistics (number, mean, standard deviation, minimum, median, maximum) will be calculated by allocation group.

**10.5.2　Analysis of Primary Endpoints**

・Recovery Rate in Cervical JOA Score Before and After Surgery

For FAS participants, calculate the least squares mean and its 95% confidence interval for the cervical JOA score recovery rate at 1 year post-surgery using analysis of covariance. Also calculate the between-group difference in least squares means (suture anchor method vs. plate method), its 95% confidence interval, and the p-value. The analysis model included the following covariates: group, preoperative JOA score, age (≤64 years, 65–74 years, ≥75 years), sex, and preoperative diagnosis (cervical spondylotic myelopathy (CSM), ossification of the posterior longitudinal ligament (OPLL)).

The suture-anchor method will be considered non-inferior to the mini-plate method if the lower limit of the 95% confidence interval for the least squares mean difference between groups at 1 year postoperatively exceeds –20%. Additionally, as a secondary analysis, a Mixed-effects Model for Repeated Measures (MMRM) analysis will be performed using the first available subject (FAS) population, with the recovery rate in cervical JOA scores at 1 year and 2 years postoperatively relative to preoperatively as the response variable. The analysis model will include the following as fixed effects: group, time point, group-time interaction, preoperative JOA score, age (≤64 years, 65–74 years, ≥75 years), sex, and preoperative diagnosis (CSM, OPLL). Using this model, the least squares mean and 95% confidence interval for the recovery rate in cervical JOA scores before and after surgery at 1 year and 2 years postoperatively will be calculated. Additionally, the between-group differences in the recovery rate of cervical JOA scores before and after surgery at 1 year and 2 years postoperatively, along with their 95% confidence intervals and p-values, were calculated.

**10.5.3　Secondary Outcome Analysis**

(1) Operative time

(2) Estimated blood loss

For FAS, calculate summary statistics (sample size, mean, standard deviation, minimum, median, maximum) by group. Perform Student's t-test for between-group comparisons and calculate p-values.

(3) Proportion achieving the minimal clinically important difference (MCID) in cervical JOA score recovery rate at postoperative 1 and 2 years

For the FAS, calculate the proportion achieving the MCID (JOA score recovery rate of 52.8%) for the cervical JOA score recovery rate at postoperative 1 year and 2 years by group. For between-group comparisons, calculate the difference in the proportion achieving the MCID (52.8% recovery rate) and its 95% confidence interval, then perform Fisher's exact test. Use the Clopper-Pearson method to calculate the confidence interval.

(4) Health-related QOL (EQ-5D) at postoperative 1 and 2 years

(5) Severity of neck pain, upper limb pain, and upper limb numbness (VAS) at postoperative 1 and 2 years

(6) Neck disability index (NDI) at postoperative 1 and 2 years compared to preoperative

For FAS, perform analyses similar to those for primary endpoints on the change in each assessment item from preoperative to 1 year postoperatively.

Additionally, perform analyses similar to those for secondary endpoint (2) on the change in each assessment item from preoperative to 1 year and 2 years postoperatively.

(7) Retention rate of enlarged lamina at postoperative 1 and 2 years (Retention rate)

For FAS, the change in the retention rate of enlarged lamina from preoperatively to 1 year postoperatively will be analyzed similarly to the primary endpoint.

Furthermore, the change in the retention rate of enlarged lamina from preoperatively to 1 and 2 years postoperatively will be analyzed similarly to secondary endpoint (2).

(8) Percentage of hinge fractures at postoperative 1 and 2 years

(9) Percentage of bone union of gutter at postoperative 1 and 2 years

For FAS, calculate the percentage for each evaluation item at 1 year and 2 years postoperatively by group. For between-group comparisons, calculate the difference in the percentage for each evaluation item and its 95% confidence interval, and perform Fisher's exact test. Use the Clopper-Pearson method to calculate the confidence interval.

　(10) Sagittal alignment of cervical spine at postoperative 1 and 2 years (C-SVA, C2-7 angle, T1 slope)

For FAS subjects, perform analyses similar to those for primary endpoints on the change in each evaluation item from preoperatively to 1 year postoperatively.

Additionally, perform analyses similar to those for secondary endpoint (2) on the change in each evaluation item from preoperatively to 1 and 2 years postoperatively.

(11) Paraspinal muscle cross-sectional area (C4/5 level) at postoperative 1 and 2 years

For FAS, perform the same analysis as for primary endpoints on the change in paraspinal muscle cross-sectional area from preoperatively to 1 year postoperatively.

Additionally, perform the same analysis as for secondary endpoint (2) on the change in paraspinal muscle cross-sectional area from preoperatively to 1 and 2 years postoperatively.

(12) Dural sac cross-sectional area (C3/4, C4/5, C5/6, C6/7 levels) at postoperative 1 and 2 years

For FAS subjects, perform the same analysis as for the primary endpoint on the change in dural canal area from preoperative to 1-year and 2-year postoperative time points.

Additionally, perform the same analysis as for secondary endpoint (2) on the change in dural canal area from preoperative to 1-year and 2-year postoperative time points.

(13) Grading of the mass posterior to the dural sac at postoperative 1 and 2 years

For FAS subjects, classify posterior spinal canal compression at 1 and 2 years postoperatively into mild (Grade 1-3) and severe (Grade 4-5). Calculate the proportion of severe posterior spinal canal compression at 1 and 2 years postoperatively by group. For between-group comparisons, calculate the difference in the proportion for each evaluation item and its 95% confidence interval, then perform Fisher's exact test. Use the Clopper-Pearson method to calculate the confidence interval.

(14) Incremental Cost-Effectiveness Ratio (ICER)

Calculate the ICER of the test treatment group relative to the control group.

Refer to Section 8.1.2 for details on the calculation method.

(15) Surgical complication incidence

For SAS, calculate the number of cases and rates of each complication by the following categories for each assigned group.

・Intraoperative complication rate (from start of surgery to end of surgery (wound closure))

・Early postoperative complication rate (from end of surgery (wound closure) to within 30 days postoperatively)

・Late postoperative complication rate (from postoperative day 31 to within 2 years postoperatively)

**10.5.4　Subgroup Analysis**

Subgroup analyses will be performed based on the factors listed below. These analyses are not designed to ensure sufficient power and will not include multiple testing adjustments; therefore, the results of each subgroup analysis should be interpreted as exploratory findings only. Additionally, subgroup analyses related to other clinical-pathological characteristics or prognostic factors expected to have clinical significance at the time of analysis will be performed.

<Factors Planned for Subgroup Analysis>

・ Age (64 years or younger / 65-74 years / 75 years or older)

・ Gender (Male / Female)

・ BMI (≤25 / >25)

・ Preoperative Diagnosis (CSM / OPLL)

・ Number of Expanded Laminas (2 / 3 / 4)

・ Posterior spinal canal compression classification (Grade 1-3 / Grade 4-5)

**10.6　Interim Analysis**

No interim analysis will be conducted.

**10.7　Primary Analysis**

The primary analysis for the primary endpoint and secondary efficacy endpoints will be performed using data at postoperative 1 year. The primary analysis will be conducted after completing the 1-year post-surgery evaluation for the last enrolled case.

Details will be specified in a separate statistical analysis plan created prior to data locking.

**10.8　Final Analysis**

The follow-up period will end upon completion of the 2-year postoperative evaluation for the last enrolled case. The final analysis will be performed after the follow-up period ends and data from all cases are locked. The person responsible for statistical analysis will compile the “Analysis Report” and submit it to the Principal Investigator.

**11．Matters Concerning Access to Original Data and Related Materials**

Original documents in this study include medical records, various test data, surgical records, medication records, patient evaluation sheets, etc.

The Principal Investigator and the research institution administrator shall accept monitoring and audits related to this study, as well as investigations by the Ethics Review Committee and regulatory authorities, and shall guarantee direct access to all original documents and other materials related to this study during such events.

For data recorded in case report forms but not documented in medical records, the following items shall use the case report form entries as the source data:

1) Severity, degree, outcome, date of outcome, causal relationship with protocol treatment, etc., of complications/adverse events, and the rationale for determining causality

2) Date of discontinuation, reason for discontinuation, complications/adverse events causing discontinuation, post-discontinuation course, and results of follow-up

3) Comment from the responsible attending surgeon

**12．Matters Concerning Quality Control and Quality Assurance**

**12.1　Monitoring**

The Principal Investigator shall conduct monitoring to confirm that the study is being conducted safely and according to the protocol, and that data are being collected accurately. The Principal Investigator shall also designate a monitoring officer responsible for conducting the monitoring. The monitor shall verify throughout the study period that the research complies with the latest protocol and relevant research guidelines. The monitor shall prepare a monitoring report detailing these findings and submit it to the Principal Investigator. The Principal Investigator shall review the monitoring report, share identified issues with the research team at the institution, and strive to implement improvements. The monitor must not disclose any personal information of research subjects obtained during monitoring.

① Registration Status: Number Registered - Cumulative/By Period, By Group/By Site

② Eligibility: Ineligible Cases/Potentially Ineligible Patients: By Group/By Site

③ Pre-Treatment Baseline Factors: By Group

④Protocol Treatment Status: On Treatment/Off Treatment, Reason for Discontinuation/Completion: By Group/By Site

⑤ Protocol Deviations: By Group/By Site

⑥ Serious Adverse Events: Group/Site

⑦ Adverse Events/Incidents: Group

⑧ Overall Survival: All Registered Cases

⑨ Other Issues Regarding Trial Progress or Safety

**12.2　Audit**

As this study is a comparative trial of surgical techniques currently performed under insurance-covered medical care, researchers and others will ensure quality, and no audit will be conducted. However, if events significantly impacting subject safety occur, etc., conducting an audit will be considered as necessary.

# **13．Ethical Considerations**

**13.1　Compliance with Laws and Regulations**

All researchers involved in this study will conduct the research in compliance with the “Declaration of Helsinki” (October 2013, Japanese Medical Association translation)^A)^, the “Ethical Guidelines for Life Science and Medical Research Involving Human Subjects” (partially revised March 10, 2022)^B)^, and related notifications.

A) http://dl.med.or.jp/dl-med/wma/helsinki2013j.pdf

B) https://www.mhlw.go.jp/content/000909926.pdf

**13.2　Expected Benefits, Burdens, and Harms**

1) Expected Benefits

Participation in this study will not directly benefit the research subjects. The research findings may contribute to future advances in medical care.

2) Expected Disadvantages

Regarding the comparison of technical difficulty between the control treatment (plate method) and the experimental treatment (suture anchor method), no difference is expected when performed by surgeons skilled in spinal surgery. Furthermore, no difference is anticipated in complication rates, safety, or other aspects. Indeed, a meta-analysis of short-term outcomes for plate and suture anchor methods in open laminectomy performed overseas reported no significant difference in operating time, blood loss, or JOA scores23. Therefore, we consider there to be no particular disadvantage associated with participating in this study.

Furthermore, participation in this study requires that a Japanese Spine Society Spine Surgery Instructor be employed at each participating institution. This ensures surgical quality and safety, minimizes risks for research subjects, and enables high-quality clinical research.

3) Expected Burden

The duration of hospitalization, number of visits, number of tests, and time spent on examinations/tests associated with participating in this study are comparable to routine clinical care. Furthermore, research subjects will incur no financial burden from participating in this study (see “15. Matters Concerning Monetary Payments and Compensation”).

4) Measures to Minimize Risk

Since this study enrolls patients who require laminoplasty and were already scheduled for surgery, the likelihood of physical risks exceeding those of routine care due to study participation is considered low. However, throughout the follow-up period, the attending surgeon will closely monitor the subject's condition and strive for early detection of surgical complications and adverse events.

Should complications or adverse events arise due to protocol treatment, responses will be considered on a case-by-case basis. For considerations regarding the protection of research subjects' personal information, refer to “21. Handling of Personal Information, etc.”

**13.3　Handling of Research Findings and Incidental Findings Related to Genetic Characteristics of Research Subjects**

This study will not perform any tests or analyses that could yield significant findings regarding the health or genetic characteristics of research subjects.

# **14．Matters Concerning Collection, Storage, and Disposal of Information and Samples**

**14.1****Collection, Storage, and Disposal of Information**

Information collected (research data) in this study will be entered into and managed by the electronic data capture (EDC) system “eACReSS”. Entered information will be stored on the eACReSS server at Institute of Science Tokyo Hospital.

*: “eACReSS” is a dedicated system for managing clinical research data, developed under the University Hospital Clinical Trial Alliance Project.

① Storage Location: Institute of Science Tokyo Hospital “eACReSS” server

※ After the trial concludes, research data will be transferred to the Principal Investigator using optical discs or similar media.

② Storage Responsible Person: Toshitaka Yoshii (Principal Investigator)

③ Retention Period: At least 10 years after publication of the main paper, etc. (in accordance with university regulations)

④ Disposal Method: If disposed of after the retention period ends, it shall be processed into an unrecoverable state before disposal.

⑤

Potential for secondary use: ■ Yes　　　□ No

Furthermore, paper documents and source materials (e.g., medical history records, worksheets) related to research implementation, other than those entered into the EDC system, shall be stored in lockable storage units at each research institution.

① Storage Responsible Person: Research Principal Investigator at each research institution

② Retention Period: 10 years after study completion

③ Disposal Method: Shred or otherwise process into an irrecoverable state before disposal.

Furthermore, data obtained from research subjects in this study may potentially be used for future research. Should such future research be conducted, a separate research protocol must be prepared and reviewed by the Ethics Review Committee or equivalent body before implementation (see “21.2 Secondary Use of Data”).

**14.2** **Sample Collection, Storage, and Disposal**

This study does not plan to collect or use blood, tissue, cells, body fluids, excretions, or DNA extracted from these samples from research subjects.

**15．Matters Concerning Monetary Payments and Compensation**

**15.1** **Monetary Payments (Research Subject Expenses)**

The protocol treatment in this study (laminoplasty using the plate method or suture anchor method) falls within the scope of insurance-covered medical treatment. Participation in this study will not result in any additional costs for research subjects. Any costs incurred by research subjects will be within the range of costs typically incurred during routine medical care.

**15.2** **Compensation**

Although this study does not involve medical procedures beyond the scope of routine medical care, it is covered by clinical research insurance. Should unknown side effects or similar occur, necessitating treatment equivalent to hospitalization, medical expenses and medical allowances will be covered by the enrolled clinical research insurance. For other health damages arising from the conduct of this study, appropriate measures will be taken, such as providing medical care. Furthermore, to prepare for health damages to research subjects arising from routine medical procedures within this study, surgeons participating in the study must be enrolled in individual insurance.

**16．Information Disclosure**

- Prior to conducting this research, the research content will be registered in the clinical research database (jRCT Clinical Research Submission and Disclosure System (https://jrct.niph.go.jp/)).
- Research findings will be published at domestic and international research meetings, academic conferences, in papers, etc., but will be disclosed in a form that does not contain information that could identify research subjects.。

**17．Implementation Period**

Total Research Period: October 17, 2025 (jRCT Publication Date) to March 31, 2031 (5.5 years)

(Planned Registration Period: 3 years, Follow-up Period: 2 years post-surgery, Analysis Period: 0.5 years)

**18．****Explanation and Consent for Research Participants**

Written informed consent based on free will will be obtained from all research participants. Consent will be documented by the participant's signature on the consent form. The explanatory document and consent form approved by the same ethics review committee that approved this research protocol will be used.

Research participants may freely refuse to participate in the study or withdraw from it at any time, even without a valid reason. Participants will not suffer any disadvantage due to refusal or withdrawal.

After the attending surgoen (or a research collaborator designated by the principal investigator) provides appropriate and important information and explains it fully using plain language, both the research subject and the consent acquirer (the attending surgeon) shall personally sign and date the consent form. If a research collaborator provides supplementary explanations, that collaborator shall also sign and date the form. The research subject receives a copy of the explanatory document and the signed consent form, while the original is retained by the research institution.

If the research protocol is revised and research procedures are significantly added or changed, the research subject may be asked to sign an additional consent form.

《Explanatory Items for Research Subjects》

The consent explanation document shall include the following items:

① The name of the research and that the head of the research institution has approved its implementation

② The name of the research institution and the name of the principal investigator (if a multi-institution collaborative study is conducted, this includes the names of the collaborating institutions and their respective principal investigators)

③ The purpose and significance of the research

④ The research methods (including the purpose of use and handling of samples/information obtained from research subjects) and duration

⑤ The reason for selection as a research subject

⑥ The burden on research subjects, as well as the anticipated risks and benefits

⑦ A statement that consent to the conduct or continuation of the research may be withdrawn at any time (including a statement and reason if it may be difficult to take measures in accordance with the content of the withdrawal from research subjects, etc.)

⑧ A statement that research subjects, etc., will not be subject to disadvantageous treatment by refusing to consent to the conduct or continuation of the research or by withdrawing consent

⑨ Method for disclosing information about the research

⑩ Upon request by research subjects, they may obtain or view the research protocol and materials concerning research methods, to the extent that this does not compromise the protection of other research subjects' personal information or the originality of the research, and the method for obtaining or viewing such materials

⑪ Handling of personal information (including the method if measures are taken to prevent identification of individuals, and a statement if pseudonymized or anonymized information is created)

⑫ Methods for Storing and Disposing of Samples and Information

⑬ Sources of Research Funding and Other Conflicts of Interest Related to the Research Institution's Research, and Situations Regarding Personal Income or Other Conflicts of Interest Related to the Researcher's Research

⑭ Handling of Results Obtained Through the Research

⑮ Response to Inquiries from Research Subjects and Related Parties (Including Genetic Counseling)

⑯ If there is any financial burden or compensation for research subjects, etc., the fact and its details

⑰ For research involving medical procedures beyond routine clinical practice, matters concerning other treatment methods, etc.

⑱ For research involving medical procedures beyond routine clinical practice, arrangements for providing medical care to research subjects after the research is conducted

⑲ For invasive research: Whether compensation is provided for health damage caused by the research and its details

⑳ For samples/information obtained from research subjects: If there is a possibility they may be used for future research not specified at the time consent is obtained or provided to other research institutions, the fact and the content anticipated at the time consent is obtained

㉑ For research involving invasive procedures (excluding minor invasions) that includes interventions, the fact that persons engaged in monitoring and auditing, as well as the ethics review committee, will access samples and information pertaining to the research subjects within the necessary scope, provided that the confidentiality of the research subjects is preserved

**19．Matters Concerning Conflicts of Interest**　

This research is conducted using research funds from the Department of Orthopedic Surgery, Institute of Science Tokyo (operating funds and joint research funds with Olympus Terumo Biomaterials Corporation, etc.). There is no provision of funds or benefits from manufacturers or distributors of surgical instruments. Researchers independently plan, conduct, analyze results, and publish findings without corporate influence. Conflicts of interest involving researchers in this study are managed in accordance with the conflict of interest management systems (e.g., conflict of interest committees) of each participating research institution. Any changes to disclosed conflicts of interest will be reported to the relevant committee.

**20．Intellectual Property Rights**

Results, data, and intellectual property rights obtained through this research belong to the Principal Investigator (or the research institution conducting this study). Specific handling and distribution shall be determined through consultation.

**21．Handling of Personal Information**

**21.1** **Protection of Personal Information**

When handling data and consent documents related to this research, sufficient consideration shall be given to protecting the personal information of research subjects. Case reports and similar documents shall be recorded using identification codes. When publishing the results of this research, information that could identify research subjects shall be excluded. Information and data collected in this study shall be processed by removing personal information such as names, initials, and patient IDs, and assigning new codes to prevent individual identification. Furthermore, the management of this information shall be handled appropriately in accordance with Section 14. Collection, Storage, and Disposal of Information and Samples. If separate regulations or procedures regarding personal information protection exist within the research institution, they shall also be followed appropriately.

**21.2** **Secondary Use of Data**

Data obtained in this study may be used for secondary purposes (ancillary research) after obtaining renewed approval from the Ethics Review Committee or similar body. Such data may be retained beyond the period specified for record retention. In such cases, personal information of research subjects will be protected using the same methods as described in Section 21.1 “Protection of Personal Information.”

**22．Compliance with the Research Protocol and Protocol Amendments**

**22.1** **Compliance with the Research Protocol**

Researchers involved in this study shall conduct the research in compliance with the research protocol, provided it does not compromise the safety or human rights of research subjects.

**22.2** **Changes to the Research Protocol**

If changes to the research protocol (including the explanatory document and consent form) become necessary after the start of this research, the Principal Investigator shall submit the proposed changes and their rationale for re-review and approval by the Ethics Review Committee. After approval, the Principal Investigator shall notify the head of the research institution (hospital director) of the changes according to the institution's established procedures and inform the Principal Investigators at other research institutions. The Principal Investigator receiving this information shall report the changes to the institution's administrator or obtain the administrator's implementation approval according to the institution's procedures. All Principal Investigators and responsible srgeons must not conduct research using the modified research protocol (including the explanatory document and consent form) before obtaining approval from the Ethics Review Committee or the head of the research institution (hospital director).

**23．Handling of Deviations (Non-Compliance) from the Research Protocol**

• The principal investigator and the attending surgeon shall not deviate from or modify the research protocol without obtaining the prior agreement of the research representative and the prior review by the ethics review committee, followed by approval from the head of the research institution (hospital director).

• The Principal Investigator and the attending surgeon may deviate from or change the research protocol before obtaining prior approval from the Ethics Review Committee due to unavoidable reasons such as emergency avoidance. In such cases, the Principal Investigator and the attending surgeon shall promptly submit the details and reasons for the deviation or change, along with any necessary draft revisions to the research protocol, to the Ethics Review Committee and obtain approval from both the Ethics Review Committee and the head of the research institution (hospital director).

• The Principal Investigator and the attending surgeon must record all deviations from the research protocol, including the reasons for each deviation.

• If particularly serious non-compliance is identified, the Principal Investigator and the attending surgeon shall promptly report it to the head of the research institution (hospital director) and notify the Research Representative.

The Principal Investigator shall report to the Ethics Review Committee, etc., and take necessary actions. The Principal Investigator shall also promptly provide information to other Principal Investigators, etc.

**24．Reporting to the Head of the Research Institution and Method**

During the research period, the Principal Investigator and Principal Investigators shall report on the status of research implementation to the Ethics Review Committee and the head of the research institution (hospital director) once a year.

Furthermore, the principal investigator and research supervisors shall promptly report in writing to the Ethics Review Committee and the head of the research institution (hospital director) any content falling under the following items:

• Facts or information that compromise the ethical validity or scientific rationality of the research, or information that may compromise them and is considered to affect the continuation of the research

• Facts or information that compromise the appropriateness of the research implementation or the reliability of the research results, or information that may compromise them

• Occurrence of a serious adverse event

• Progress of the research, occurrence of adverse events, or completion (including discontinuation) of the research

**25．Discontinuation of Research**

• If the Principal Investigator decides to discontinue or suspend the research, they shall promptly report this decision and its reasons to both the Ethics Review Committee and the head of the research institution (hospital director).

• If discontinuation is ordered due to a serious non-compliance with the “Ethical Guidelines for Life Science and Medical Research Involving Human Subjects,” the head of the research institution (hospital director) shall report to the Minister of Health, Labour and Welfare. Research subjects shall promptly discontinue protocol treatment.

＊: Report via Institute of Science Tokyo “Ethics Review Application System.”

**26．Research Termination**

- Research is considered terminated upon completion of the statistical analysis report. When research is terminated, report simultaneously to the Ethics Review Committee and the head of the research institution (hospital director), and provide information to the principal investigators at other research institutions. Principal investigators receiving this information shall report it to their respective institution's administrator.

※ Report via Institute of Science Tokyo “Ethics Review Application System”.

- The principal investigator shall also report the completion of the research to the jRCT Clinical Research Submission and Disclosure System.

**27．References**

1. Nouri A, Tetreault L, Singh A, Karadimas SK, Fehlings MG. Degenerative Cervical Myelopathy: Epidemiology, Genetics, and Pathogenesis. Spine (Phila Pa 1976) 2015;40:E675-93.

2. Nagata K, et al. The prevalence of cervical myelopathy among subjects with narrow cervical spinal canal in a population-based magnetic resonance imaging study: the Wakayama Spine Study. Spine J 2014; 14(12): 2811-2817.

3. Japanese Orthopaedic Association, Japanese Society for Spine Surgery and Related Research. Cervical Spondylotic Myelopathy: Clinical Practice Guidelines 2020, Revised 3rd Edition. P35-37

4. Hirabayashi K, Watanabe K, Wakano K, Suzuki N, Satomi K, Ishii Y. Expansive open-door laminoplasty for cervical spinal stenotic myelopathy. Spine (Phila Pa 1976) 1983;8:693-9.

5. Miyazaki K, Kirita Y. Extensive simultaneous multisegment laminectomy for myelopathy due to the ossification of the posterior longitudinal ligament in the cervical region. Spine (Phila Pa 1976) 1986;11:531-42.

6. Japanese Orthopaedic Association, Japanese Society for Spine and Spinal Cord Disorders (Supervising Editors). Cervical Spondylotic Myelopathy: Clinical Practice Guidelines 2020, Revised 3rd Edition. P48-54

7. Sakai K, Hirai T, Arai Y, Maehara H, Torigoe I, Inose H, Tomori M, Sakaki K, Yuasa M, Matsukura Y, Oyaizu T, Morishita S, Yoshii T, Okawa A. Laminar Closure in Double-door Laminoplasty for Cervical Spondylotic Myelopathy with Nonkyphotic Alignment. Spine (Phila Pa 1976) 2021;46:999-1006.

8. Fujishiro T, Nakano A, Baba I, Fukumoto S, Nakaya Y, Neo M. Double-door cervical laminoplasty with suture anchors: evaluation of the clinical performance of the constructs. Eur Spine J 2017;26:1121-8.

9. Taniyama T, Hirai T, Yamada T, Yuasa M, Enomoto M, Yoshii T, Kato T, Kawabata S, Inose H, Okawa A. Modified K-line in magnetic resonance imaging predicts insufficient decompression of cervical laminoplasty. Spine (Phila Pa 1976) 2013;38:496-501.

10. Hirai T, Okawa A, Arai Y, Takahashi M, Kawabata S, Kato T, Enomoto M, Tomizawa S, Sakai K, Torigoe I, Shinomiya K. Middle-term results of a prospective comparative study of anterior decompression with fusion and posterior decompression with laminoplasty for the treatment of cervical spondylotic myelopathy. Spine (Phila Pa 1976) 2011;36:1940-7.

11. Yoshiyama T, Fujibayashi S, Otsuki B, Shimizu T, Murata K, Matsuda S. Preoperative and Postoperative Factors Affecting Patient Satisfaction with Double-Door Laminoplasty for Cervical Spondylotic Myelopathy. Spine Surg Relat Res 2023;7:421-7.

12. Hoshi K, Kurokawa T, Nakamura K, et al. Expansive cervical laminoplasties--observations on comparative changes in spinous process lengths following longitudinal laminal divisions using autogenous bone or hydroxyapatite spacers. Spinal Cord 1996;34:725-8.

13. Kaito T, Hosono N, Makino T, et al. Postoperative displacement of hydroxyapatite spacers implanted during double-door laminoplasty. J Neurosurg Spine 2009;10:551-6.

14. Kimura A, Seichi A, Inoue H, Hoshino Y. Long-term results of double-door laminoplasty using hydroxyapatite spacers in patients with compressive cervical myelopathy. Eur Spine J 2011;20:1560-6.

15. Takeoka Y, Yurube T, Maeno K, et al. Improved bone bonding of hydroxyapatite spacers with a high porosity in a quantitative computed tomography-image pixel analysis: A prospective 1-year comparative study of the consecutive cohort undergoing double-door cervical laminoplasty. JOR Spine 2020;3:e1080.

16. Park HG, Zhang HY, Lee SH. Box-shape cervical expansive laminoplasty: clinical and radiological outcomes. Korean J Spine 2014;11:152-6.

17. Oh CH, Ji GY, Hur JW, Choi WS, Shin DA, Lee JB. Preliminary Experiences of the Combined Midline-Splitting French Door Laminoplasty with Polyether Ether Ketone (PEEK) Plate for Cervical Spondylosis and OPLL. Korean J Spine 2015;12:48-54.

18. Wu W, Zhang S, Yan T. Initial clinical experiences of the muscle-preserving double door cervical laminoplasty with adjustable mini plates. Front Surg 2022;9:1049937.

19. Kato S, Oshima Y, Matsubayashi Y, et al. Minimum Clinically Important Difference and Patient Acceptable Symptom State of Japanese Orthopaedic Association Score in Degenerative Cervical Myelopathy Patients. Spine 2019;44:691-7.

20. Okada E, Matsumoto M, Ichihara D, et al. Cross-sectional area of posterior extensor muscles of the cervical spine in asymptomatic subjects: a 10-year longitudinal magnetic resonance imaging study. Eur Spine J 2011;20:1567-73.

21. Ratliff JK, Cooper PR. Cervical laminoplasty: a critical review. J Neurosurg 2003;98:230-8.

22. Takeuchi K, Yokoyama T, Wada KI, et al. A New Grading of Epidural Hematoma or Scar Formation after Posterior Cervical Spine Surgery: Evaluation of Perioperative Related Factors, Distributions, and Clinical Outcomes after Surgery. Spine Surg Relat Res 2019;3:285-94.

23. Mo Z, Li D, Zhang R, et al. Comparison of three fixation modalities for unilateral open-door cervical laminoplasty: a systematic review and network meta-analysis. Neurosurg Rev 2020;43:813-23.
